# Foxi1 regulates multipotent mucociliary progenitors and ionocyte specification through transcriptional and epigenetic mechanisms

**DOI:** 10.1371/journal.pbio.3003583

**Published:** 2026-01-05

**Authors:** Sarah Bowden, Magdalena Maria Brislinger-Engelhardt, Mona Hansen, Aisha Andricek, Africa Temporal-Plo, Damian Weber, Sandra Hägele, Fabian Lorenz, Tim Litwin, Clemens Kreutz, Peter Walentek

**Affiliations:** 1 Internal Medicine IV, Medical Center—University of Freiburg, Freiburg, Germany; 2 CIBSS Centre for Integrative Biological Signalling Studies, University of Freiburg, Freiburg, Germany; 3 IMPRS-EBM International Max Planck Research School of Epigenetics, Biophysics, and Metabolism, Max Planck Institute of Immunobiology and Epigenetics, Freiburg, Germany; 4 SGBM Spemann Graduate School for Biology and Medicine, University of Freiburg, Freiburg, Germany; 5 IMBI Institute of Medical Biometry and Statistics, Medical Center—University of Freiburg, Freiburg, Germany; California Institute of Technology, UNITED STATES OF AMERICA

## Abstract

Foxi1 is a master regulator of ionocytes (ISCs/INCs) across species and organs. Two subtypes of ISCs exist, and both α- and β-ISCs regulate pH- and ion-homeostasis in epithelia. Gain and loss of FOXI1 function are associated with human diseases, including Pendred syndrome, male infertility, renal acidosis, and cancers. Foxi1 was predominantly studied in the context of ISC specification, however, reports indicate additional functions in early and ectodermal development. Here, we re-investigated the functions of Foxi1 in *Xenopus laevis* embryonic mucociliary epidermis developpment and found a novel function for Foxi1 in the generation of Notch-ligand expressing mucociliary multipotent progenitors (MPPs). We demonstrate that MPPs are a distinct sub-population of epidermal cells in which Foxi1 has two concentration-dependent functions: At low levels, Foxi1 maintains ectodermal competence in MPPs through transcriptional and epigenetic mechanisms, while at high levels, Foxi1 induces a multi-step process of ISC specification and differentiation in cooperation with Ubp1 and Dmrt2. We further describe how *foxi1* expression is affected through auto- and Notch-regulation, and how this developmental program affects mucociliary patterning. Together, we reveal novel functions for MPPs and Foxi1 in *Xenopus* mucociliary epidermis formation, relevant to our understanding of vertebrate development and human disease.

## Abstract 2

The transcription factor Foxi1 regulates ionocytes across species and organs and it is associated with several human diseases, but its function in epidermis remains unclear. This study shows that low concentrations of Foxi1 in progenitor cells maintain epidermal identity via the Notch pathway affecting mucociliary cell fates in Xenopus.

## Introduction

The Forkhead-box transcription factor Foxi1 is a master regulator of ionocytes (ISCs) in the vertebrate lung, kidney, inner ear, and epididymis [1,2] as well as in the embryonic skin of aquatic species (e.g., zebrafish and *Xenopus* frogs) [3,4]. In all these tissues, ISCs regulate ion homeostasis through the expression of transmembrane solute carriers and pH-regulators (e.g., vacuolar (v)H^+^ATPase encoded by *atp6* genes, Pendrin encoded by *slc26a4*, and Anion exchanger 1 encoded by *slc4a1*) [3]. In *Xenopus* embryos, an additional role for Foxi1 has been described during germ layer specification, where Foxi1 promotes epidermis formation by activating ectodermal gene expression while simultaneously counteracting vegetal mesendoderm-inducing factors (e.g., VegT) [5–7]. However, how Foxi1 can have such profoundly different functions has not been elucidated so far.

The *Xenopus* embryonic epidermis is a popular model to study vertebrate mucociliary epithelia [8,9]. Mucociliary epithelia in the mammalian lung and the *Xenopus* epidermis serve as first line of defense against pathogens through mucociliary clearance [10]. They are composed of secretory cells (e.g., goblet cells) that release mucus and anti-microbial peptides, and multiciliated cells (MCCs) that generate a fluid flow by means of directional cilia beating to remove pathogens [11]. Mucociliary ISCs are important for efficient mucociliary clearance [12,13], and Foxi1 dysregulation is linked to a range of human diseases affecting airway and kidney function as well as to causing deafness and cancers [2,14–20]. Hence, investigating how Foxi1 regulates diverse processes ranging from germ layer specification to cell type formation in the *Xenopus* mucociliary epidermis could reveal insights relevant to our understanding of vertebrate development as well as human diseases.

In this work, we elucidate novel concentration-dependent Foxi1 transcriptional and epigenetic functions in mucociliary development and ISC specification. In blastula and early gastrula stages, Foxi1 is expressed at lower levels and required to retain epidermal identity in a population of multipotent mucociliary progenitors (MPPs) residing in the deep layer of the prospective epidermis. MPPs are regulated by Foxi1 through transcriptional and epigenetic means, and are required for mucociliary cell type generation as well as Notch-mediated patterning. At high levels, Foxi1 then induces ISC specification in later gastrula and neurula stages. We further demonstrate that high Foxi1 expression in ISCs is achieved through auto-regulation, and that ISC differentiation is guided by additional transcription factors, Ubp1 and Dmrt2, which regulate α- and β-ISC subtype development.

## Results

### Foxi1 is expressed in multipotent mucociliary progenitors (MPPs)

**Fig 1:**
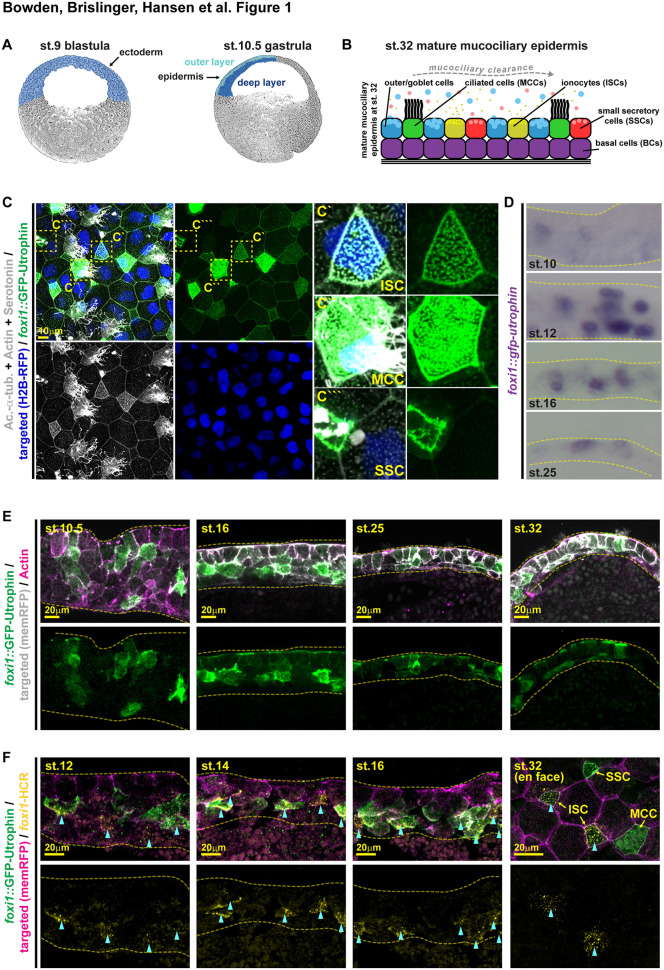
Foxi1 is transiently expressed in multipotent mucociliary progenitors (MPPs). **(A)** Schematic representations of a st. 9 blastula embryo (with prospective ectoderm expressing *foxi2* and *sox3* indicated in blue), of a st. 10.5 gastrula embryo (with outer layer epidermal cells indicated in light blue and deep layer cells indicated in dark blue), and **(B)** of the mature (st. 32) mucociliary epidermis with basal cells (BCs) in purple, outer/goblet cells in blue, ionocytes (ISCs) in yellow, multiciliated cells (MCCs) in green, and small secretory cells (SSCs) in red. **(C–F)** Analysis of *foxi1::gfp-utrophin* reporter (green) injected embryos by IF **(C–F)** and WMISH against *gfp* **(D)**. **(C)** En face image, IF for Acetylated-α-tubulin (Ac.-α-tub., cilia, gray), F-actin (Actin, cell borders and morphology, gray), and serotonin in large vesicular granules (SSCs, gray) at st. 32. Targeted cells were identified by nuclear RFP expression (H2B-RFP, blue). Magnifications of intercalating GFP(+) cell types are shown in insets. Location of insets is indicated by dashed yellow boxes in left panels. *n* = 12 embryos. **(D)** Sections of epidermal locations from embryos depicted in S2C Fig show *gfp*-expressing cells in the epidermis at key stages of mucociliary development (st. 10, 16, 25, 32). st. 10 *n* = 19; st. 12 *n* = 16; st. 16 *n* = 14; st. 25 *n* = 14 embryos. **(E)** IF for *foxi1::gfp-utrophin* reporter (green) and F-actin (Actin, cell borders and morphology, magenta) at st. 10.5–32 on hemisected embryos. Apical up, basal down. Targeted cells were identified by membrane RFP expression (memRFP, gray). Additional stages shown in S3A Fig. st. 10.5 *n* = 4; st. 16 *n* = 6; st. 25 *n* = 4; st. 32 *n* = 5 embryos. **(F)** HCR staining of endogenous *foxi1* transcripts (yellow) and IF for *foxi1::gfp-utrophin* reporter (green) at st. 12–16 on hemisected embryos and at st. 32 with en face view of the epithelium. Targeted cells were identified by membrane RFP expression (memRFP, magenta). Blue arrowheads indicate *foxi1*-HCR (+) cells. Stages st. 12 *n* = 5; st. 14 *n* = 4; st. 16 *n* = 3; st. 32 *n* = 4 embryos. Dashed lines demark the epidermis; apical up and basal down, in (D–F).

Previous work in *Xenopus* has demonstrated that the maternally deposited transcription factor Foxi2 directly binds the *foxi1* promoter and activates its expression during zygotic genome activation (ZGA) [6]. Recent work further demonstrated that Foxi2 cooperates with maternally deposited Sox3 in the early priming of genes for their activation during ZGA throughout the ectoderm [21], and that *foxi1* transcription is activated in the deep layer of the epidermal ectoderm (Fig 1A). The deep ectodermal cell layer, also called sensorial layer, gives rise to precursors and has been predominantly investigated in the context of neural and placodal development [22–25]. In the epidermis, the deep layer generates intercalating mucociliary cell types including ISCs, MCCs, and small secretory cells (SSCs) as well as basal cells (BCs) that serve as stem cells of the epidermis (Fig 1B) [8,9,24,26]. However, how this population of cells is established and regulated during mucociliary development is not well understood.

To re-evaluate Foxi1 functions in epidermis development, we first analyzed *foxi1* expression by whole-mount in situ hybridization (WMISH). Shortly after ZGA, in early blastula/gastrula (st. 9/10) stages, *foxi1* was expressed at low levels in patches of the prospective ectoderm, which started to resolve at st. 12 with individual cells strongly increasing *foxi1* expression by st. 16, resulting in a salt-and-pepper pattern of individual cells by st. 32, representing individual ISCs (S1A Fig). Hence, *foxi1* seemed to be transiently expressed in more epidermal cells than just in developing ISCs. We wondered if *foxi1* might be initially expressed at low levels in epidermal mucociliary multipotent progenitors (MPPs) in the deep epidermal layer. To test this, we generated a fluorescent reporter using the previously characterized *foxi1* promoter fragment harboring Foxi2 binding sites [6] driving the expression of GFP fused to the actin-binding protein Utrophin for stable long-term labeling (*foxi1::gfp-utrophin*) (S1B and S1C Fig). We injected embryos with *foxi1::gfp-utrophin* DNA and analyzed reporter activity at st. 32 by immunofluorescence (IF) and confocal microscopy. GFP signal was detected in ISCs, MCCs, and SSCs, and even some goblet cells expressed GFP at low levels (Fig 1C). In contrast, a Mcidas/Foxj1-regulated promoter construct driving mScarletI fluorescence (α-*tub::mscarletI*) was expressed predominantly in MCCs (S2A and S2B Fig), as previously described [27,28].

Next, we confirmed that temporal reporter expression dynamics resemble endogenous *foxi1* expression during epidermis development using WMISH (Figs 1D and S2C) and GFP expression by IF (Figs 1E and S3A). While plasmid injections lead to mosaic expression in the embryo, reporter-driven *gfp* transcripts were detected at st. 9–32, starting with non-epithelial low-level expression at st. 9/10, which increased by st. 12/16 in deep and superficial layer cells, and at st. 32 expression was found predominantly in epithelial layer cells (Figs 1D and S2C). GFP-fluorescent cells were detected from st. 10 onwards, predominantly in deep-layer cells, but also in some cells of the outer epithelial layer (Figs 1E and S3A). During st. 12–16, an increasing number of cells became GFP(+), including intercalating differentiating cells (Fig 1E and S3A). During st. 20–32, the number of GFP(+) cells decreased and fluorescent cells were progressively confined to the epithelial outer cell layer—however, basal-positioned GFP(+) cells were detected even at st. 32 suggesting that MPPs not differentiating into intercalating cell types could become BCs (Figs 1E and S3A). To further validate reporter specificity, we stained *foxi1::gfp-utrophin* injected embryos by fluorescent hybridization chain reaction (HCR) for endogenous *foxi1* transcripts. During cell fate specification stages (st. 12–16), most GFP(+) cells were also stained by *foxi1*-HCR, however, at st. 32, when the mucociliary epidermis is mature, only GFP(+) ISCs were still co-stained by *foxi1*-HCR in the epithelial layer, but not MCCs, SSCs, or goblet cells (Fig 1F).

Together, these data support the conclusion that *foxi1* is initially expressed in a distinct population of MPPs during mucociliary epidermis development, and that *foxi1* is turned off in MCCs and SSCs during cell fate specification, while Foxi1 activity is maintained in ISCs.

### Foxi1 regulates genome accessibility of mucociliary genes in the epidermis

It was proposed that Foxi2 and Sox3 initially regulate broad epigenetic accessibility and gene expression in the ectoderm at ZGA, but that zygotic-expressed factors would be required to maintain accessibility and to drive gene expression in the epidermis during subsequent development [21]. Besides its effects counteracting mesendoderm induction through transcriptional activation of ectodermal genes in early *Xenopus* embryos [7], Foxi1 has been shown to remain bound to condensed chromatin during mitosis, to remodel nucleosome structure, and to alter the transcriptional ground state of cells in zebrafish embryos [29]. This suggested that zygotic expression of *foxi1* could regulate both epigenetic state and transcriptional activity in epidermal MPPs.

**Fig 2:**
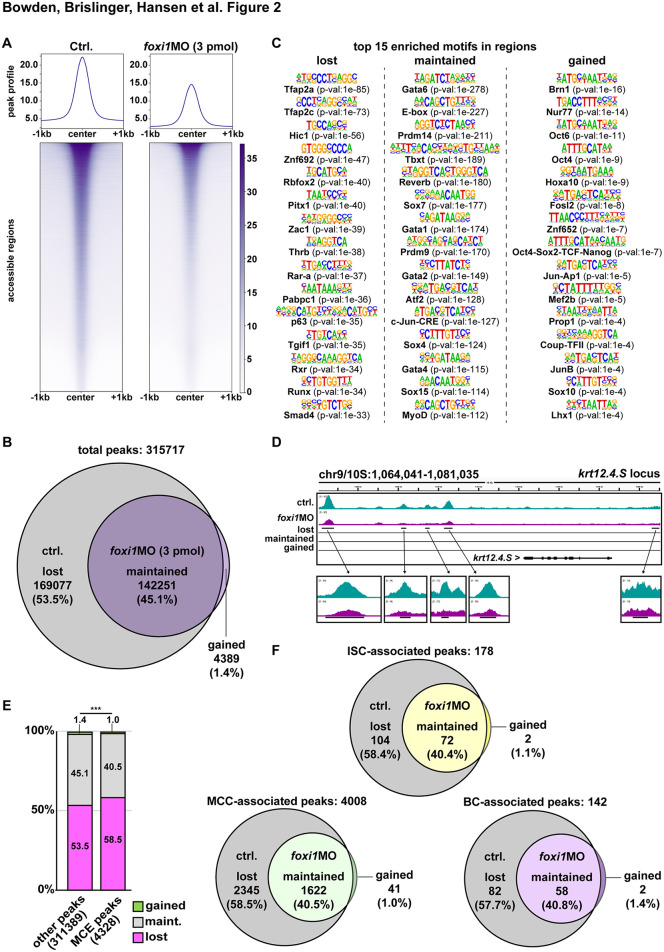
Foxi1 regulates chromatin state and mucociliary epidermal competence. **(A)** Profiles of ATAC-Seq normalized accessibility around peak center ±1 kb in controls (ctrl.) and foxi1 morphant (*foxi1* MO, 3 pmol) organoids. *n* = 2 organoids per condition and replicate. 3 replicates. (**B)** Venn diagram of peaks present in uninjected organoids (gray) and *foxi1* MO-injected organoids (purple). (**C)** Top 15 transcription factor binding motifs predicted by HOMER in sets of peaks with lost, maintained or gained accessibility after *foxi1* MO. **(D)** Distribution of accessible regions around epidermal *krt12.4.S.* Lost, maintained, and gained tracks as generated by MACS2 bdgdiff analysis and visualized in IGV. Turquoise track = control (ctrl.) and purple track = morphant (*foxi1* MO). **(E)** Enrichment of lost peaks in proximity of genes associated with mucociliary cell type development (MCE peaks). Green = gained; gray = maintained (maint.); magenta = lost. Chi^2^ test: *p* < 0.001 = ***. **(F)** Venn diagrams of peaks present in uninjected organoids (gray) and *foxi1* MO-injected organoids (colored). ISC-associated peaks = yellow; MCC-associated peaks = green; BS-associated peaks = magenta. Data used for panels (A) and (E): S1 and S2 Data. Motif enrichment files: S3 and S4 Data.

To test if Foxi1 affects chromatin state and genomic accessibility in *Xenopus* epidermal development, we performed assays for transposase-accessible chromatin with sequencing (ATAC-seq) after morpholino oligonucleotide (MO; 3 pmol) knockdown of *foxi1* (S3B Fig). For these experiments, we used “animal cap”-derived organoids to specifically investigate chromatin state in pure epidermis tissue [30,31]. This analysis revealed a dramatic reduction in accessible chromatin regions (peaks) after loss of Foxi1 (control: 311,328, *foxi1* MO: 146,640) (Fig 2A and 2B). In Foxi1-depleted organoids, 53.5% of accessible regions (169,077 peaks) were lost, 45.1% were maintained (142,251 peaks), and 1.4% were gained (4,389 peaks) (Fig 2B). Next, we investigated which transcription factor binding motifs were enriched in regions lost, maintained or gained after *foxi1* MO. We found that motifs for factors with known functions in *Xenopus* ectodermal development were enriched in regions that lost accessibility after *foxi1* knockdown (Fig 2C). These include Tfap2a and Tfap2c, Hic1, Rbfox2, Zac1 that regulate neural (crest) formation as well as Tp63, which regulates epidermal basal stem cells, and Pitx1, which is required for cement gland formation [32–37]. In contrast, regions that remained open were enriched for mesendodermal transcription factor motifs (e.g., Gata6, Tbxt, MyoD), and regions that gained in accessibility were enriched in pluripotency factors (e.g., Brn1, Oct4) (Fig 2C) [38–42]. Interestingly, Fox-factor motifs similar to the core-Foxi1 motif (TGTTT) were enriched more among the lost fraction of peaks (11.58% Fox/Ebox, 17.62% FoxO, 0.6% FoxA) than in the maintained (9.78% FoxO) or gained (no Fox motif enrichment) peak fractions. Together, these data support a function for Foxi1 in retaining accessible chromatin state during epidermal development in *Xenopus*.

Next, we wondered how loss of Foxi1 affects chromatin accessibility in regions harboring important genes for mucociliary epidermis development. First, we inspected a region around the *krt12.4* (epidermal keratin; marker for epidermal identity in *Xenopus*) [5] gene on chromosome 9/10.S, which revealed strongly reduced accessibility and indicated a loss of epidermal competence (Fig 2D) [7,43]. We further inspected genomic loci containing genes associated with mucociliary development (*dll1.L*), ISCs (*ubp1.L* and *dmrt2.S*), MCCs (*foxj1.L*), and BCs (*tp63.L*) [3,24,44,45]. In all cases, we found reduced accessibility (S3C Fig). To investigate if the loss of Foxi1 particularly affected mucociliary genes as compared to other genes in the ectoderm, we investigated loci associated with published core-ISC, -MCC, and -BC genes [44,45]. Strikingly, loss of accessibility in mucociliary gene-associated (MCE) peaks was significantly higher than in the rest of the genome (Fig 2E), increasing from 53.7% lost non-MCE peaks to 57.7% lost BC-associated peaks, to 58.4% lost ISC-associated peaks, and to 58.5% lost MCC-associated peaks (Fig 2F), and Fox-factor motifs were enriched in the MCE fraction of peaks that lost accessibility after *foxi1* MO (S4 Data).

In conclusion, Foxi1 regulates chromatin accessibility required for ectoderm and particularly for mucociliary cell type development. This provides an additional rational how Foxi1 could regulate mucociliary MPPs in early *Xenopus* epidermal development.

### Foxi1 acts in a concentration-dependent manner

**Fig 3:**
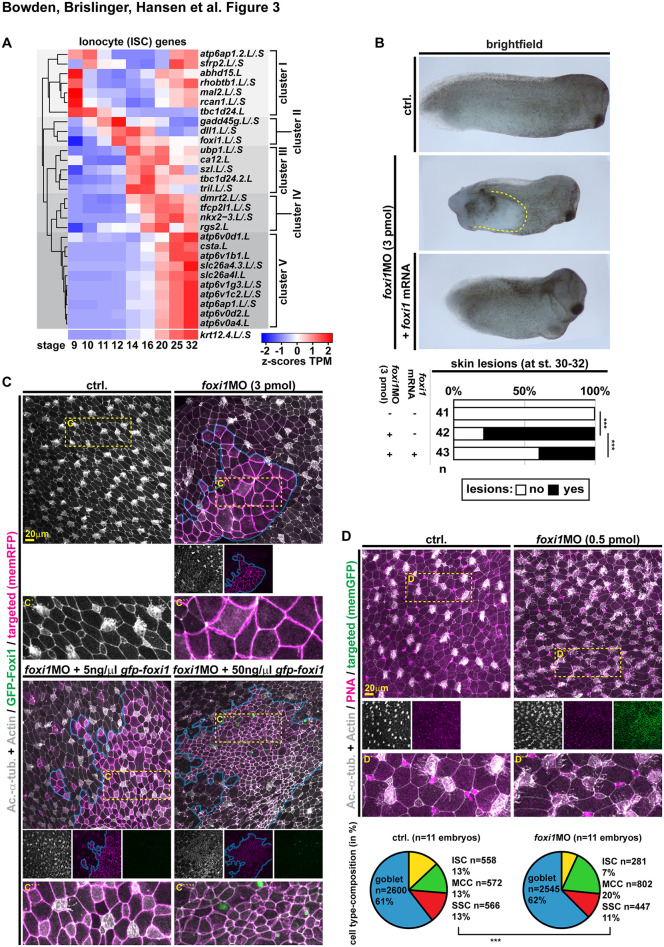
Foxi1 acts in a concentration-dependent manner. **(A)** Temporal expression analysis of core ISC genes. Clustered heatmap of line-normalized z-scores of TPMs (transcripts per million reads) derived from mRNA-seq of *Xenopus* mucociliary organoids over the course of development (st. 9–32). *krt12.4.L/.S* expression plot is shown below, but not included in clustering analysis. **(B)** Brightfield images and quantification of st. 30–32 embryos. Uninjected controls (ctrl.), *foxi1* morphants (*foxi1* MO; 3 pmol) and morphants co-injected with *foxi1* mRNA (50 ng/μl) are depicted anterior to the right, dorsal up. Skin lesions (dashed yellow outline) were quantified. Chi^2^ test: *p* < 0.001 = ***. **(C,D)** Immunofluorescence confocal micrographs (IF) from control (ctrl.) and *foxi1* morphants (*foxi1* MO; 3 or 0.5 pmol) at st. 32 stained for Acetylated-α-tubulin (Ac.-α-tub., cilia, gray), F-actin (Actin, cell borders and morphology, gray). **(C)** Targeted cells were identified by membrane RFP expression (memRFP, magenta) and are outlined in blue. Strongly targeted areas targeted by 3 pmol of *foxi1* MO fail to generate intercalating cell types, while co-injection of 5 and 50 ng/μl *gfp-foxi1* (green) rescues intercalated cell formation (identified by cilia staining or small apical surface of cells). Location of insets is indicated by dashed yellow box in upper panels. Ctrl. *n* = 9; *foxi1* MO *n* = 13; *foxi1* MO + 5 ng/μl = 7; *foxi1* MO + 50 ng/μl = 8. (**D**) To analyze cell type composition after low concentration of *foxi1* MO (0.5 pmol), mucus (PNA, magenta) was stained to reveal secretory cell types. Targeted cells were identified by membrane GFP expression (memGFP, green). Location of insets is indicated by dashed yellow box in upper panels. Quantification of cell type composition is depicted as pie-charts, goblet cells (blue), ISCs (yellow), MCCs (green), and SSCs (red). *n* embryos (above chart) and *n* quantified cells (in/left of chart). Chi^2^ test: *p* < 0.001 = ***. Data used for panels (A), (B), and (D): S1 Data.

Epidermal *foxi1* expression dynamics suggested that Foxi1 might act in a concentration-dependent manner: During early stages (st. 9–11), when MPPs are formed, *foxi1* expression is relatively low, while at stages when ISCs are specified and begin to differentiate (st. 12–16), *foxi1* expression dramatically increases in a subset of cells (S1A Fig). To validate this observation using a quantitative approach and additional ISC markers, we used a previously defined core-ISC gene set in *Xenopus laevis* [44] and investigated gene expression specifically in epidermal tissue using bulk RNA-sequencing (RNA-seq) on mucociliary organoids [30,45,46]. Z-scores of normalized counts (TPM) of ISC transcripts were clustered to reveal dynamic co-expression (Fig 3A). Five clusters clearly separated along developmental time, with cluster I being the only set of genes displaying strong expression during very early and late developmental stages, but not during cell fate specification stages (st. 10–16). Cluster II contained *foxi1*, the Notch ligand Delta-like 1 (*dll1*) and the cell cycle regulator *gadd45g*. Cluster III contained the pH-regulator Carbonic Anhydrase 12 (*ca12*; a pH regulator expressed in ISCs; [47]) and the transcription factor *ubp1*, which was shown to induce ectopic ISCs upon overexpression in the epidermis [3]. Cluster IV contained multiple transcription factors, including *tfcp2l1*, required for ISC formation in the mouse kidney [48]. Cluster V was dominated by solute carrier (e.g., *slc26a4*) and pH-regulator (*atp6*-subunits) expression during later differentiation of ISCs (st. 20–32), similar to overall differentiation of the epidermis marked by *krt12.4* expression. These data confirmed that *foxi1* expression levels were initially low (st. 9–11), and that they peaked at st. 12–14, shortly before the first functional ISC genes (e.g., *ubp1* and *ca12*) were expressed at st. 14–16 (Fig 3A) [3,47].

To test the hypothesis that high Foxi1 levels are required for ISC specification while lower Foxi1 levels are sufficient to retain epidermal identity, we first injected high *foxi1* MO doses (3 pmol; as used for the ATAC-seq experiments) targeted to the developing epidermis. This treatment induced delamination of cells without inducing apoptosis (TUNEL staining; [27]) in st. 9/10 embryos (S4A and S4B Fig) and frequent formation of skin lesions at st. 32 (Fig 3B) as previously described [5]. Analysis of neurula stage embryos (st. 19) injected with 3 pmol *foxi1* MO further revealed extensive gastrulation defects [49] on the injected side of morphants (S4C and S4D Fig). This was in line with the observed delamination of a subset of Foxi1-depleted cells (S4A Fig), which affects epithelial integrity and likely prevents epiboly of cells during gastrulation, when ectodermal tissues are significantly strained [50]. Hence, loss of MPPs in combination with gastrulation defects causes skin lesions observed in later tadpole stages. The formation of skin lesions at st. 32, as well as gastrulation defects could be partially rescued by co-injection of *foxi1* mRNA (using 10 or 50 ng/μl), confirming MO specificity (Figs 3B and S4C).

Next, we performed IF on targeted regions of the epidermis at st. 32 to investigate mucociliary cell type formation and epidermal morphology. Cells receiving the highest doses of *foxi1* MO, marked by higher levels of co-injected lineage tracer (membrane RFP; outlined in blue), did not form intercalating cell types including MCCs (revealed by acetylated-α-Tubulin staining in cilia) (Figs 3C and S4E), as previously described [5]. Next, we co-injected *foxi1* morphants with different concentrations of mRNA encoding GFP-tagged *foxi1* (*gfp-foxi1*) and analyzed rescue effects in the epidermis by IF. Low levels (5–15 ng/μl) of *gfp-foxi1* improved epithelial morphology and lead to the re-appearance of intercalating cells, including MCCs as well as cells with ambiguous morphology (Fig 3C and S4E). High levels (50 ng/μl) of *gfp-foxi1* lead to a strong overproduction of intercalating cells with ISC-like morphology without induction of MCCs (Figs 3C and S4E). This suggested that low Foxi1 levels rescue MPPs (able to generate different intercalating cell types), while high Foxi1 levels induce ISCs.

To further explore this question and to relate epidermal to overall morphological defects in *foxi1* morphants, we analyzed the expression of the ISC marker *ubp1* and the MCC marker *mcidas* at st. 19 by WMISH in the same embryos used for the assessment of gastrulation defects (S4C, S4D, and S4F Fig). In *foxi1* morphants, *ubp1* and *mcidas* were both detected, independent of the severity of gastrulation defects (S4F Fig). In morphologically normal embryos and embryos with mild gastrulation defects, expression of both markers was reduced. In specimens with severe gastrulation defects, large areas devoid of marker expression were observed in most cases (indicated by red arrows in S4F Fig). This argued for a loss of competence to generate intercalating cells, which was most prevalent in areas adjacent to epidermal lesions and embryos showing severe gastrulation defects. Co-injection of low (10 ng/μl) *gfp-foxi1* mRNA concentrations rescued partially the expression of ISC and MCC markers in embryos with severe gastrulation defects, and in a subset of cases, *mcidas* expression was increased beyond wt levels (indicated by yellow arrows in S4F Fig). Co-injection of high (50 ng/μl) *gfp-foxi1* mRNA concentrations also rescued ISC and MCC markers in embryos with severe gastrulation defects, however, while *ubp1* expression further increased (indicated by yellow arrows), we started to observe gaps within the *mcidas* domain that lacked expression (indicated by red arrows in S4F Fig). Despite the fact that a reliable assessment and quantification of cell type composition by IF or WMISH was limited by the severe morphological changes induced in morphants, these results suggested that low Foxi1 levels rescued epidermal MPPs able to generate ISCs and MCCs, while high Foxi1 levels induced supernumerary ISC formation instead. Therefore, we tested the concentration-dependent rescue effects by quantitative PCR (qPCR) at st. 10.5. We assessed a pan-epidermal marker (*krt12.4*) and a definitive-ISC marker (*ubp1*) on control and *foxi1* MO (3 pmol) injected samples as well as after co-injection of 5–50 ng/μl *gfp-foxi1* mRNA (S5A Fig). This revealed a significant reduction in *krt12.4* in morphants, which was partially rescued by 15 ng/μl, but not 50 ng/μl of *gfp-foxi1*, while *ubp1* was significantly over-induced by 50 ng/μl of *gfp-foxi1*. Furthermore, we validated that different protein levels were induced at relevant stages (st. 12) using different *gfp-foxi1* mRNA concentrations by western blot analysis (anti-GFP antibody) (S5B Fig and S1 Raw Images). While we could not assess endogenous Foxi1 protein level, collectively these data supported the hypothesis that lower levels of Foxi1 are sufficient to rescue epidermal identity and the formation of intercalating cell types from MPPs, while higher Foxi1 levels are required for ISC specification.

To confirm this, we injected low concentrations of *foxi1* MO (0.5 pmol) aiming at reducing only peak expression levels of Foxi1 without interfering with epidermis identity or MPPs, and analyzed cell type composition and morphology in the mature mucociliary epidermis at st. 32 by IF [30]. This mild *foxi1* knockdown specifically reduced ISC formation without affecting epidermal identity as evidenced by the formation of other intercalating cell types (Fig 3D): We observed little effects on secretory cells (SSCs and goblet cells) and while MCC ciliation was reduced as previously described in *X. tropicalis* [47], the overall number of MCCs was increased (Fig 3D). This indicated that low concentrations of *foxi1* MO reduced Foxi1 levels enough to inhibit ISC specification (leading to supernumerary MCC specification), but not strong enough to interfere with ectoderm specification and MPP development.

This raised the question how MPPs can achieve high *foxi1* expression levels required for specification of ISC fate. One potential mechanism for conferring robust cell fate decisions is positive auto-regulation, and Foxi1 could activate its own expression using core Foxi motifs previously identified in the *foxi1* promoter [6,51]. To test if Foxi1 is required for *foxi1* expression in MPPs and differentiating cells, we injected both blastomeres at 2-cell stage with *foxi1::gfp-utrophin* reporter (together with nuclear *H2B-rfp* mRNA) followed by a single injection of *foxi1* MO (3 pmol; together with membrane *mem-rfp* mRNA) into one balstomere at 4-cell stage (S5C Fig). Embryos were raised to st. 19, fixed, stained for F-actin, sectioned transversally, and imaged with a confocal microscope. This analysis revealed GFP-utrophin signal in reporter-only injected cells, while cells co-injected with *foxi1* MO showed strongly reduced reporter activity in the epidermis (S5D Fig). Furthermore, in areas where single and double targeted cells mixed, we observed reduced GFP-utrophin signal in MO-targeted cells (S5E Fig). These data indicated that Foxi1 is required to maintain *foxi1* reporter activity during cell fate specification in the mucociliary epidermis in a cell-autonomous manner.

Next, we deleted a validated Foxi2 binding region (*foxi1*Δ*Foxi2BR::gfp-utrophin*) (S1B and S1C Fig) and analyzed reporter activity comparatively to *foxi1::gfp-utrophin* at st. 12 by qPCR as well as by IF and confocal microscopy of the epidermis in vivo at st. 32, i.e., long after *foxi2* expression is terminated. The *foxi1*Δ*Foxi2BR::gfp-utrophin* construct showed decreased reporter activity (S6A and S6B Fig), suggesting that core Foxi motifs are also used by Foxi1 to maintain its expression through auto-regulation. Injection of *foxi1* mRNA also affected endogenous *foxi1* expression at st. 12 (but not to a significant degree), measured by qPCR for 3′UTR sequences not present in the synthetic *foxi1* mRNA used for manipulations (S6B Fig). While *foxi1.L* was upregulated, *foxi1.S* was downregulated, suggesting potential feedback effects in the developing epidermis. To verify that Foxi1 can also activate its own promoter without contributions from Foxi2, we injected *foxi1::gfp-utrophin* vegetally to target the prospective mesendoderm, which lacks maternally deposited *foxi2* [6]. Analysis of reporter-only injected cells (marked by membrane RFP) in hemisected embryos at st. 11 showed no reporter activity in endodermal cells, while co-injection of *foxi1* mRNA led to ectopic activation of the reporter (S6C Fig).

**Fig 4:**
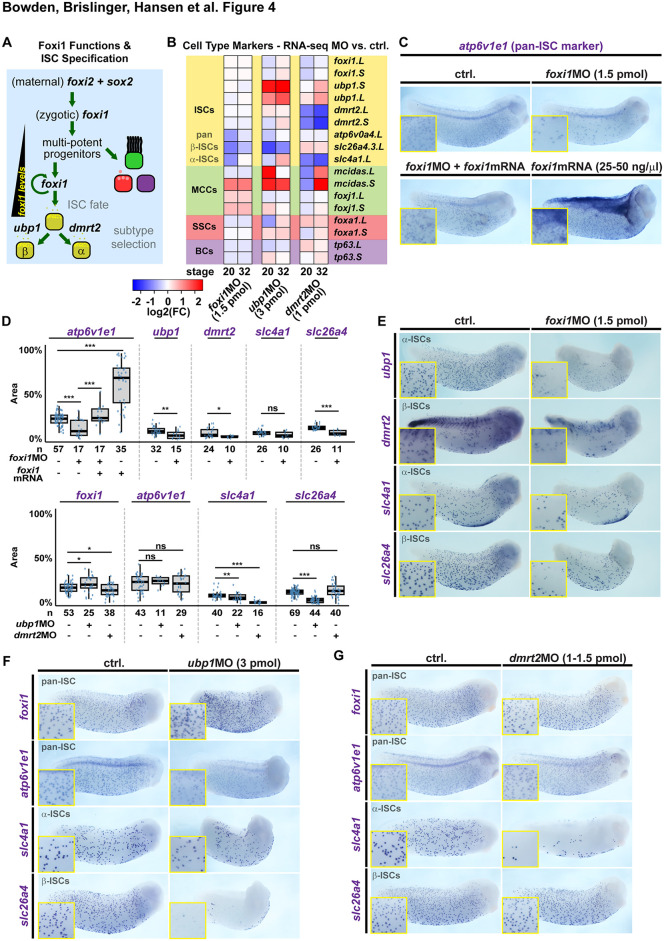
Foxi1, Ubp1, and Dmrt2 differentially regulate ionocyte development. **(A)** Schematic representation of MPP and ISC specification in relation to transcription factor expression. **(B)** Gene expression analysis of mucociliary cell fate regulators after knockdown of Foxi1 (*foxi1* MO; 1.5 pmol), Ubp1 (*ubp1* MO; 3 pmol), and Dmrt2 (*dmrt2* MO; 1 pmol). Heatmap of log2-fold changes relative to control samples derived from DEseq2 analysis of mRNA-seq on *Xenopus* mucociliary organoids during ISC differentiation (st. 20) and in mature epidermis stage (st. 32). **(C–G)** Knockdown of ISC transcription factors (*foxi1* MO, 1.5 pmol; *ubp1* MO, 3 pmol; *dmrt2* MO, 1 pmol) and analysis of effects by WMISH at st. 29–32 against *atp6v1e1* and *foxi1* (pan-ISC markers), *ubp1* and *slc26a4/pendrin* (β-ISC markets), and *dmrt2* and *slc4a1/ae1* (α-ISC markers). Representative images, embryos depicted anterior to the right, dorsal up, insets show magnification of epidermis in **(C,E,F,G)** and quantification of results in **(D)**. *n* = number of embryos analyzed per condition. **(C)** Co-injection of 25–50 ng/μl of *foxi1* mRNA rescues expression of pan-ISC marker *atp6v1e1* in *foxi1* morphants (1.5 pmol) and *foxi1* overexpression induces broad *atp6v1e1* expression in the epidermis. In **(D)**, marker-positive area within a standardized region of the embryo was analyzed and is depicted as % of analyzed area. Wilcoxon Rank Sum test: *p* > 0.05 = ns; *p* < 0.05 = *; *p* < 0.01 = **; *p* < 0.001 = ***. **(D,E)** Foxi1 knockdown (*foxi1* MO, 1.5 pmol) leads to loss of marker expression in both ISC subtypes. **(D,F)** Ubp1 knockdown (*ubp1* MO, 3 pmol) leads to loss of β-ISC marker. **(D,G)** Dmrt2 knockdown (*dmrt2* MO, 1 pmol) leads to loss of α-ISC marker. Data used for panels (B) and (D): S1 Data.

Together these data supported the conclusion that low levels of Foxi1 are sufficient for ectoderm identity and MPPs, that high Foxi1 levels are required for ISC specification, and that the *foxi1* expression increase in ISCs is achieved by positive auto-regulation (Fig 4A).

### Specification and differentiation of ISCs is a multistep process

Besides concentration-dependent effects of transcription factors, an important route to modulate transcription factor functions during development is the cooperation with other transcription factors [52]. Therefore, we next investigated how other transcription factors contained in the core-ISC gene set regulate ISC specification (Fig 3A). Among the first definitive ISC genes expressed during mucociliary epidermis development were the transcription factors Ubp1 (Cluster III) and Dmrt2 (Cluster IV). Two ISC subtypes exist: α-ISCs that differentially express AE1 (encoded by *slc4a1*) and β-ISCs that express Pendrin (encoded by *slc26a4*) [3]. Additionally, both ISC subtypes express vH+-ATPase (encoded by *atp6*-subunits) [3,53]. Ubp1 was previously shown to induce β-ISCs in *Xenopus*, and Dmrt2 was recently shown to be required for α-ISC formation in the mouse kidney [3,54].

We used single-cell RNA-seq (scRNA-seq) data from at late *X. laevis* tail stages containing mature ISCs [55] to investigate α- and β-ISC gene profiles. These data revealed high levels of *foxi1* and *atp6*-subunit expression in both ISC subtypes, while *ubp1* was enriched and *slc26a4* was specifically expressed in β-ISCs (S6D Fig). In contrast, *slc4a1* as well as *dmrt2* were expressed only in α-ISCs (S6D Fig). To reveal ISC-subtype specific functions, we tested how *foxi1*, *ubp1*, and *dmrt2* contribute to ISC-subtype formation by RNA-seq on mucociliary organoids during differentiation (st. 20) and when the epidermis is mature (st. 32) (Fig 4B). Knockdown of Foxi1 (1.5 pmol *foxi1* MO) should reduce expression of all ISC genes, while knockdown of Ubp1 (3 pmol *ubp1* MO) and Dmrt2 (1 pmol *dmrt2* MO) should yield differential effects on the subtype-specific ISC markers (*slc4a1* – α-ISCs; *slc26a4* – β-ISCs) (S3B Fig). RNA-seq on *foxi1* morphant organoids revealed a reduction of ISC differentiation markers *atp6*, *slc26a4*, and *slc4a1* (Fig 4B), and the expression of core-ISC genes was reduced across all clusters (S7A Fig). Furthermore, the MCC genes *mcidas* and *foxj1* were upregulated, in line with our finding that MCCs are over-produce when ISC specification was blocked (Fig 3D). In contrast to *foxi1* MO injections, knockdown of *ubp1* caused strong downregulation of *slc26a4*, while *atp6* was only transiently reduced and *slc4a1* was elevated at st. 32 (Fig 4B). A reduction of ISC-gene expression was predominantly found in cluster I and V genes, while genes in cluster III were upregulated (S7B Fig). This indicated a specific loss of β-ISCs as well as a change in differentiation dynamics. Knockdown of *dmrt2* caused strong downregulation of *slc4a1*, while *atp6* remained unchanged and *slc26a4* was elevated (Fig 4B). Across core-ISC genes, *dmrt2* MO only led to a strong downregulation of *dmrt2* expression, while most genes across all clusters were only transiently reduced (S7C Fig). This indicated a specific loss of α-ISCs. Furthermore, differentiation dynamics appeared to be dysregulated after *dmrt2* MO, and similar to *foxi1* as well as *ubp1* MOs, an upregulation of *mcidas* was observed (Figs 4B and S7C).

To validate the findings from manipulated organoids, we knocked down each factor and analyzed ISC marker expression in the mature epidermis at st. 30–32 by WMISH and semi-automated image analysis. *foxi1* (MO, 1.5 pmol) knockdown strongly reduced the pan-ISC marker *atp6v1e1* expression, which could be rescued by co-injection of *foxi1* mRNA (Fig 4C and 4D). Furthermore, 1.5 pmol of *foxi1* MO reduced *ubp1*, *dmrt2*, as well as *slc26a4* expression, and to a minor degree *slc4a1*, in line with the RNA-seq data (Fig 4B, 4D, and 4E), indicating a loss of both ISC subtypes. As automated image analysis of complex in vivo samples can strongly vary depending on the used cutoff parameters, we further modified the area limit parameters (cf. Materials and methods) and compared the results between automated analyses. The results for strongly affected markers *atp6v1e1* and *slc26a4* yielded the same statistical results (S5 and S6 Data). However, the statistical significant effect on *ubp1* expression was changed to no significant effect in the modified analysis, the non-significant effect on *slc4a1* became significant (*p* < 0.01, **), and the effect on *dmrt2* became more significant (from *p* < 0.05, * to *p* < 0.01, **) (S5 Data). While the use of alternative parameters overall supported the conclusions as well as the use of area measurement as proxy for cell count (S5 Data), it also highlighted the limitations of automated analysis approaches.

In contrast to *foxi1* MO, knockdown of *ubp1* strongly reduced expression of the β-ISC marker *slc26a4*, increased *foxi1*, and mildly reduced the α-ISC marker *slc4a1* (Fig 4D and 4F). Conversely, *dmrt2* loss led to a strong inhibition of α-ISC-specific *slc4a1* and a mild reduction in *foxi1*, without affecting *slc26a4* (Fig 4D and 4G). Importantly, *ubp1* and *dmrt2* MOs had only minor effects on the pan-ISC marker *atp6v1e1*, mostly in the form of reduced expression in some cells without significant reduction in the number of expressing cells (Fig 4D, 4F, and 4G). Validation of these automated quantification results using modified parameters further revealed changed significance for the effects of *ubp1* MO and *dmrt2* MO on *foxi1* expression as well as in the case of *dmrt2* MO effects on *atp6v1e1* and *ubp1* MO effects on *slc4a1* (S5 Data). Furthermore, MO-specific effects were confirmed in rescue experiments, and ISC-specific mRNA effects were confirmed by overexpression of *ubp1* and *dmrt2* causing supernumerary induction of ISC subtypes (S7D and S7E Fig).

Taken together, these data support the conclusion that Foxi1 determines ISC fate commitment, while Ubp1 and Dmrt2 cooperate with Foxi1 during ISC-subtype differentiation in a multi-step process of ISC development (Fig 4A).

### MPPs and ISC transcription factors regulate Notch signaling during cell fate specification

Previous studies indicated that inhibition of Foxi1 at a level that prevents specification of ISCs but not MCCs negatively affected normal MCC ciliation [47]. In line with this observation, our data indicated that while MCC numbers were increased after *foxi1* MO (0.5 pmol), ciliation was reduced in many MCCs (inset in Fig 3D). Previously, we have shown that elevated Notch levels presented to MCCs after fate commitment interfered with normal ciliation and differentiation [27]. Hence, we hypothesized that a dysregulation of Notch signaling could cause the MCC ciliation phenotype. Notch signaling is required for mucociliary cell fate decision across mucociliary epithelia, and in the *Xenopus* epidermis, ISCs as well as MCCs were shown to be inhibited by Notch activity [3,24]. The Notch ligand *dll1* is expressed during epidermis development, and its expression was assigned to ISCs by Quigley and colleagues, similar to Foxi(+) cells in the zebrafish skin and mammalian kidney [4,44,56]. However, another study observed *dll1* expression overlapping with different cell markers during patterning stages in the *Xenopus* epidermis [57]. Our temporal expression analysis indicated very early *foxi1* and *dll1* expression in Cluster II, likely representing MPPs and early ISC differentiation stages (Fig 3A), in line with both published observations. Therefore, Foxi1 manipulations could dysregulate MPPs expressing *dll1*, and thereby alter Notch signaling during patterning in the epidermis.

To investigate how MPPs, Notch signaling and core-ISC genes are regulated in the mucociliary epidermis, we manipulated Notch and cell fates in organoids and investigated gene expression at st. 10.5, 16, 25, and 32. As previously described [3], increased Notch signaling (Notch intracellular domain (*nicd*) mRNA injections) inhibited core ISC gene expression, while inhibition of Notch signaling (injection of dominant-negative suppressor of hairless/RBPJ (*suh-dbm*) mRNA) promoted core ISC gene expression (S8A and S8B Fig). Inhibition of Notch signaling in combination with blocking MCCs (by co-injection of *dominant-negative mcidas* (*dn-mcidas*) mRNA [58]) further increased core ISC gene expression, reflecting stronger overproduction of ISCs. However, expression of *tfcp2l1*, *atp6v0d1.L*, and *csta.L* were reduced in these conditions suggesting that they might not be specifically expressed in ISCs (S8C Fig). Notch repression of *foxi1* by *nicd* was substantial but not statistically significant (S8A Fig), likely reflecting a Notch-independent regulation of Foxi1 in MPPs that is retained when the specification of intercalating cell types is inhibited.

**Fig 5:**
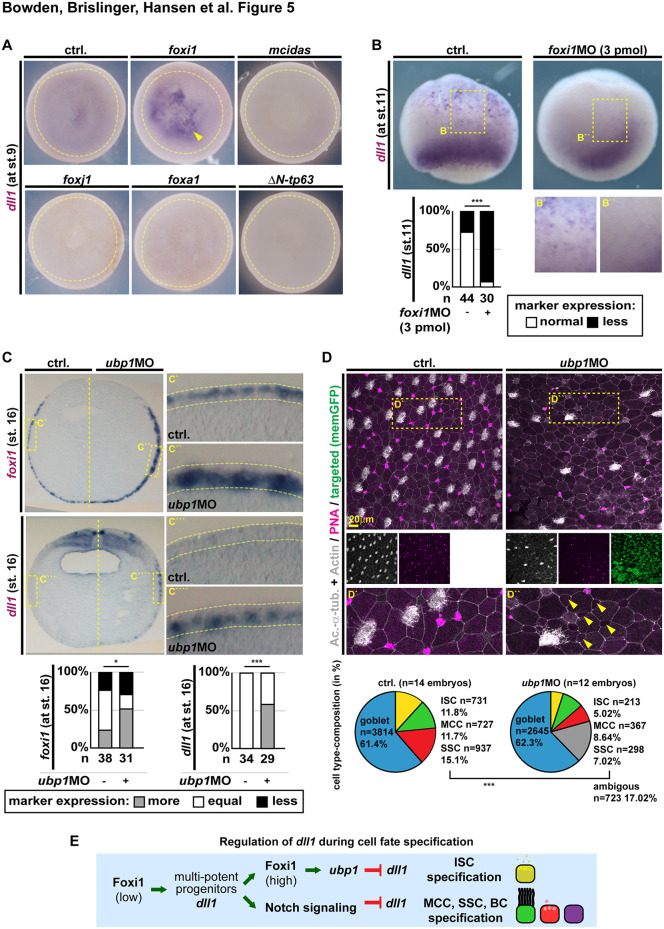
Foxi1 induces and Ubp1 terminates Notch ligand expression. **(A–C)** Manipulation of mucociliary cell fate transcription factors *foxi1* and *ubp1* (ISCs/MPPs), *mcidas* and *foxj1* (MCCs), *foxa1* (SSCs), and Δ*N-tp63* (BCs) and analysis of effects by WMISH at st. 9 (animal view, ectoderm outlined in yellow) **(A)**, st. 11 (ventro-lateral view, blastopore down, animal regions up) **(B)**, and st. 16 section of unilateral injected embryos; dorsal up, ventral down) **(C)** against *dll1* (Notch ligand) and *foxi1* (MPP/ISC marker). **(A)** Representative images of control (ctrl.) and manipulated embryos (animal views) after mRNA overexpression of transcription factors to test premature induction of *dll1*. Quantification of results and effects on *dlc* are shown in S8D Fig. Embryos were scored as induced or non-induced expression. Yellow arrowhead indicates induced expression. Yellow dashed line outlines prospective epidermis. **(B)** Representative images of control (ctrl.) and *foxi1* morphants (*foxi1* MO, 3 pmol) to test effects on *dll1* expression. Quantification of results is shown in lower panel. Insets show epidermal area magnification. Locations of insets are indicated by dashed yellow box in upper panels. Embryos were scored as normal or reduced (less) expression of *dll1*. Chi^2^ test: *p* < 0.001 = ***. **(C)** Representative images of sectioned embryos after unilateral knockdown of *ubp1* (*ubp1* MO, 3 pmol). Expression of markers was scored as more, less, or equal to uninjected control (ctrl.) side. Locations of insets are indicated by dashed yellow box in left panels. Epidermis is outlined in magnified images, apical up and basal down. Chi^2^ test: *p* < 0.05 = *; *p* < 0.001 = ***. **(D)** IF of control (ctrl.) and *ubp1* morphants (*ubp1* MO, 3 pmol) at st. 32 stained for Acetylated-α-tubulin (Ac.-α-tub., cilia, gray), F-actin (Actin, cell borders and morphology, gray), and mucus (PNA, magenta). Targeted cells were identified by membrane GFP expression (memGFP, green). Location of insets is indicated by dashed yellow box in upper panels. Ambiguous cells are indicated by yellow arrowheads. Quantification of cell type composition is depicted as pie-charts, goblet cells (blue), ISCs (yellow), MCCs (green), and SSCs (red). Ambiguous cells are depicted in gray. n embryos (above chart) and n quantified cells (in/left of chart). Chi^2^ test: *p* < 0.001 = ***, not including ambiguous cells. **(D)** Schematic summary of Dll1 regulation during mucociliary development. Data used for panels (B), (C), and (D): S1 Data.

Feedback regulation of *dll1* by Notch signaling was suggested in *Xenopus* epidermis development [24]. RNA-seq analysis of *dll1* expression after Notch manipulations confirmed that gain of Notch signaling suppresses *dll1*, while blocking Notch increases and prolongs *dll1* expression (S8A and S8B Fig). Interestingly, blocking MCC formation in Notch-inhibited organoids further increased and prolonged *dll1* expression, indicating that MCC fate specification inhibits *dll1* expression when MPPs adopt this cell fate (S8B and S8C Fig). To address if *dll1* (and *dlc*; Brislinger-Engelhardt and colleagues, 2023) expression is part of the differentiation program across multiple mucociliary cell types or specific to MPPs and early ISCs, we tested whether master transcription factors inducing cell fates of the mucociliary epidermis were able to induce Notch ligands prematurely. To induce the different cell types, we overexpressed *foxi1* for MPPs/ISCs, *mcidas* and *foxj1* for MCCs, *foxa1* for SSCs, and for BCs Δ*N-tp63* (Tp63 isoform that regulates mucociliary BCs [45]). Only *foxi1* robustly induced *dll1* and *dlc* (Figs 5A and S8D), and conversely, depletion of Foxi1 (3 pmol MO) prevented *dll1* expression during cell fate specification stages (Fig 5B). These results suggested that *dll1* is expressed in *foxi1*(+) MPPs and terminated by Notch signaling and cell fate induction of MCCs, SSCs, and BCs.

In differentiating and mature ISCs, which maintain *foxi1* expression, *dll1* expression should be also maintained. Nevertheless, RNA-seq data indicate that *dll1* expression is not maintained at high levels in the mature mucociliary epidermis (Fig 3A). This raised the question how *dll1* expression is terminated during ISC differentiation. To address that, we investigated published developmental *X. tropicalis* scRNA-seq data [59] and visualized enrichment for key mucociliary cell fate regulators and *dll1* across cell types and developmental stages. This confirmed that *dll1* is enriched early in the ISC lineage, and that *dll1* expression is lost once *ubp1* is expressed (S8E Fig). Furthermore, our RNA-seq data on manipulated organoids confirmed an upregulation of *dll1* when ISC differentiation was inhibited, and these effects were most pronounced after *ubp1* MO (S7A–S7C Fig). To validate that Ubp1 terminates *dll1* expression in embryos, we knocked down *ubp1* and analyzed embryos at the end of cell fate specification (st. 16) by WMISH. *foxi1* was maintained and *dll1* expression was prolonged on the *ubp1* MO injected side (Fig 5C). Analysis of cell type composition at st. 32 by IF in *ubp1* morphants further revealed reduced MCC and SSC numbers as well as appearance of intercalating cells with ambiguous morphology, likely representing incompletely differentiated ISCs (Fig 5D). These results revealed that manipulating *foxi1* and ISC differentiation leads to dysregulated Notch dynamics during mucociliary development. This explains why we and others have observed MCC ciliation defects after Foxi1 manipulations.

Together, these data suggest that Notch ligands are expressed by *foxi1*(+) MPPs during mucociliary patterning, and that Ubp1 terminates *dll1* expression in the ISC lineage. Furthermore, induction of MCC, SSC, and BCs also terminates Notch ligand expression in other differentiating mucociliary cell types. This provides a mechanism to regulate Notch signaling during mucociliary patterning that is tuned by both feedback-regulation (Notch) as well as fate commitment induced by mucociliary transcription factor networks (Fig 5E).

## Discussion

This work revealed that Foxi1 regulates multiple crucial steps during *Xenopus* mucociliary epidermis development through transcriptional and epigenetic mechanisms as well as its association with multipotent MPPs.

Previous studies have revealed that cells located in the deep layer of the prospective ectoderm contain progenitors of neural and epidermal tissues [24,60]. Additionally, single-cell transcriptomic studies confirmed that epidermal progenitors give rise to mature mucociliary cell types, ISCs, MCCs, SSCs, and BCs [26,59]. However, it remained largely enigmatic how such progenitors are generated, regulated, and separated from neural progenitor populations. Initially, *foxi1* expression is activated in the prospective ectoderm by maternally deposited *foxi2* and *sox2*, which regulate transcriptional activation as well as epigenetic accessibility of ectodermal genes [6,21]. Our data suggest that the resulting initial low-level expression of Foxi1 is required to maintain and regulate chromatin accessibility in MPPs when Foxi2 and Sox2 levels decrease after ZGA [21]. This is important to subsequently support the developmental functions of pro-ectodermal transcription factors (e.g., Tfap2a/c), mucociliary regulators (e.g., Tp63) as well as mediators of thyroid hormone, retinoic acid and TGFβ signaling (e.g., Thrb, Rar-a, Smad4) that were described to regulate ectodermal development [27,45,57,61,62]. Notch over-activation does not alter ectodermal identity in line with previous reports as baseline *foxi1* expression is Foxi2-/Sox2- but not Notch-dependent, whereas strong reduction in Foxi1 does lead to a failure of the epidermis to develop normally [6,21,24]. Hence, we propose that Foxi1 could maintain epidermis developmental potential in deep layer cells after the activities of maternal factors Foxi2 and Sox2 are reduced during gastrula stages [21] and that this is required to generate MPPs. Furthermore, our data support previous findings that loss of Foxi1 could facilitate acquisition of mesendodermal fates, as loci enriched for pro-mesendodermal transcription factors (e.g., Gata6, Tbxt, MyoD) remain accessible in the absence of Foxi1 [38–40]. It is attractive to speculate that this is possible, because multiple transcription factors enriched in the maintained fraction of peaks (e.g., Gata and Sox family members) are known factors with pioneer activity [63,64].

We further provide evidence that after regulating MPPs during early development, Foxi1 levels increase through auto-regulation, and that only high levels of Foxi1 induce ISC fate in cooperation with Ubp1 and Dmrt2. This is in line with the known role of Foxi1 as master transcription factor for ISCs across vertebrate tissues [1]. These findings support the conclusion that ISC fate, subtype selection and differentiation is a multi-step process. Importantly, we demonstrate that MPPs express Notch ligands required for mucociliary patterning during this process. This reconciles apparent discrepancies described in the literature, one suggesting that Notch ligands are expressed from ISCs (based on transcriptional induction) while the other suggesting Notch ligand expression can overlap with markers of ISCs, MCCs and SSCs (based on fluorescent in situ co-expression) during early mucociliary development [44,57]. Our data revealed that in the *Xenopus* epidermis, Ubp1 initially terminates Notch ligand expression in differentiating α- and β-ISCs. Subsequently, Ubp1 drives differentiation of β-ISCs, while Dmrt2 drives α-ISC differentiation. This highlights the importance of transcription factor cooperativity in cell fate decisions, in addition to Foxi1 concentration-dependent effects. Interestingly, Dmrt2 has recently been shown to be required for α-ISCs in the mouse kidney; however, not Ubp1, but Tfcp2l1 is employed in mammalian kidney β-ISCs [3,48,54]. Differential use of these grainyhead-like transcription factors (Ubp1 and Tfcp2l1) could explain why Dll1 expression is terminated in *Xenopus* epidermal ISCs, but remains active in mammalian kidney ISCs (also called INCs) [48,56]. Similar to Ubp1, our results suggest that fate acquisition of other mucociliary epidermal cell types terminates Dll1/Dlc ligand expression in MPPs. Together, this system provides a robust Notch feedback-regulated developmental program for mucociliary epidermis development [65], with Foxi1 as a central player that acts through transcriptional as well as epigenetic mechanisms, and that affects cell fate specification directly in ISCs as well as indirectly through dysregulating Notch ligand expression in MPPs. However, one limitation at this point is that we have not found a specific marker exclusively expressed in MPPs, which would be necessary to investigate MPP-specific behavior and gene expression further.

Collectively, our data argue that the different concentrations of Foxi1—low in MPPs and high during ISC generation—distinguish between MPP- and ISC-specific functions. (1) High MO doses lead to loss of epigenetic accessibility preventing epidermal mucociliary cell type development, while low MO doses lead to a loss of ISCs and, instead, MCCs were specified in excess. (2) Only high MO concentrations cause the delamination of ectodermal cells that was previously described [5], but without inducing cell death—at least during early developmental stages. The loss of epidermal cells from the forming epithelium not only leads to a lack of epidermal cell types, but also causes gastrulation defects. Ectodermal cells are “pulled” over the mesendoderm during gastrulation (evidenced by the stretch forces generated on the ectodermal tissue [50]), which is prohibited when cells delaminate and epithelial integrity is affected. The loss of MPPs in combination with gastrulation defects then gives rise to lesion in the epidermis during subsequent tailbud stages. (3) Rescue of the phenotypes induced by high doses of *foxi1* MO is concentration dependent, i.e., lower concentrations of exogenous Foxi1 can rescue mucociliary cell types, while higher concentrations induce supernumerary ISCs. This is line with the interpretation that high concentrations of Foxi1 are required for ISC specification. However, it is noteworthy that our study has clear limitations as we could not assess endogenous Foxi1 levels in wt and manipulated embryos directly due to lack of *Xenopus*-specific antibodies and non-reactivity of anti-Foxi1 antibodies commercially available. While all relevant morphant phenotypes could be rescued by mRNA co-injections in a concentration-dependent manner, MOs could have additional off targets, and exogenous mRNA injections might not fully recapitulate endogenous levels and expression dynamics. Hence, subsequent studies will have to address endogenous Foxi1 levels by *Xenopus*-specific antibodies, which will also be useful to investigate genomic regions bound by Foxi1 directly (e.g., by ChIP-seq). Such studies will also be able to address additional mechanistic aspects, e.g., whether core ISC genes such as *ubp1* and *dmrt2* are directly or indirectly regulated by Foxi1.

Finally, in ISCs of the mammalian airway mucociliary epithelium, Foxi1 also regulates the expression of *cystic fibrosis transmembrane conductance regulator*, and mutations in *FOXI1* and its transcriptional target solute carriers cause Pendred syndrome and hearing loss, male infertility, and distal renal tubular acidosis [2,13–15]. In contrast, Foxi1 overexpression is found in cancer subtypes, e.g., in chromophore renal cell carcinoma and in pulmonary large cell carcinoma, but how Foxi1 overactivation can lead to cancerous transformations remains unresolved [17–20]. Published scRNA-seq datasets [66] revealed that Foxi1 was highly enriched in pulmonary ISCs and Foxi2 was also enriched in mouse ISCs. Ubp1 and Dmrt2 were also enriched in ISCs, suggesting that they might contribute to α- and β-ISC formation in the airways as well. However, Ubp1 was not only enriched in ISCs, while Tfcp2l1 and Foxp1 were strongly enriched in ISCs, similar to murine kidney ISCs. This suggests a conserved function of the transcription factor network in ISCs across tissues, however, the relative contributions of downstream factors such as Dmrt2, Ubp1, Tfcp2l1, and Foxp1 seem to be adapted across tissues to optimize tissue functions (discussed here: [65]). Interestingly, overexpression of Foxi1 only induced ISCs (marked by ATP6 transcripts) at high levels, while low-level Foxi1-expressing cells did not express ISC markers [66], similar to *Xenopus* epidermis development. Furthermore, Foxi1 overexpression induced novel non-ISC cell states with unknown function, in line with our findings that Foxi1 is not only a master transcription factor for ISC specification. Hence, our finding that Foxi1 drives an MPP state during mucociliary epidermis development could serve as a starting point to better understand the role of Foxi1 in cancers and other alterations across mucociliary tissues.

## Materials and methods

### Animal experiments

Wild-type *X. laevis* were obtained from the European Xenopus Resource Centre (EXRC) at University of Portsmouth, School of Biological Sciences, UK, or Xenopus 1, USA. Frog maintenance and care was conducted according to standard procedures in the AquaCore facility, University Freiburg, Medical Center (RI_00544) and based on recommendations provided by the international Xenopus community resource centers NXR (RRID:SCR_013731) and EXRC as well as by Xenbase (http://www.xenbase.org/, RRID:SCR_003280) [67]. This work was done in compliance with German animal protection laws and was approved under Registrier-Nr. G-18/76 and G-22/43 by the state of Baden-Württemberg.

### Note on data contained in a previous preprint

Data shown here in Figs 5A, S4A, and S8D are included in preprinted work addressing the Notch regulation of mucociliary cell fates [46]. While currently still part of this preprinted article, these data will be removed from subsequent updates to reflect the changed scope of that study. Instead, these data were incorporated into this article.

### Manipulation of *Xenopus* embryos

*X. laevis* eggs were collected and in vitro-fertilized, then cultured and microinjected by standard procedures [68,69]. Embryos were injected with MOs (Gene Tools), mRNAs, or plasmid DNA at two-cell to eight-cell stage using a PicoSpritzer setup in 1/3× Modified Frog Ringer’s solution (MR) with 2.5% Ficoll PM 400 (GE Healthcare, #17-0300-50), and were transferred after injection into 1/3× MR containing Gentamycin. Drop size was calibrated to about 7–8 nL per injection. *X. laevis* allotetraploid and contains.L and.S alleles for most genes.

Embryos injected with hormone-inducible constructs of (GFP-ΔN-tp63-GR and MCI-GR) [45,58] were treated with 10 µM Dexamethasone (Sigma-Aldrich/Merck #D4902) in ethanol from eight-cell stage until fixation. Ultrapure Ethanol (NeoFroxx #LC-8657.3) was used as vehicle control.

MOs were obtained from Gene Tools targeting *dmrt2*, *foxi1*, and *ubp1*. Foxi1 MO concentrations are indicated in the figures, dmrt2 and ubp1 MO concentrations are indicated in the list below (Table 1).

**Table 1:** Morpholino sequences and doses.

Name	Sequence	Concentration Range
dmrt2 MO	5′-GTGCCTTCATCTCGTACATCTCCAG-3′	1–1.5 pmol (or 1.0–1.6 ng/nl)
foxi1MO	5′-GTGCTTGTGGATCAAATGCACTCAT-3′	0.5–3 pmol (or 0.5–3.2 ng/nl)
ubp1 MO	5′-TTGGGTCGGACAGGTACAATAATCC-3′	3 pmol (or 3.2 ng/nl)

mRNAs encoding, *foxi1* (25–100 ng/μl), *gfp-foxi1* (5–50 ng/μl), *ubp1 (50* ng/μl), *and dmrt2 (50* ng/μl) (this study; cloned using primers listed in Table 2 into pCS107), *mcidas* (100 ng/μl) [58], *foxj1* (100 ng/μl) [70], *foxa1* (100 ng/μl) [71,72], Δ*N-tp63* (100 ng/μl) [45] were injected together with *membrane-gfp* or *membrane-rfp* (at 50 ng/μL) or *h2b-rfp* (at 30 ng/μL) as lineage tracers. All mRNAs were prepared using the mMessage Machine kit using Sp6 (Invitrogen #AM1340) supplemented with RNAse Inhibitor (Promega #N251B). For rescue experiments, fusion of the *gfp*-sequence 5′ to the *foxi1* coding region rendered the mRNA insensitive to translational blocking by *foxi1* MO (the ATG of the full mRNA is >25 bases upstream of the MO binding sequence), and in the cases of *ubp1* and *dmrt2* mRNAs, only the coding regions were used, leading to no targeting sequence for *ubp1* MO and no sufficient targeting sequence (10 of 25 bases) for *dmrt2*.

**Table 2:** Full length *foxi1*, *gfp-foxi1*, *ubp1*, *and dmrt2* cloning (3′-5′).

Name	Sequence
Cla1-XLfoxi1-F	AAAAAAATCGATATGAGTGCATTTGATCCACAAGC
XLfoxi1-Sal1-R	AAAGTCGACTTATACTTCTGTACCTTCTCTG
Cla1-gfp-F	AAAAAAATCGATATGAGTGCATTTGATCCACAAGC
Cla1-gfp-R	AAAGTCGACTTATACTTCTGTACCTTCTCTG
BamH1-XLdmrt2-F	AAAGGATCCATGTACGAGATGAAGGCACC
XLdmrt2-Sal1-R	AAAGTCGACTTACTGACTAGAACGCTTGAC
BamH1-XLubp1-F	AAAGGATCCATGTTGTTTTGGCACAGCCAAC
XLubp1-Sal1-R	AAAGTCGACTCATCGCAGGATGCAGTGAAAG

The *foxi1::gfp-utrophin*, *foxi1*Δ*Foxi2BR::gfp-utrophin* and *a-tub::mscarletI* plasmids were cloned using primers listed in Table 3 and purified using the Pure Yield midiprep kit (Promega #A2492) and injected at 5 ng/μl.

**Table 3:** Cloning primers *foxi1.S* reporter (3′-5′).

Name	Sequence
gDNA-Prom_foxi1_F	GCATAATGAATCCCAAGTGTACTG
gDNA-Prom_foxi1_R	GAAGCAATCGTTTAGAGACAGG
Foxi1prom_F	CGCTATTACGCCAGTCGACCGCATAATGAATCCCAAGTG
Foxi1prom_R	CAATTCGAATCGATGGGATCAGTTAAAGCTAGCAGGTC
MS2_rmATUB_F	GATCCCATCGATTCGAATTG
MS2_rmATUB_R	GGTCGACTGGCGTAATAG
Q5foxi1Del1_F	TCTGTAGCTGATGTCTATAATC
Q5foxi1Del1_R	TACCACTGTGTGACTCAG
MS2_rmGFPUtro_F	GTACAAGTAACCTCTAGAACTATAGTGAGTC
MS2_rmGFPUtro_R	TGCTCACCATGGTTTGGATCAATTCGAATC
mScarletI_F	GATCCAAACCATGGTGAGCAAGGGCGAG
mScarletI_R	GTTCTAGAGGTTACTTGTACAGCTCGTCCATG

### *foxi1.S* reporter construct cloning and experiments

To generate the *foxi1.S::gfp-utrophin* reporter construct, genomic DNA was prepared from *X. laevis* using the phenol/chloroform DNA purification (ThermoFisher #15593031 and associated protocol). A 2.7 kb fragment (S1B and S1C Fig) of the *foxi1.S* promoter was cloned using Easy-A Hi-Fi Cloning Enzyme (Agilent #600404) and primers listed in the table below. The PCR fragment was ligated using the pGEM-T Easy Vector System (Promega #A1360). The *foxi1.S* promoter sequence was subcloned into *a-tub::gfp-utrophin* (used in [27]) after removal of the *a-tub* promoter sequence using HiFi DNA Assembly (NEB #E2621S) and Q5 High-Fidelity DNA Polymerase (NEB #M0491S) kits. *foxi1*Δ*Foxi2BR::gfp-utrophin* reporter version (S1B and S1C Fig) was generated using Q5 High-Fidelity DNA Polymerase and primers listed in the table below. The *a-tub::mscarletI* reporter was generated by replacing the *gfp-utrophin* sequence in *a-tub::gfp-utrophin* by the *mscarletI* sequence using HiFi DNA Assembly and Q5 High-Fidelity DNA Polymerase and primers listed below. Final construct sequences were analyzed by whole-plasmid nanopore sequencing.

### Real-time quantitative PCR

Total RNA was extracted from 13 to 32 animal caps per sample at stage 10.5 to 11 in experiments shown in (S5A Fig) or 14 to 20 animal caps per sample at stage 12 for experiments shown in (S6B Fig) using a standard Trizol (Invitrogen #15596026) protocol and used for cDNA synthesis with iScript cDNA Synthesis Kit (Bio-Rad #1708891) (S5A Fig) or iScript gDNA clear cDNA Synthesis Kit (Bio-Rad #1725034) (S6B Fig). qPCR reactions were conducted using Sso Advanced Universal SYBR Green Supermix (Bio-Rad #172-5275) on a CFX Connect Real-Time System (Bio-Rad) in 96-well PCR plates (Brand #781366). Expression values were normalized against two housekeeping control genes—EF1 and ODC (2^∆∆pr^ method) [53]. For reporter activity test, expression levels were also normalized for injection levels (*memRFP* mRNA). Results are presented as log-transformed fold expression over average uninjected control sample values (S5A and S6B Figs) or over average full-length foxi1-reporter construct expression levels (S6B Fig). Epidermal keratin (*krt14.4.S*; [73]) was used as pan-epidermal identity marker and *ubp1.L* was used as first selective ISC marker [3]. Primers designed to amplify *foxi1.L* or *foxi1.S* 3’UTR sequences were used to approximate endogenous *foxi1* expression after *foxi1* mRNA overexpression. Used primers are listed in Table 4.

**Table 4:** qPCR primers (3′-5′).

Name	Sequence
Ubp1.L-F	TACATCGCCACAGATACACC
Ubp1.L-R	CAGAGAAGAGATCAGCCACC
Krt12.4.S-F	AACAGCTTCTCCAGATTAGGTC
Krt12.4.S-R	ACTGAACTTGCCTTTGACACTG
Ef1a-F	CCCTGCTGGAAGCTCTTGAC
Ef1a-R	GGACACCAGTCTCCACACGA
Odc-F	GGGCTGGATCGTATCGTAGA
Odc-R	TGCCAGTGTGGTCTTGACAT
GFP-Utro-F	GTGAACTTCAAGATCCGCCA
GFP-Utro-R	AATTCGTTCTGCCCATTGTC
memRFP-F	CGGCGAGTTCATCTACAAGG
memRFP-R	ATCTTGATCTCGCCCTTCAG
Foxi1.L-3′-F	ATAGCAGTCACGACAAAGTCCT
Foxi1.L-3′-R	ACTTACTTAATACAGTGCCCTTCC
Foxi1.S-3′-F	GACTAATAGCAGGGTGGGAC
Foxi1.S-3′-R	CATCAGGTGGTTACAGTATAAAGG

### Whole-mount in situ hybridization and sections

For antisense in situ hybridization probes*, slc26a4*, *slc4a1*, *ubp1*, and *dmrt2* fragments were cloned from whole-embryo cDNAs derived from stages between 3 and 30 using primers listed in Table 5. All sequences were verified by Sanger sequencing. In addition, the following, previously published probes were used: *foxi1* [3], *foxj1* and *mcidas* [58,70], *foxa1* [72], *tp63* [45], *atp6v1e1* [53], and *dll1* [27].

**Table 5:** Probe cloning primers (5′-3′).

Name	Sequence
ISH-dmrt2-F	CAAACCAGTGTATCAGAGAC
ISH-dmrt2-R	ACTCCTTTCCTAAGAAGCAG
ISH-ubp1-F	ATTCCTGAAGCAAGAAGACC
ISH-ubp1-R	GAGAATGTGAATCCCATGAG
ISH-slc26a4-F	GATTCATACCACCTATGACAC
ISH-slc26a4-R	TTCCAATAGTTCCCTAGATTCC
ISH-slc4a1-F	ATCTCCTATCTCACCTTTCAC
ISH-slc4a1-R	ATCCATCTGTCTGTCTTCTC
ISH-anti-GFP-F	ATGGTGAGCAAGGGCGAGG
ISH-anti-GFP-R	CTTGTACAGCTCGTCCATGCCATGCCGAGAGTG

Embryos were fixed in MEMFA (100 mM MOPS pH7.4, 2 mM EGTA, 1 mM MgSO_4_, 3.7% (v/v) Formaldehyde) overnight at 4 °C and stored in 100% Ethanol at −20 °C until used. DNAs were purified using the PureYield Midiprep kit (Promega #A2492) and were linearized before in vitro synthesis of anti-sense RNA probes using T7 or Sp6 polymerase (Promega, #P2077 and #P108G), RNAse inhibitor, and dig-labeled rNTPs (Roche, #3359247910 and 11277057001). Embryos were in situ hybridized according to [74], bleached after staining with BM Purple (Roche #11442074001), and imaged. Sections were made after embedding in gelatin-albumin with Glutaraldehyde at 50–70μm as described in [75].

### TUNEL

Embryos were fixed at stage 9–10 in 1× MEMFA (100 mM MOPS pH7.4, 2 mM EGTA, 1 mM MgSO_4_, 3.7% (v/v) Formaldehyde) overnight at 4 °C or for 2 h at RT, and stored in 100% Ethanol at -−20 °C until use. Embryos were bleached before staining. TUNEL staining was performed as described in [27] using Terminal Deoxynucleotidyl Transferase Kit (Invitrogen #10533065), dig-UTP (Roche, #3359247910), anti-Digoxigenin AP Fab fragments (Roche, #11093274910), and NBT/BCIP (Roche, #11681451001). Staining was stopped with 100% Methanol (Roth, #8388.2), samples were then fixed briefly with 4% PFA (Roth, #0335.1) in PBS (Phosphate buffered saline, 10 mM Na_2_HPO_4_, 1.8 mM KH_2_PO_4_, 137 mM NaCl, 2.7 mM KCl) and imaged on a Zeiss Stemi508 with Axiocam208-color. Images were adjusted for color balance, brightness, and contrast using Adobe Photoshop. Stage 43 tadpoles served as positive control samples for successful TUNEL staining.

### Evaluation of WMISH staining and morphological evaluations

Embryos were staged according to Nieuwkoop and Faber (1994) Normal Table of *X. laevis* (Daudin). Garland Publishing Inc, New York ISBN 0-8153-1896-0. For the *foxi1* expression stage series wt embryos from multiple batches were mixed and at least 5 embryos per stage were assessed (Figs 1D and S1A). Images of embryos after in situ hybridization and corresponding sections were imaged using a Zeiss AxioZoom setup, Zeiss AxioImager.Z1 or Zeiss Stemi508 with Axiocam208-color, and images were adjusted for color balance, brightness and contrast using Adobe Photoshop.

In Figs 4C–4G, S7D, and S7E, embryos were quantified by selecting a region of interest (ROI) from each image of a stained embryo, and using the following FIJI/ImageJ adjustments processes to calculate the stained area: Color Deconvolution (H DAB), Convert to Mask, Analyze Particles. Macros used for this are available at www.github.com/sarahbowden/Imaging_Macros. In cases where the resulting files did not sufficiently represent the stained area of the ROI, this was generated manually with a combination of Brightness and Contrast, Color Deconvolution, Shadow, Threshold, or Color Thresholding techniques to select the most representative mask of stained regions. Since rescue or gain-of-function using *foxi1*, *ubp1*, and *dmrt2* mRNAs lead to broad induction of target gene expression, we used stained area as measure presented throughout the main and supplemental figures. For this, we used following macro version v1.0.0 (https://doi.org/10.5281/zenodo.17688453). To validate that area is a good proxy measure for cell numbers, we modified the parameters (i.e., particles with an area of min. 30 px and max. 500 px units were included), re-run the analysis on control and morphant samples only, and generated results of area and cell counts (Supporting information count comparison) using macro version v1.1.0 (https://doi.org/10.5281/zenodo.17688454). This confirmed that area is a suitable proxy for cell numbers, but also revealed that minor parameter changes can influence the statistical outcomes of automated analyses. Quantified data was then plotted using a custom R script which performed the Wilcoxon Rank Sum test to calculate p-values and generated plots using ggplot2. In Figs 5A and S8D, induction of expression was scored. In Fig 5B, *dll1* expression in the ventral epidermis was analyzed as normal or less (number of dots and expression intensity). In Fig 5C, expression level differences observed between the uninjected control sides and manipulated sides of embryos were scored in whole-mount embryos, while depicted sections are shown for clarity.

For analyses in Figs 3B and S4A, embryos injected with high dose of *foxi1* MO, cell morphology and cell size were evaluated for S4A Fig (and delamination was confirmed in hemisected embryos) and skin lesions were evaluated for Fig 3B. Gastrulation phenotypes in S4C and S4D Fig were scored in different treatment groups, color code of graph and representative examples of phenotypes are presented in S4D Fig. WMISH marker staining for ISCs (ubp1) and MCCs (mcidas) (S4F Fig) were analyzed in the same set of embryos used for scoring of gastrulation defects S4C and S4D Fig). The upper panels show marker expression in representative examples for each class of phenotypes in control and *foxi1* morphant embryos. Bottom rows show examples of morphants as well as morphants co-injected with different concentrations of *gfp-foxi1* mRNAs (two examples per marker and rescue mRNA concentration).

### Immunofluorescence staining, in situ hybridization chain reaction and sample preparation

Whole *Xenopus* embryos, were fixed at indicated stages in 4% paraformaldehyde at 4 °C overnight or 2 h at room temperature, then washed 3 × 15 min with PBS, 2 × 30 min in PBST (0.1% Triton X-100 in PBS), and were blocked in PBST-CAS (90% PBS containing 0.1% Triton X-100, 10% CAS Blocking; ThermoFischer #00-8120) for 30 min-1 h at RT. A detailed protocol was described in [30].

Mouse anti-Acetylated-α-tubulin (Sigma/Merck #T6793) primary antibody (1:1,000) was used to mark cilia/MCCs, Rabbit Anti-serotonin (Sigma/Merck #S5545) primary antibody (1:500) was used to mark SSCs, Rabbit Anti-GFP (Invitrogen #A11122) primary antibody (1:400) was used after HCR to boost *foxi1*::gfp-utrophin signal and applied at 4 °C overnight. Secondary antibodies AlexaFluor-405-labeled goat anti-mouse (Invitrogen # A30104), AlexaFluor 405-labeled goat anti-rabbit antibody (Invitrogen #A31556), and AlexaFluor-488-labeled goat anti-rabbit antibody (Invitrogen #A11008) were used for 2 h at RT (1:250). Antibodies were applied in 100% CAS Blocking (ThermoFischer #00-8120). Actin was stained by incubation (30–120 min at room temperature) with AlexaFluor 405-labeled Phalloidin (1:800 in PBSt; Invitrogen #A30104), mucus-like compounds were stained by incubation (overnight at 4 °C) with AlexaFluor-594- or -647-labeled or PNA (1:500–1,000 in PBSt; Molecular Probes #L32459 and #L32460).

For HCR, *Xenopus* embryos were fixed at indicated stages in 10% MEMFA (100 mM MOPS pH7.4, 2 mM EGTA, 1 mM MgSO4, 3.7% (v/v) Formaldehyde) for 1 h at RT, washed with PBSTw and stored in 100% Methanol at −20°C until use. HCR (hybridized chain reaction) was performed as previously described [76]. *foxi1* probe and amplifiers were designed and obtained from Molecular Instruments, (https://www.molecularinstruments.com/). IF staining was performed on samples after HCR following the steps described above.

### Fluorescence imaging, image processing, and analysis

Confocal imaging was conducted using either a Zeiss LSM880 or a Zeiss LSM980 microscope and Zeiss Zen software in the LIC and BiMiC imaging facilities. Confocal images were adjusted for channel brightness/contrast, Z-stack projections were generated and cell types were quantified based on their morphology using ImageJ [77]. For analyses in Figs 3D and 5D, a detailed protocol for quantification of *Xenopus* epidermal cell type composition was published [30].

For analysis and comparison of fluorescent reporter construct activity on confocal micrographs (S5D, S5E, and S6C Figs) in ImageJ, z-projections were performed using the “sum-slices” function. Analysis of reporter activity after *foxi1* MO knockdown (S5D and S5E Fig) was conducted by serial injections at 2- and 4-cell stages. First both blastomeres were animally injected with *foxi1::gfp-utrophin* construct together with mRNA encoding H2B-RFP leading to label reporter-targeted cells by nuclear RFP expression. Next, one ventral cell was injected at 4-cell stage with 3 pmol *foxi1*MO together with mRNA encoding memRFP to mark morphant cells by membrane-RFP expression (S6C Fig). At stage 19, embryos were fixed in 4% PFA overnight, washed, and stained using phalloidin (as described above). Next, embryos were sectioned manually and transversal sections were imaged using tile-scanning on a confocal microscope (Zeiss LSM980). Tiles were stitched using ImageJ. H2B-RFP (+) cells with and without co-staining by membrane-RFP (morphant cells) were analyzed for GFP-Utrophin signal in the deep layer of the epidermis. Induction of reporter expression in the endoderm (S6A Fig) embryos were imaged using a Zeiss AxioImager.Z1 with Axiocam208-color camera. Induction was scored as positive when GFP fluorescence was detected in the vegetal half of the gastrula embryo. In some controls, activity was observed in involuting or animally positioned mesoderm, where maternal *foxi2* deposition occurs.

### Western blot analysis of GFP-Foxi1 levels

Eight to fifteen embryos per condition were collected and stored at −80 °C until use. Embryos were lysed in 100 µl of 1× Lysis Buffer (20 mM Tris-HCl pH8, 150 mM NaCl, 2 mM EDTA, 1× Protease Inhibitor Roche, #04693116001, 1% NP40 Sigma, #I8896) and smashed by pipetting up and down, then the samples were centrifuged at 4 °C at maximum speed for 15 min to remove yolk. 4× Laemmli Buffer (50 ml 4× buffer, 1 M Tris, pH 6.8, 4 g SDS, 20 ml Glycerol, 10 ml 2-Mercaptoethanol, 0.1 g Bromophenol Blue) was added to the supernatant and the samples were cooked at 95 °C for 10 min.

Ten percent separating gel (2.5 ml 4× Tris SDS (Roth, #2326), pH 8.8, 2 ml 40% Acrylamide (Sigma, #A7802), 5.4 ml H_2_O, 40 µl TEMED (Roth, #2367.1), 100 µl 10% APS (Roth, #9592.2)) was used, and a 4% collecting gel (1.25 ml 4× Tris SDS, pH 6.8, 0.625 ml 40% Acrylamide, 3.11 ml H_2_O, 50 µl TEMED, 50 µl 10% APS)). 1× Running buffer (25 mM Tris-HCl, pH 8, 192 mM Glycine (Roth, #3187) in distilled water) was used for electrophoresis. Ten µl Precision Plus Protein Western C Standards Ladder (BioRad; #161-0376) and 20 µl of each sample were loaded for electrophoresis (120 V for 90 min).

Semi-dry transfer onto an activated PVDF membrane (Thermo Scientific, #88518) was conducted in 1× Towbin buffer with 0.1% SDS for 60 min (25 mM Tris-base, 192 mM Glycine, 1% SDS) using a PerfectBlue Semi-Dry Electroblotter Sedec M (VWR; 700–1,220). Membranes and Gels were washed in 1× TBStw (100 mM Tris-base, 500 mM NaCl, 1% Tween 20) at RT.

Membranes were blocked for at least 45 min using 5% non-fat dry milk (Roth, #T145.3) in TBStw. The following primary antibodies were used at 1:1,000 and incubated over night at 4 °C: Rabbit monoclonal anti-GFP (Abcam, #ab290) and, as a loading control, mouse monoclonal alpha-Tubulin (Thermo Scientific, DM1A #62204). The membrane was then washed in 1× TBStw for 4 × 20 min. The following secondary antibodies were used at 1:5000 and incubated for 2 h at RT: HRP-linked Anit-Mouse IgG (Cell Signaling, #7076) and HRP-linked Anti-Rabbit IgG (Cell Signaling, #7074). The membrane was washed in 1× TBStw for 6 × 10 min. Membranes were incubated with a mixture of 500 µl of Peroxide solution and 500 µl of Luminol/enhancer solution (both from Clarity Western ECL Substrate (BioRad, #170–5,061) for 5 min in the dark at room temperature. Membranes were imaged using the Odyssey XF Imaging System by LI-COR. Afterwards, membranes were washed and stored in TBStw. Membranes were stripped for loading control (α-Tubulin) re-probing with stripping Buffer (2% SDS, 50 mM Tris pH 6.8, 100 mM 2-Mercaptoethanol) for 1 h at 50 °C. The membranes were again rinsed in distilled water and washed 3 × 5 min with TBStw. Brightness and contrast were adjusted in image J, and the ladder image was added to the chemi-luminescence membrane image.

### RNA- and ATAC-sequencing on *Xenopus* mucociliary organoids and bioinformatics analysis

Manipulations and bulk mRNA-seq used in this paper were generated and published here [45,46] (GSE130448, *n* = 3 per time point and condition; GSE215373, *n* = 2 per time point and condition; GSE215419, *n* = 2 per time point; GSE262944, *n* = 2 per time point) or generated for this study (*foxi1* MO, *ubp1* MO, *dmrt2* MO on animal cap organoids at stages 20 and 32, *n* = 3 per time point and condition; GSE299718). scRNA-seq datasets were published here: [55,59].

Data for Figs 4B and S6A–S6C was generated from 10 to 15 pooled animal caps per sample and time point (3 replicates each), collected in Trizol for total RNA isolation. Library preparation and RNA-seq (150 base paired-end reads, min. 15 Mio. reads per sample) were performed in collaboration with the NIG, University Medical Center Göttingen using standard protocols described in [45,46]. RNA-seq data generated for this study was deposited at NCBI GEO under (GSE299718).

For Fig 3A, data from [46] were used, TPM values from.L and.S allo-allels were added, and the resulting matrix was clustered using Z-values per line and galaxy.eu (ggplot2_heatmap2/3.1.3.1+galaxy). For S8A–S8C Fig, log2-fold changes were calculated using galaxy.eu (DeSeq2/2.1.3+galaxy) and visualized using (ggplot2_heatmap2/3.1.3.1+galaxy). For S6C Fig, the online tool associated with [55] was used (marionilab.cruk.cam.ac.uk/XenopusRegeneration). For S8E Fig, the online tool associated with [59] was used (kleintools.hms.harvard.edu/tools/currentDatasetsList_xenopus_v2.html) to extract lineage-enriched transcripts and the heatmap was generated using galaxy.eu (ggplot2_heatmap2/3.1.3.1+galaxy).

For ATAC-seq sample generation, injected and control embryos were cultured until st. 8. Animal caps were dissected in 1× Modified Barth’s solution (MBS) and transferred to 0.5× MBS + Gentamycin [31]. 2 organoids per condition and replicate were collected in PBS and ATAC-seq was performed as described in [78,79]. In short: Embryos were injected bilaterally in the animal hemisphere at the two-cell stage with 3 pmol *foxi1* MO or remained uninjected, animal caps were prepared at st. 8, and organoids were collected upon the appearance of the dorsal lip in control embryos cultured in parallel to the organoids (st. 10). Organoids were transferred from MBS plates into a 1.5 mL low-bind microcentrifuge tube (Eppendorf #0030108051) containing 1 mL of ice-cold 1× PBS. Samples were spun at 500*g* at 4 °C in the centrifuge for five minutes before removing the PBS and repeating the wash step with fresh ice-cold 1× PBS. Fifty µL of ice-cold lysis buffer (10 mM Tris pH 7.4, 10 mM NaCl, 3 mM MgCl_2_, 0.1% (w/v) Igepal CA-630) and pipetted to break up samples. Samples were centrifuged at 500*g* for 10 min at 4 °C and pellets were resuspended in 25 µL TD Buffer, 2.5 µL TDE1 Enzyme, and 22.5 µL Nuclease-Free water (Illumina #20034198). Samples were pelleted to mix and incubated on a ThermoMixer at 37 °C, 700 rpm for 30 min. Following incubation, the samples were cleaned with MinElute Reaction Cleanup Kit (Qiagen, #28204), following manufacturer instructions and eluted into 11 µL Buffer EB.

Libraries were prepared in collaboration with the NIG, University Medical Center Göttingen. Quality was assessed with the Agilent Fragment Analyzer and prepared with the ATAC-seq Kit (Active Motif, #53150). Samples were sequenced in triplicate on an Illumina NovaSeq6000 with 150-nucleotide paired-end reads, totaling 50 million reads per sample.

Raw sequencing files were assessed for quality using FastQC (v0.11.9, Andrews, S. FastQC A Quality Control tool for High Throughput Sequence Data. http://www.bioinformatics.babraham.ac.uk/projects/fastqc/), and adapter sequences were removed with TrimGalore (v0.6.7, https://doi.org/10.5281/zenodo.7598955). Data were aligned to the *X. laevis* genome assembly v9.2 using BWA-MEM (v0.7.17, https://arxiv.org/abs/1303.3997). Mitochondrial reads were removed using Samtools (v1.21) [80], and peak calling was performed with the callpeak function of MACS2 (v2.2.7.1) [81]. Differential analysis was performed with the bdgdiff function of MACS2 (parameters: length—200 bp; gap—100 bp; cutoff—3 with likelihood ration = 1,000) and Venn diagrams were generated with VennDiagram v1.7.3 in R v4.4.1. Heatmaps showing the average ATAC-seq signal were generated using deepTools (v3.5.4) [82]. Peaks were annotated for the nearest *X. laevis* gene and transcription factor binding motifs with Homer (v4.11) [83], plant-specific transcription factors were manually excluded from the lists of transcription factors. In Fig 2E and 2F, each peak in the analysis was annotated to the nearest coding gene using Homer (v4.11). The peaks which were nearest to mucociliary-specific genes (specific for ISCs, MCCs, and basal cells published in [44,45]) were then identified. Venn diagrams were then made for each mucociliary cell type, comparing the number of peaks lost, maintained, or gained for each cell type. Bioinformatic analyses were performed on the Galaxy/ Europe platform (usegalaxy.eu) [84]. ATAC-seq data generated for this study was deposited at NCBI GEO under (GSE280790).

### Quantification and statistical evaluation

Stacked bar graphs were generated in Microsoft Excel. Heatmaps and Venn diagrams were generated using the Galaxy Europe platform (usegalaxy.eu) and R. Statistical tests used and significance levels are indicated in the figure legends, and were conducted in Excel or R or using https://astatsa.com/OneWay_Anova_with_TukeyHSD (ANOVA, S5A Fig). Sample sizes for all experiments were chosen based on previous experience and used embryos derived from at least two different females. No randomization or blinding was applied.

### Use of shared controls

For some of the in situ and IF experiments shared controls were used in multiple graphs; all experiments are listed in S7 Data.

## Supporting information

S1 Fig Early Foxi1 expression and reporter constructs. **(A)** WMISH expression analysis of *foxi1* across mucociliary epidermis development stages (st. 9–32). St. 9, 10 = animal views; st. 12, 16 = ventral views; st. 25, 32 = lateral views, anterior to the left. Ectodermal (st. 9–10) and epidermal (st. 12–32) regions are outlined in yellow. Bottom row panels = magnified views of epidermal areas. **(B,C)** Generation and promoter sequences of *foxi1::gfp-utrophin* or *foxi1ΔFoxi2BR::gfp-utrophin* reporters. **(B)** Schematic representation of cloned genomic *foxi1.S* promoter locus (gray box) and position of Foxi2 binding region determined in Cha and colleagues, 2012 (black outlined box). **(C)** Promoter sequence with indicated predicted core Foxi binding motifs (yellow) and Foxi2 binding region (bold, underscored).
(TIF)

S2 Fig Characterization of the foxi1 reporter. **(A,B)** IF of embryos injected with *foxi1::gfp-utrophin* (green) (*n* = 12 embryos) and α-tub.::mscarletI (magenta) (*n* = 9 embryos) reporters at st. 32 stained for Acetylated-α-tubulin (Ac.-α-tub., cilia, gray), F-actin (Actin, cell borders and morphology, gray), and serotonin (SSCs, gray) in **(A)**; or for Acetylated-α-tubulin (Ac.-α-tub., cilia, gray) and F-actin (Actin, cell borders and morphology, gray), in **(B)**. In **(B)**, targeted cells were identified by nuclear RFP expression (H2B-RFP, magenta). **(C)** WMISH expression analysis of *foxi1::gfp-utrophin* (stained for *gfp* transcripts) across mucociliary epidermis development stages (st. 9–32). St. 9, 10 = animal views; st. 12, 16 = ventral views; st. 25, 32 = lateral views. Bottom row panels = magnified views of epidermal areas. Related to sections shown in Fig 1D. st. 9 *n* = 17; st. 10 *n* = 19; st. 12 *n* = 16; st. 16 *n* = 14; st. 25 *n* = 14; st. 32 *n* = 19 embryos.
(TIF)

S3 Fig Foxi1 reporter expression, morpholino targets, ATAC-profiles. **(A)** IF for *foxi1::gfp-utrophin* reporter (green) and F-actin (Actin, cell borders and morphology, magenta) at st. 12–20 on hemisected embryos. Targeted cells were identified by membrane RFP expression (memRFP, gray). Related to sections shown in Fig 1E. st. 12 *n* = 5; st. 14 *n* = 4; st. 20 *n* = 5 embryos. **(B)** Alignment of MO-target sequences in *foxi1*, *ubp1*, and *dmrt2* transcripts. ATG start-codons are indicated by yellow boxes. Generated with http://multalin.toulouse.inra.fr. **(C)** Distribution of accessible regions around genes required for development and cell fates specification in the embryonic mucociliary epidermis of *Xenopus*. Lost, maintained, and gained tracks as generated by MACS2 bdgdiff analysis and visualized in IGV: *dll1.L; ubp1.L; dmrt2.S; foxj1.L;* and *tp63.L.* Turquoise track = control (ctrl.) and purple track = morphant (*foxi1* MO). *n* = 2 organoids per condition and replicate. Three replicates. Related to Fig 2D.
(TIF)

S4 Fig Concentration-dependent effects of Foxi1 manipulations. **(A)** Representative brightfield images of controls (ctrl.) and embryos (animal views) after *foxi1* MO (3 pmol) injection at st. 8. Morphants showed enlarged cells and delamination of animal cells into the blastocoel. Quantification of results shown in the graph. Delamination events were scored based on morphological analysis. *n* = number of embryos. Chi^2^ test: *p* < 0.001 = ***. **(B)** TUNEL staining to identify apoptotic cells. Representative images of controls (ctrl.) and embryos (animal views) after *foxi1* MO (3 pmol) injection at st. 9–10. Quantification of results shown in the graph. *n* = number of embryos. Chi^2^ test: *p* > 0.05 = ns. **(C,D)** Analysis and quantification of gastrulation defects at st. 19 in controls (ctrl.), *foxi1* moprhants (3 pmol), and rescued morphants by co-injection of 10 or 50 ng/μl *gfp-foxi1* mRNA. Representative examples of phenotypic classes and color code used in **(C)** are depicted in **(D)**. *n* = number of embryos. Chi^2^ test: *p* < 0.05 = *; *p* < 0.01 = **; *p* < 0.001 = ***. **(E)** Quantification of results depicted in Fig 3C. Samples were analyzed for presence (white) or absence (dark red) of intercalating cells in highly targeted areas as well as for the presence of ISC-like cells (turquois). **(F)** WMISH analysis of ISC (*ubp1*) and MCC (*mcidas*) marker expression in st. 19 embryos used in (C,D). Dorsal up, anterior to the right. Upper two rows show representative examples of control (ctrl.) and all morphological classes of *foxi1* MO (3 pmol) injected embryos. The bottom two rows show representative examples of *foxi1* MO (3 pmol) injected embryos with or without co-injection of 10 or 50 ng/μl *gfp-foxi1* mRNA. Extruding mes-endodermal tissue is outlined in yellow. Large areas devoid of marker expression are indicated by red arrows. Areas showing increased expression of markers are indicted by yellow arrows. Ctrl. (ubp1/mcidas) *n* = 21/21, *foxi1*MO *n* = 34/33, *foxi1*MO + 10 ng/μl *gfp-foxi1 n* = 31/35, *foxi1*MO + 50 ng/μl *gfp-foxi1 n* = 34/35. Data used for panels (A), (B), (C), and (E): S1 Data.
(TIF)

S5 Fig Foxi1 is required for epidermal competence, ISCs and *foxi1* expression. **(A)** qPCR on pooled uninjected control organoids and after *foxi1* MO (3 pmol) with or without co-injected *gfp-foxi1* at 5, 15, or 50 ng/μl. The epidermal competence gene *krt12.4.S* and the definitive ISC marker *ubp1* show differential dose-dependent reactions to *foxi1* manipulations. ANOVA (Tukey HSD corrected): *p* > 0.05 = ns; *p* < 0.01 = **. *n* = number of biological and technical replicates. **(B)** western blot analysis of GFP-Foxi1 overexpression (anti-GFP) levels in lysates from pooled whole embryos at stage 12 in uninjected controls and embryos injected with *gfp-foxi1* at 5, 15, or 50 ng/μl. Two different batches (biological replicates) are shown. Predicted size of GFP-Foxi1 ca. Sixty-eight kDa, specific bands are indicated by yellow box, unspecific band indicated by red asterisk. Anti-Tubulin is used as loading control. **(C–E)** IF analysis of bisected st. 19 embryos injected with *foxi1::gfp-utrophin* (*n* = 9 embryos) reporter (green) into both blastomeres at 2-cell stage (identified by nuclear RFP expression; H2B-RFP, magenta), followed by injection of *foxi1* MO (3 pmol) into one ventral blastomere at 4-cell stage (identified by membrane RFP expression; memRFP, magenta). Embryos were stained for F-actin (Actin, cell borders and morphology, gray). **(D)** Comparison of *foxi1* MO targeted (left side of section) and non-targeted (right side of section) cells revealed reduced reporter activity (diminished GFP signal) after *foxi1* knockdown. **(D′ and D˝)** show magnified areas indicated by dashed yellow boxes in overview panels. **(E)** Magnification of an epidermal area where morphant- and non-morphant cells mixed shows GFP signal (yellow arrows) in reporter-only targeted cells, but reduced signal in MO-targeted cells. Data used for panel (A): S1 Data.
(TIF)

S6 Fig Foxi1 regulates its own expression and Dmrt2 is expressed only in α-ISCs. **(A)** IF analysis of embryos injected with *foxi1::gfp-utrophin* (*n* = 12 embryos) or *foxi1ΔFoxi2BR::gfp-utrophin* (*n* = 12 embryos) reporters (green) at st. 32 stained for Acetylated-α-tubulin (Ac.-α-tub., cilia, gray), F-actin (Actin, cell borders and morphology, gray), and serotonin (SSCs, gray) at st. 32. Targeted cells were identified by nuclear RFP expression (H2B-RFP, blue). **(B)** qPCR on pooled uninjected control organoids and after injection of *foxi1::gfp-utrophin* (full-length) or *foxi1ΔFoxi2BR::gfp-utrophin* (Δfoxi2BR) together with memRFP for normalization (left), and on pooled uninjected control organoids and organoids injected with 100 ng/μl of *foxi1* mRNA (right). Gray box-plots = *gfp* expression relative to full-length reporter *gfp* expression; red box-plots = *gfp* expression normalized by *rfp* expression; blue box-plots = *foxi1.L* 3′ UTR expression; purple box-plots = *foxi1.S* 3′ UTR expression. *T* test (2-tail, paired): *p* > 0.05 = ns; *p* < 0.05 = *; *p* < 0.01 = **. *n* = number of biological and technical replicates. (**C**) Brightfield and epifluorescence images of hemisected st. 11 gastrula embryos injected vegetally with *foxi1::gfp-utrophin* (green), membrane RFP (memRFP; magenta) as control (*memRFP*) or with additional co-injection of *foxi1* mRNA (*foxi1* + *memRFP*). Right panels show false-color of GFP fluorescence intensity. Induction was scored as positive when GFP was detected in areas below the equator (mesendoderm). Ctrl. *n* = 7 induced, 26 non-induced; *foxi1* mRNA = 26 induced, 11 non-induced. Embryos are shown dorsal to the left and animal up. **(D)** Boxplots of ISC gene expression from scRNA-seq data published in Aztekin and colleagues, 2019. Visualization was generated using the published online tool: marionilab.cruk.cam.ac.uk/XenopusRegeneration. Data used for panel (B): S1 Data.
(TIF)

S7 Fig Foxi1, Ubp1, and Dmrt2 differentially regulate ISC specification. **(A–C)** Effects of Foxi1 (*foxi1* MO, 1.5 pmol; **A**), Ubp1 (*ubp1* MO, 3 pmol; **B**), or Dmrt2 (*dmrt2* MO, 1 pmol; **C**) knockdown on core ISC gene expression stages 20 and 32. RNA-seq on mucociliary organoids. Heatmaps depict log2-fold change values derived from DEseq2. **(D,E)** Analysis of effects by WMISH at st. 29–32 against *atp6v1e1* and *foxi1* (pan-ISC markers), *ubp1* and *slc25a4/pendrin* (β-ISC markets), and *dmrt2* and *slc4a1/ae1* (α-ISC markers) after Ubp1 (*ubp1* MO, 3 pmol) or Dmrt2 (*dmrt2* MO, 1 pmol) knockdown, rescue and overexpression (by mRNA injections: 50 ng/µl *ubp1*; 25–50 ng/µl *dmrt2*). Representative images and quantification of results are depicted. *n* = number of embryos analyzed per condition. Wilcoxon Rank Sum test: *p* > 0.05 = ns; *p* < 0.05 = *; *p* < 0.01 = **; *p* < 0.001 = ***. Data used for panels (A), (B), (C), (D′), and (E′): S1 Data.
(TIF)

S8 Fig Notch regulation of ISC genes and ISC-subtype markers. **(A–C)** Effects of Notch gain (*nicd*; **A**), Notch loss (*suh-dbm*; **B**), and Notch and MCC loss (*suh-dbm* + *dn-mcidas*; **C**) on core ISC gene expression in key developmental stages (st. 10, 16, 25, 32). Heatmaps depict log2-fold change values derived from DEseq2. Asterisks indicate statistical significant (adj-*p* value < 0.05) changes. **(D)** Representative images of st. 9 control (ctrl.) and manipulated embryos (animal views) after mRNA overexpression of transcription factors to test premature induction of *dlc*. Quantification of results and effects on *dll1* (yellow) and *dlc* (blue) graphs. Embryos were scored as induced or non-induced expression. Related to Fig 5A. **(E)** Heatmap of mucociliary marker gene enrichment during differentiation in lineages from scRNA-seq data published in Briggs and colleagues (2018). Values were derived using the published online tool: kleintools.hms.harvard.edu/tools/currentDatasetsList_xenopus_v2.html. NNE, non-neural ectodermal precursors; BC, basal cells; ISC, ionocytes; MCC, multiciliated cells; SSC, small secretory cells; GB, outer-layer goblet cells. Data used for panels (A), (B), (C), (D), and (E): S1 Data.
(TIF)

S1 Raw Images Full western blot membranes associated with S5B Fig. (TIF)

S1 Data Quantifications used in graphs. (XLSX)

S2 Data Data used to generate Fig 2A. (ZIP)

S3 Data Motif enrichment results from Homer related to Fig 2C. (PDF)

S4 Data Motif enrichment results from Homer related to Fig 2E. (PDF)

S5 Data Comparison of automated quantification results using different parameters related to Fig 4D. (TIF)

S6 Data Data used for comparison of automated quantification results using different parameters related to Fig 4D. (XLSX)

S7 Data Experimental log. (XLSX)

## Abbreviations

EXRC: European Xenopus Resource Centre
HCR: hybridization chain reaction
IF: immunofluorescence
ISCs: ionocytes
MBS: Modified Barth’s solution
MCCs: multiciliated cells
MCE: mucociliary gene-associated
MO: morpholino oligonucleotide
MPPs: multipotent progenitors
qPCR: quantitative PCR
RNA-seq: RNA-sequencing
ROI: region of interest
SSCs: small secretory cells
WMISH: whole-mount in situ hybridization
