# Supplementary figures and images for: Foxi1 regulates multipotent mucociliary progenitors and ionocyte specification through transcriptional and epigenetic mechanisms
Source: PLoS Biol. 2026 Jan 5;24(1):e3003583. doi: 10.1371/journal.pbio.3003583 (PMC12768278; doi:10.1371/journal.pbio.3003583)

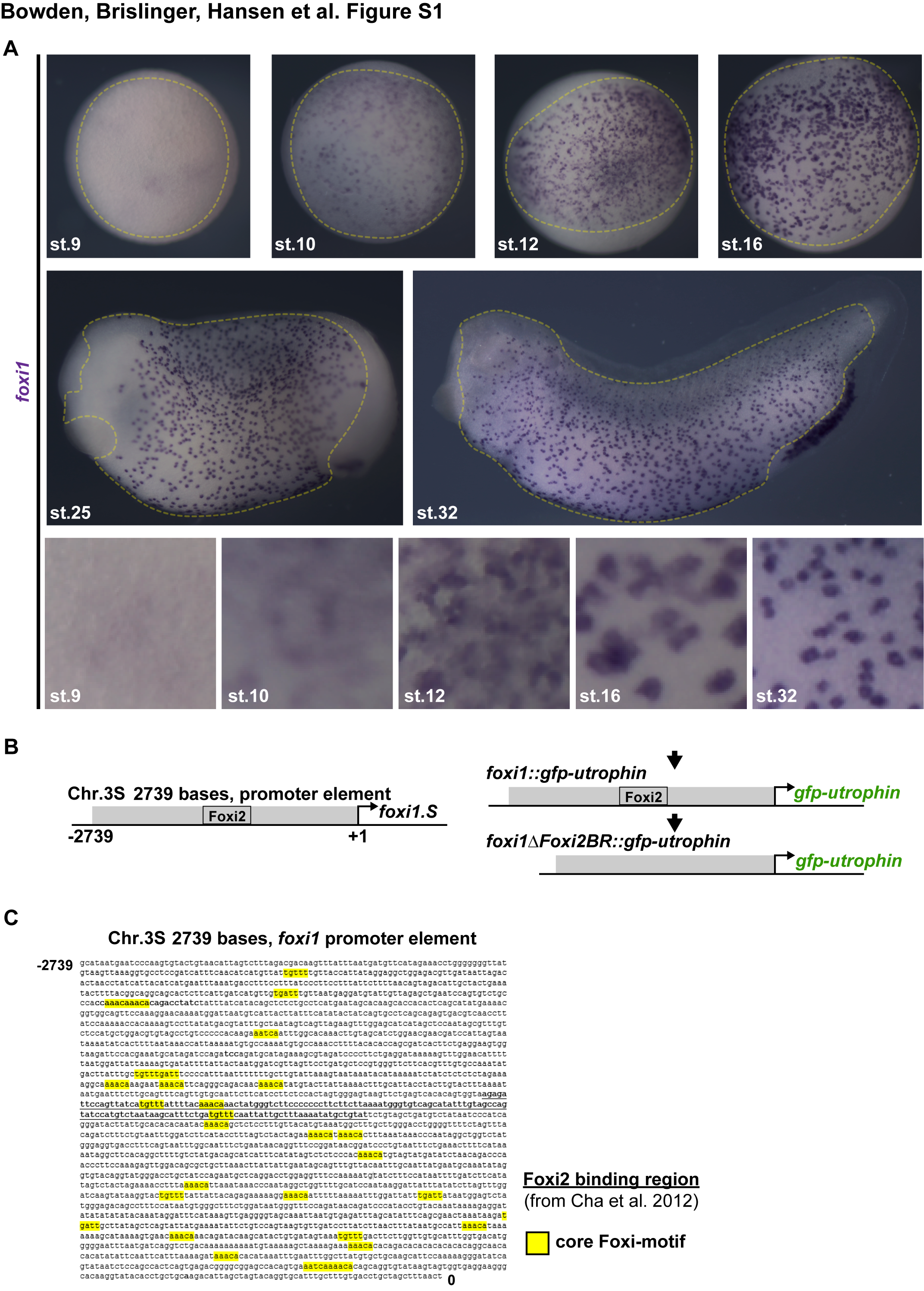

Supplement: S1 Fig — (A) WMISH expression analysis of foxi1 across mucociliary epidermis development stages (st. 9–32). St. 9, 10 = animal views; st. 12, 16 = ventral views; st. 25, 32 = lateral views, anterior to the left. Ectodermal (st. 9–10) and epidermal (st. 12–32) regions are outlined in yellow. Bottom row panels = magnified views of epidermal areas. (B,C) Generation and promoter sequences of foxi1::gfp-utrophin or foxi1ΔFoxi2BR::gfp-utrophin reporters. (B) Schematic representation of cloned genomic foxi1.S promoter locus (gray box) and position of Foxi2 binding region determined in Cha and colleagues, 2012 (black outlined box). (C) Promoter sequence with indicated predicted core Foxi binding motifs (yellow) and Foxi2 binding region (bold, underscored). (TIF) [file pbio.3003583.s001.tif]

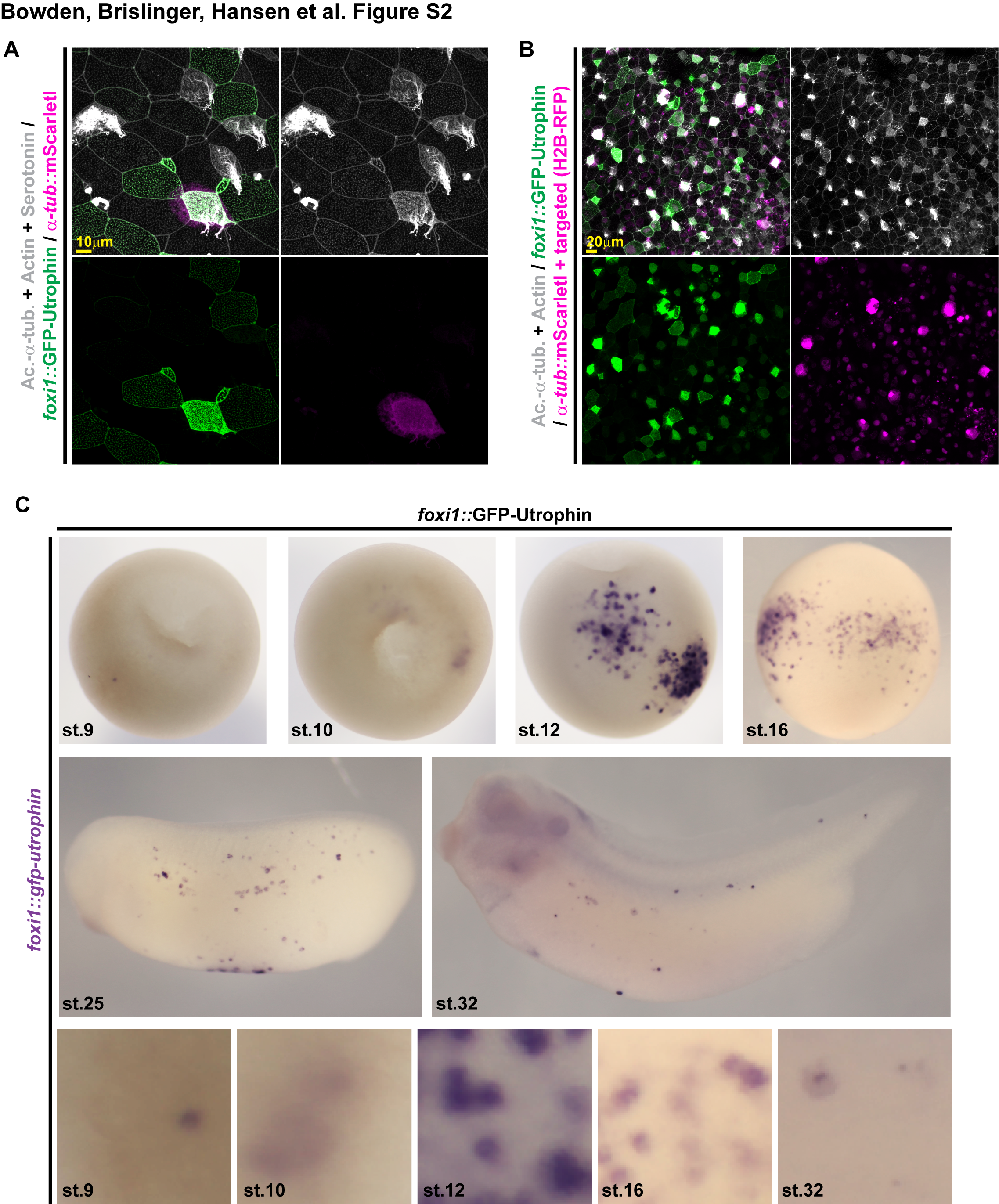

Supplement: S2 Fig — (A,B) IF of embryos injected with foxi1::gfp-utrophin (green) (n = 12 embryos) and α-tub.::mscarletI (magenta) (n = 9 embryos) reporters at st. 32 stained for Acetylated-α-tubulin (Ac.-α-tub., cilia, gray), F-actin (Actin, cell borders and morphology, gray), and serotonin (SSCs, gray) in (A); or for Acetylated-α-tubulin (Ac.-α-tub., cilia, gray) and F-actin (Actin, cell borders and morphology, gray), in (B). In (B), targeted cells were identified by nuclear RFP expression (H2B-RFP, magenta). (C) WMISH expression analysis of foxi1::gfp-utrophin (stained for gfp transcripts) across mucociliary epidermis development stages (st. 9–32). St. 9, 10 = animal views; st. 12, 16 = ventral views; st. 25, 32 = lateral views. Bottom row panels = magnified views of epidermal areas. Related to sections shown in Fig 1D. st. 9 n = 17; st. 10 n = 19; st. 12 n = 16; st. 16 n = 14; st. 25 n = 14; st. 32 n = 19 embryos. (TIF) [file pbio.3003583.s002.tif]

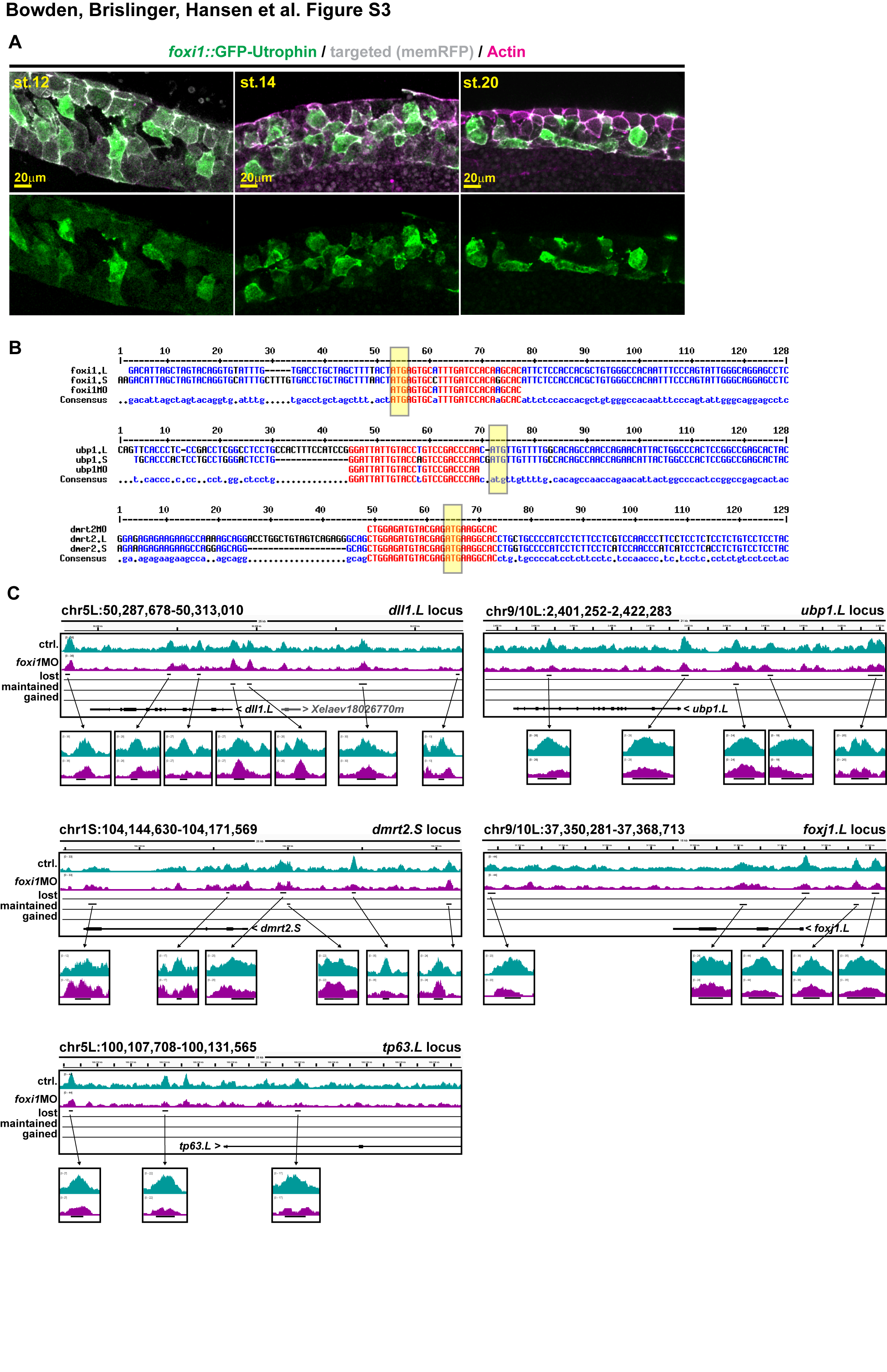

Supplement: S3 Fig — (A) IF for foxi1::gfp-utrophin reporter (green) and F-actin (Actin, cell borders and morphology, magenta) at st. 12–20 on hemisected embryos. Targeted cells were identified by membrane RFP expression (memRFP, gray). Related to sections shown in Fig 1E. st. 12 n = 5; st. 14 n = 4; st. 20 n = 5 embryos. (B) Alignment of MO-target sequences in foxi1, ubp1, and dmrt2 transcripts. ATG start-codons are indicated by yellow boxes. Generated with http://multalin.toulouse.inra.fr. (C) Distribution of accessible regions around genes required for development and cell fates specification in the embryonic mucociliary epidermis of Xenopus. Lost, maintained, and gained tracks as generated by MACS2 bdgdiff analysis and visualized in IGV: dll1.L; ubp1.L; dmrt2.S; foxj1.L; and tp63.L. Turquoise track = control (ctrl.) and purple track = morphant (foxi1 MO). n = 2 organoids per condition and replicate. Three replicates. Related to Fig 2D. (TIF) [file pbio.3003583.s003.tif]

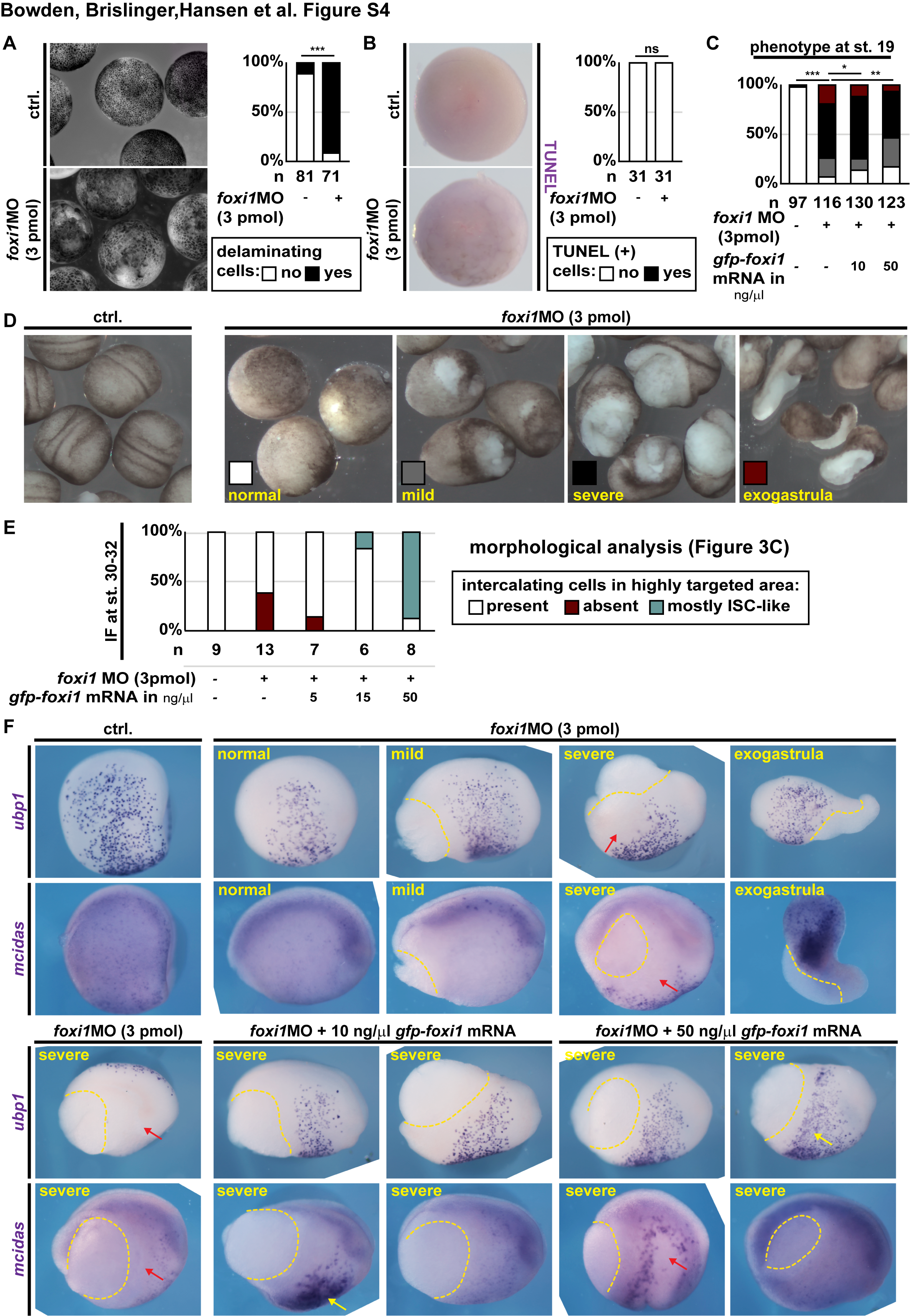

Supplement: S4 Fig — (A) Representative brightfield images of controls (ctrl.) and embryos (animal views) after foxi1 MO (3 pmol) injection at st. 8. Morphants showed enlarged cells and delamination of animal cells into the blastocoel. Quantification of results shown in the graph. Delamination events were scored based on morphological analysis. n = number of embryos. Chi2 test: p < 0.001 = ***. (B) TUNEL staining to identify apoptotic cells. Representative images of controls (ctrl.) and embryos (animal views) after foxi1 MO (3 pmol) injection at st. 9–10. Quantification of results shown in the graph. n = number of embryos. Chi2 test: p > 0.05 = ns. (C,D) Analysis and quantification of gastrulation defects at st. 19 in controls (ctrl.), foxi1 moprhants (3 pmol), and rescued morphants by co-injection of 10 or 50 ng/μl gfp-foxi1 mRNA. Representative examples of phenotypic classes and color code used in (C) are depicted in (D). n = number of embryos. Chi2 test: p < 0.05 = *; p < 0.01 = **; p < 0.001 = ***. (E) Quantification of results depicted in Fig 3C. Samples were analyzed for presence (white) or absence (dark red) of intercalating cells in highly targeted areas as well as for the presence of ISC-like cells (turquois). (F) WMISH analysis of ISC (ubp1) and MCC (mcidas) marker expression in st. 19 embryos used in (C,D). Dorsal up, anterior to the right. Upper two rows show representative examples of control (ctrl.) and all morphological classes of foxi1 MO (3 pmol) injected embryos. The bottom two rows show representative examples of foxi1 MO (3 pmol) injected embryos with or without co-injection of 10 or 50 ng/μl gfp-foxi1 mRNA. Extruding mes-endodermal tissue is outlined in yellow. Large areas devoid of marker expression are indicated by red arrows. Areas showing increased expression of markers are indicted by yellow arrows. Ctrl. (ubp1/mcidas) n = 21/21, foxi1MO n = 34/33, foxi1MO + 10 ng/μl gfp-foxi1 n = 31/35, foxi1MO + 50 ng/μl gfp-foxi1 n = 34/35. Data used for panels (A), (B), (C) [file pbio.3003583.s004.tif]

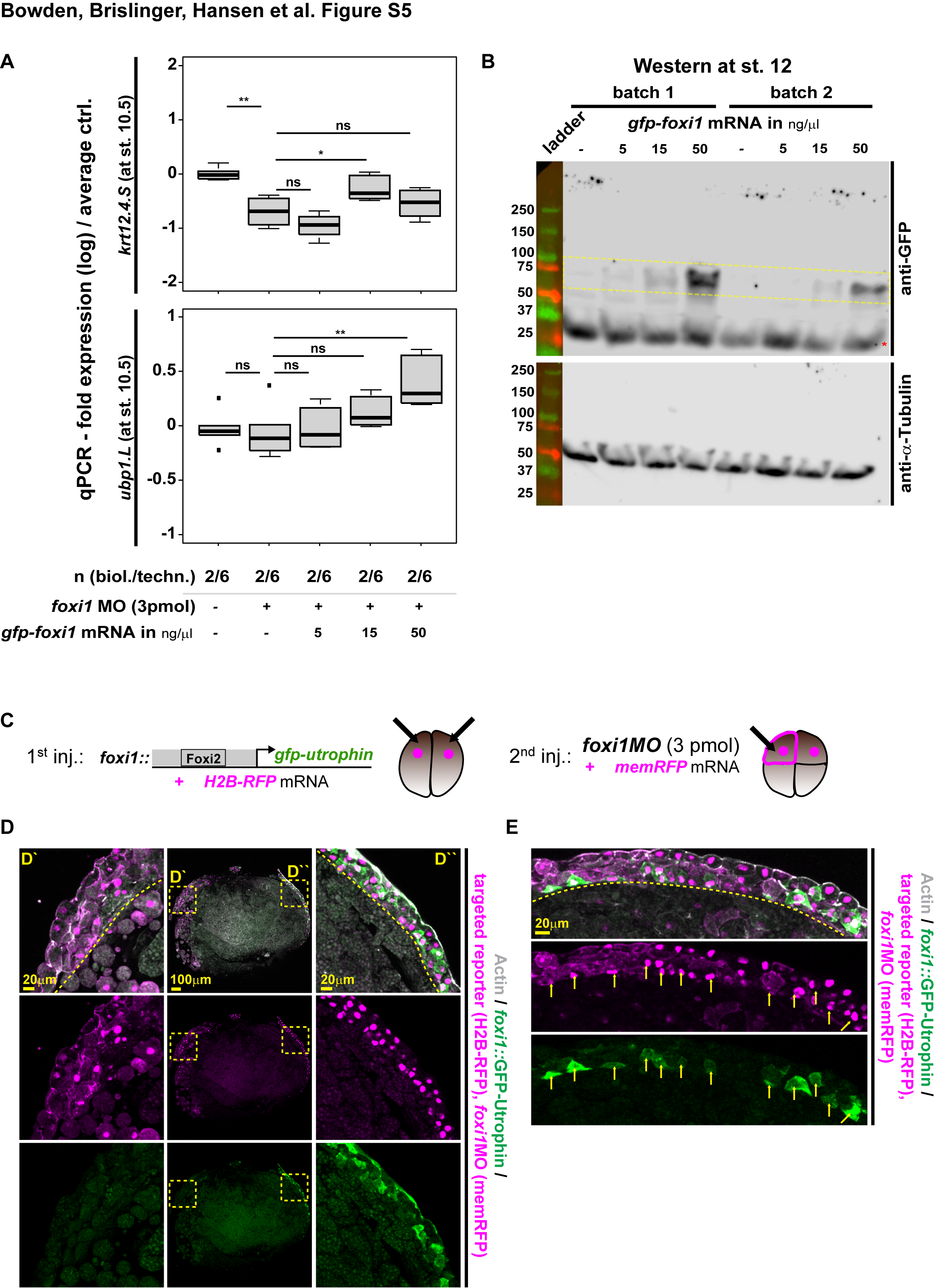

Supplement: S5 Fig — (A) qPCR on pooled uninjected control organoids and after foxi1 MO (3 pmol) with or without co-injected gfp-foxi1 at 5, 15, or 50 ng/μl. The epidermal competence gene krt12.4.S and the definitive ISC marker ubp1 show differential dose-dependent reactions to foxi1 manipulations. ANOVA (Tukey HSD corrected): p > 0.05 = ns; p < 0.01 = **. n = number of biological and technical replicates. (B) western blot analysis of GFP-Foxi1 overexpression (anti-GFP) levels in lysates from pooled whole embryos at stage 12 in uninjected controls and embryos injected with gfp-foxi1 at 5, 15, or 50 ng/μl. Two different batches (biological replicates) are shown. Predicted size of GFP-Foxi1 ca. Sixty-eight kDa, specific bands are indicated by yellow box, unspecific band indicated by red asterisk. Anti-Tubulin is used as loading control. (C–E) IF analysis of bisected st. 19 embryos injected with foxi1::gfp-utrophin (n = 9 embryos) reporter (green) into both blastomeres at 2-cell stage (identified by nuclear RFP expression; H2B-RFP, magenta), followed by injection of foxi1 MO (3 pmol) into one ventral blastomere at 4-cell stage (identified by membrane RFP expression; memRFP, magenta). Embryos were stained for F-actin (Actin, cell borders and morphology, gray). (D) Comparison of foxi1 MO targeted (left side of section) and non-targeted (right side of section) cells revealed reduced reporter activity (diminished GFP signal) after foxi1 knockdown. (D′ and D˝) show magnified areas indicated by dashed yellow boxes in overview panels. (E) Magnification of an epidermal area where morphant- and non-morphant cells mixed shows GFP signal (yellow arrows) in reporter-only targeted cells, but reduced signal in MO-targeted cells. Data used for panel (A): S1 Data. (TIF) [file pbio.3003583.s005.tif]

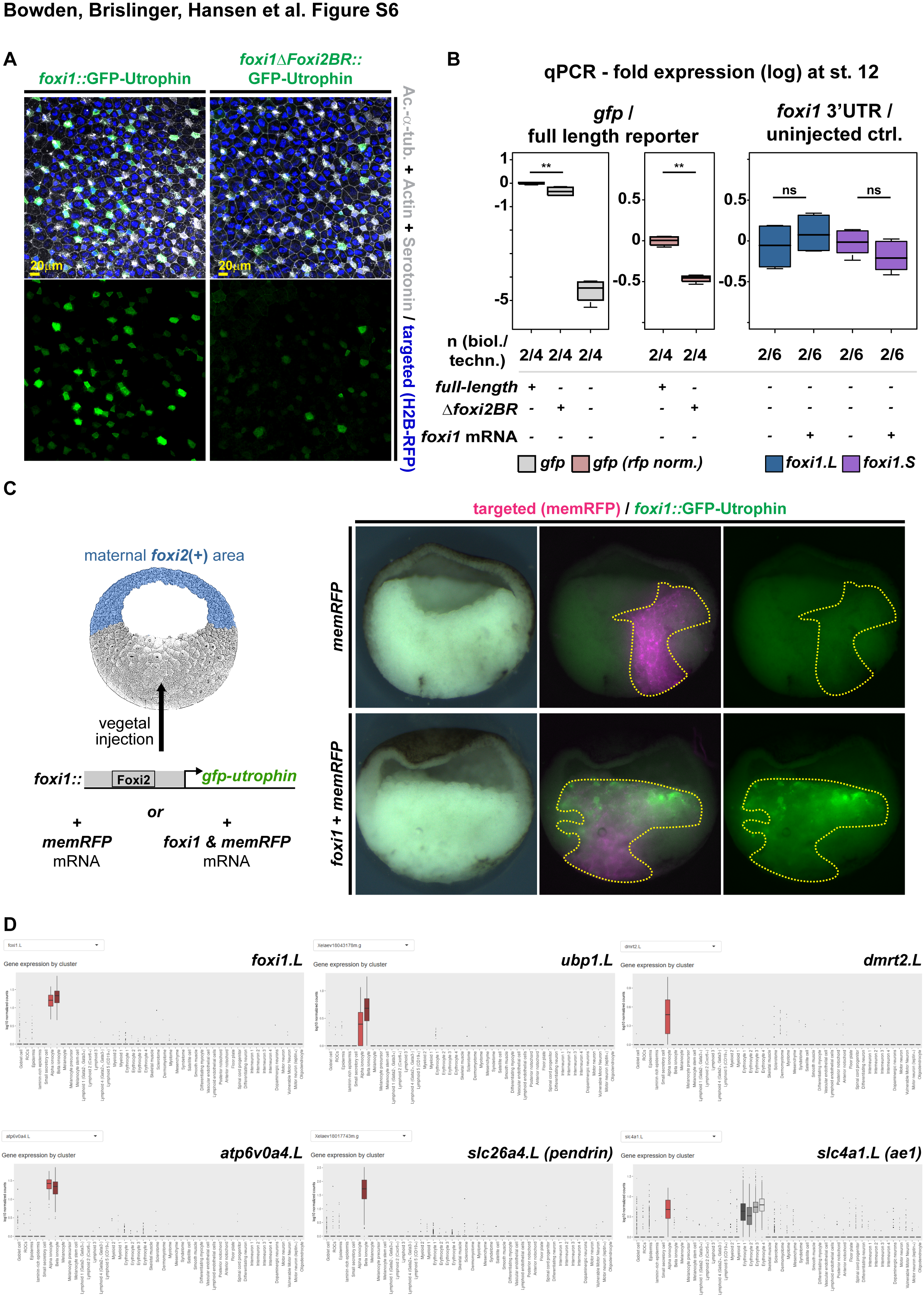

Supplement: S6 Fig — (A) IF analysis of embryos injected with foxi1::gfp-utrophin (n = 12 embryos) or foxi1ΔFoxi2BR::gfp-utrophin (n = 12 embryos) reporters (green) at st. 32 stained for Acetylated-α-tubulin (Ac.-α-tub., cilia, gray), F-actin (Actin, cell borders and morphology, gray), and serotonin (SSCs, gray) at st. 32. Targeted cells were identified by nuclear RFP expression (H2B-RFP, blue). (B) qPCR on pooled uninjected control organoids and after injection of foxi1::gfp-utrophin (full-length) or foxi1ΔFoxi2BR::gfp-utrophin (Δfoxi2BR) together with memRFP for normalization (left), and on pooled uninjected control organoids and organoids injected with 100 ng/μl of foxi1 mRNA (right). Gray box-plots = gfp expression relative to full-length reporter gfp expression; red box-plots = gfp expression normalized by rfp expression; blue box-plots = foxi1.L 3′ UTR expression; purple box-plots = foxi1.S 3′ UTR expression. T test (2-tail, paired): p > 0.05 = ns; p < 0.05 = *; p < 0.01 = **. n = number of biological and technical replicates. (C) Brightfield and epifluorescence images of hemisected st. 11 gastrula embryos injected vegetally with foxi1::gfp-utrophin (green), membrane RFP (memRFP; magenta) as control (memRFP) or with additional co-injection of foxi1 mRNA (foxi1 + memRFP). Right panels show false-color of GFP fluorescence intensity. Induction was scored as positive when GFP was detected in areas below the equator (mesendoderm). Ctrl. n = 7 induced, 26 non-induced; foxi1 mRNA = 26 induced, 11 non-induced. Embryos are shown dorsal to the left and animal up. (D) Boxplots of ISC gene expression from scRNA-seq data published in Aztekin and colleagues, 2019. Visualization was generated using the published online tool: marionilab.cruk.cam.ac.uk/XenopusRegeneration. Data used for panel (B): S1 Data. (TIF) [file pbio.3003583.s006.tif]

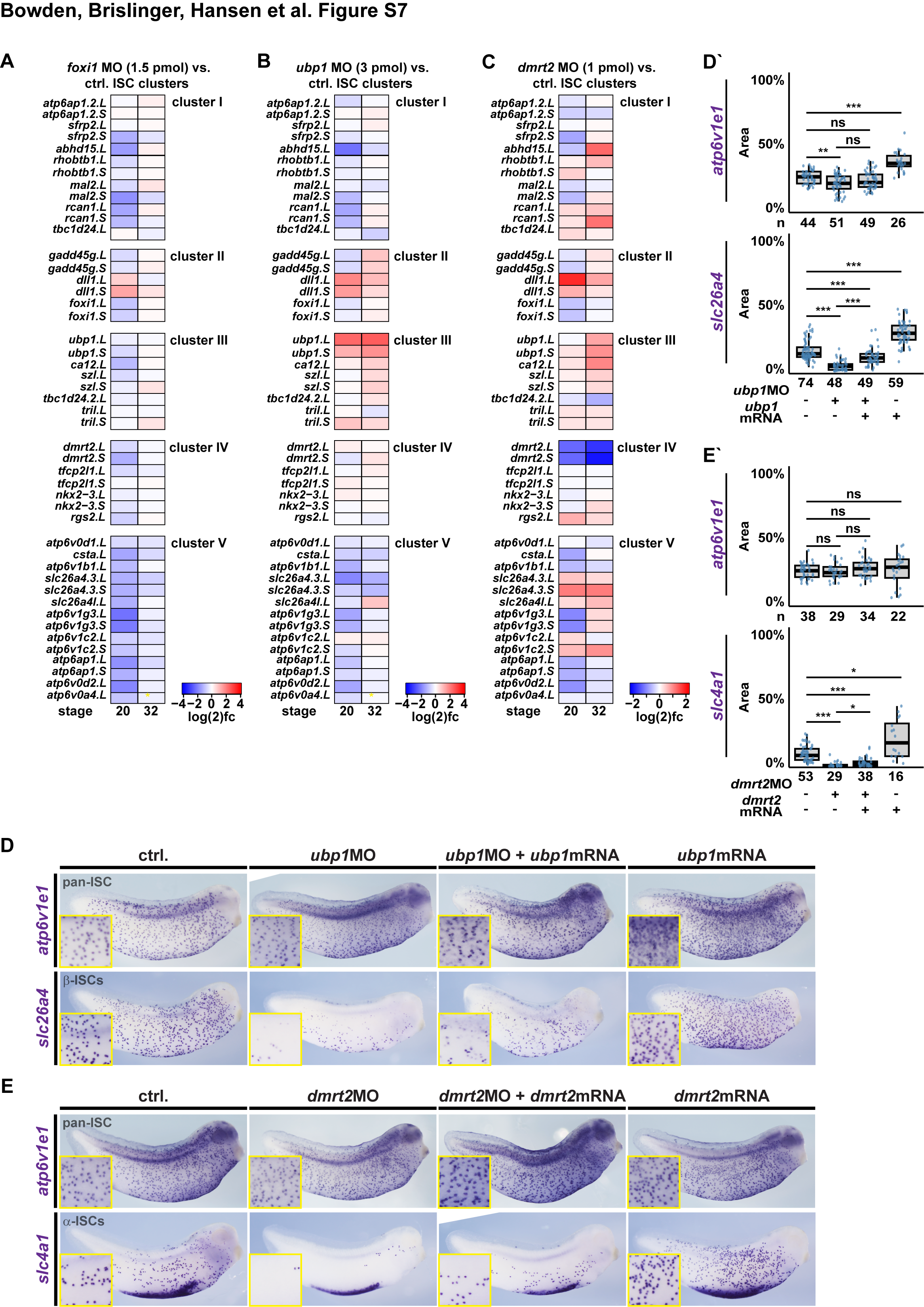

Supplement: S7 Fig — (A–C) Effects of Foxi1 (foxi1 MO, 1.5 pmol; A), Ubp1 (ubp1 MO, 3 pmol; B), or Dmrt2 (dmrt2 MO, 1 pmol; C) knockdown on core ISC gene expression stages 20 and 32. RNA-seq on mucociliary organoids. Heatmaps depict log2-fold change values derived from DEseq2. (D,E) Analysis of effects by WMISH at st. 29–32 against atp6v1e1 and foxi1 (pan-ISC markers), ubp1 and slc25a4/pendrin (β-ISC markets), and dmrt2 and slc4a1/ae1 (α-ISC markers) after Ubp1 (ubp1 MO, 3 pmol) or Dmrt2 (dmrt2 MO, 1 pmol) knockdown, rescue and overexpression (by mRNA injections: 50 ng/µl ubp1; 25–50 ng/µl dmrt2). Representative images and quantification of results are depicted. n = number of embryos analyzed per condition. Wilcoxon Rank Sum test: p > 0.05 = ns; p < 0.05 = *; p < 0.01 = **; p < 0.001 = ***. Data used for panels (A), (B), (C), (D′), and (E′): S1 Data. (TIF) [file pbio.3003583.s007.tif]

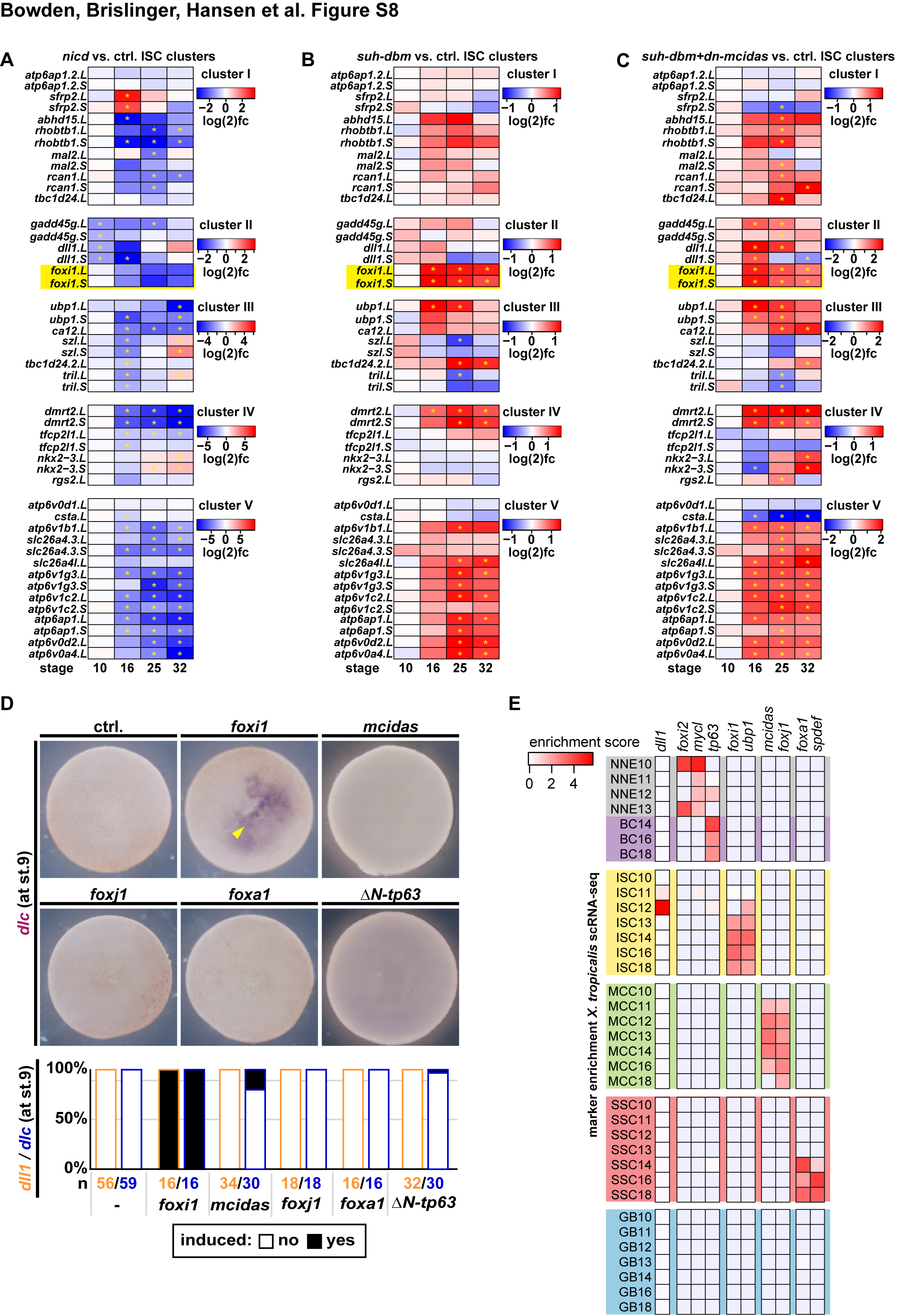

Supplement: S8 Fig — (A–C) Effects of Notch gain (nicd; A), Notch loss (suh-dbm; B), and Notch and MCC loss (suh-dbm + dn-mcidas; C) on core ISC gene expression in key developmental stages (st. 10, 16, 25, 32). Heatmaps depict log2-fold change values derived from DEseq2. Asterisks indicate statistical significant (adj-p value < 0.05) changes. (D) Representative images of st. 9 control (ctrl.) and manipulated embryos (animal views) after mRNA overexpression of transcription factors to test premature induction of dlc. Quantification of results and effects on dll1 (yellow) and dlc (blue) graphs. Embryos were scored as induced or non-induced expression. Related to Fig 5A. (E) Heatmap of mucociliary marker gene enrichment during differentiation in lineages from scRNA-seq data published in Briggs and colleagues (2018). Values were derived using the published online tool: kleintools.hms.harvard.edu/tools/currentDatasetsList_xenopus_v2.html. NNE, non-neural ectodermal precursors; BC, basal cells; ISC, ionocytes; MCC, multiciliated cells; SSC, small secretory cells; GB, outer-layer goblet cells. Data used for panels (A), (B), (C), (D), and (E): S1 Data. (TIF) [file pbio.3003583.s008.tif]

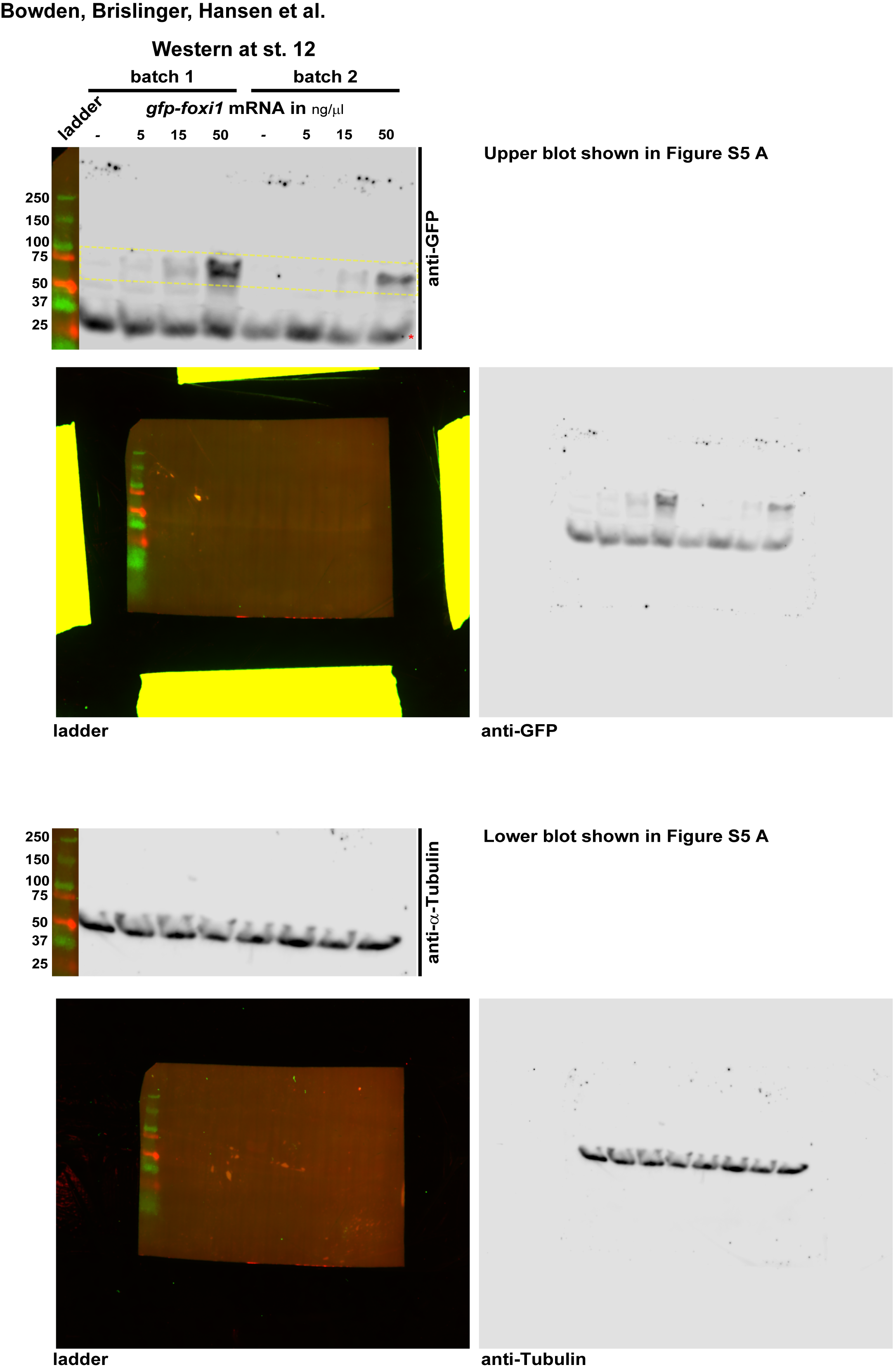

Supplement: S1 Raw Images — (TIF) [file pbio.3003583.s009.tif]

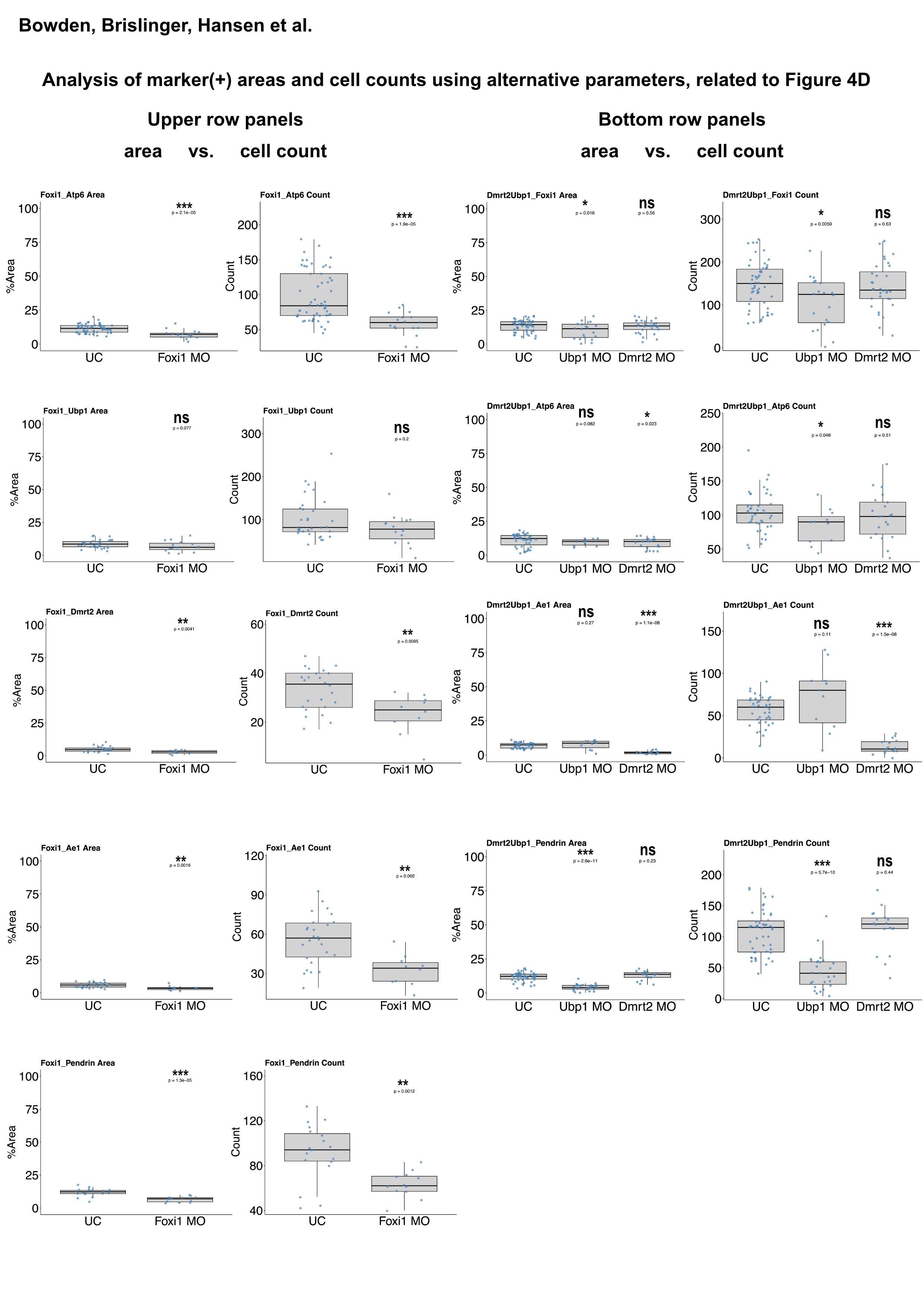

Supplement: S5 Data — (TIF) [file pbio.3003583.s014.tif]
