# Supplementary material for: Foxi1 regulates multipotent mucociliary progenitors and ionocyte specification through transcriptional and epigenetic mechanisms
Source: PLoS Biol. 2026 Jan 5;24(1):e3003583. doi: 10.1371/journal.pbio.3003583 (PMC12768278; doi:10.1371/journal.pbio.3003583)
Supplement: S4 Data — (PDF) [file pbio.3003583.s013.pdf]

Homer Known Motif Enrichment Results (MOTIFS_Lost_Enriched_MCE)

Motifs enriched in lost MCE peaks (background = lost non-MCE peaks)

Homer *de novo* Motif Results
Gene Ontology Enrichment Results
Known Motif Enrichment Results (txt file)
Total Target Sequences = 2528, Total Background Sequences = 165390

Rank	Motif	Name	P-value	log P-pvalue	q-value (Benjamini)	# Target Sequences with Motif	% of Targets Sequences with Motif	# Background Sequences with Motif	% of Background Sequences with Motif	Motif File	SVG
1	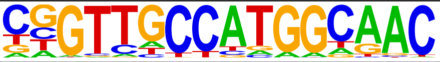	RFX(HTH)/K562-RFX3-ChIP-Seq(SRA012198)/Homer	1e-21	-5.003e+01	0.0000	90.0	3.56%	1736.6	1.05%	motif file (matrix)	svg
2	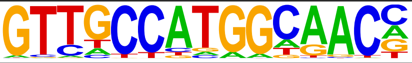	Rfx2(HTH)/LoVo-RFX2-ChIP-Seq(GSE49402)/Homer	1e-20	-4.798e+01	0.0000	98.0	3.87%	2073.0	1.25%	motif file (matrix)	svg
3	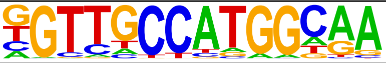	Rfx1(HTH)/NPC-H3K4me1-ChIP-Seq(GSE16256)/Homer	1e-13	-3.209e+01	0.0000	128.0	5.06%	3953.6	2.39%	motif file (matrix)	svg
4	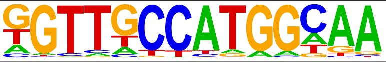	X-box(HTH)/NPC-H3K4me1-ChIP-Seq(GSE16256)/Homer	1e-13	-3.136e+01	0.0000	89.0	3.52%	2328.2	1.41%	motif file (matrix)	svg
5	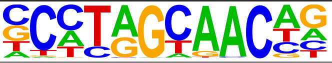	Rfx5(HTH)/GM12878-Rfx5-ChIP-Seq(GSE31477)/Homer	1e-6	-1.592e+01	0.0000	198.0	7.82%	8851.0	5.35%	motif file (matrix)	svg
6	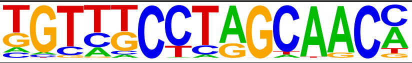	Rfx6(HTH)/Min6b1-Rfx6.HA-ChIP-Seq(GSE62844)/Homer	1e-6	-1.437e+01	0.0001	506.0	19.99%	26973.1	16.30%	motif file (matrix)	svg
7	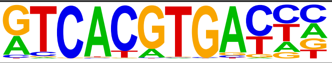	TFE3(bHLH)/MEF-TFE3-ChIP-Seq(GSE75757)/Homer	1e-4	-1.086e+01	0.0028	150.0	5.93%	6911.7	4.18%	motif file (matrix)	svg
8	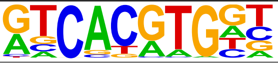	Usf2(bHLH)/C2C12-Usf2-ChIP-Seq(GSE36030)/Homer	1e-4	-1.046e+01	0.0036	232.0	9.17%	11614.6	7.02%	motif file (matrix)	svg
9	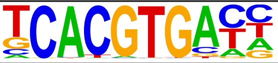	Cbf1(bHLH)/Yeast-Cbf1-ChIP-Seq(GSE29506)/Homer	1e-4	-1.009e+01	0.0046	210.0	8.30%	10416.9	6.30%	motif file (matrix)	svg
10	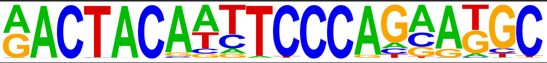	GFY-Staf(?,Zf)/Promoter/Homer	1e-4	-9.802e+00	0.0056	64.0	2.53%	2470.1	1.49%	motif file (matrix)	svg
11	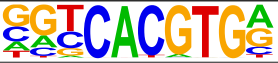	USF1(bHLH)/GM12878-Usf1-ChIP-Seq(GSE32465)/Homer	1e-4	-9.406e+00	0.0075	269.0	10.63%	13987.2	8.46%	motif file (matrix)	svg
12	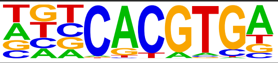	SPCH(bHLH)/Seedling-SPCH-ChIP-Seq(GSE57497)/Homer	1e-3	-7.329e+00	0.0550	459.0	18.14%	26052.3	15.75%	motif file (matrix)	svg
13	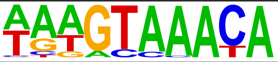	FOXA1(Forkhead)/LNCAP-FOXA1-ChIP-Seq(GSE27824)/Homer	1e-3	-7.172e+00	0.0594	673.0	26.59%	39466.5	23.86%	motif file (matrix)	svg
14	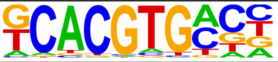	bHLHE41(bHLH)/proB-Bhlhe41-ChIP-Seq(GSE93764)/Homer	1e-3	-7.064e+00	0.0615	470.0	18.57%	26823.9	16.21%	motif file (matrix)	svg
15	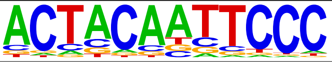	GFY(?)/Promoter/Homer	1e-2	-6.845e+00	0.0714	63.0	2.49%	2717.5	1.64%	motif file (matrix)	svg
16	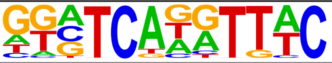	Six1(Homeobox)/Myoblast-Six1-ChIP-Chip(GSE20150)/Homer	1e-2	-6.705e+00	0.0770	173.0	6.84%	8945.1	5.41%	motif file (matrix)	svg
17	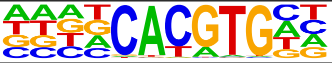	BIM2(bHLH)/col-BIM2-DAP-Seq(GSE60143)/Homer	1e-2	-6.495e+00	0.0894	465.0	18.37%	26716.8	16.15%	motif file (matrix)	svg
18	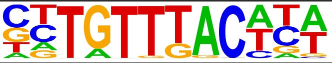	Foxa2(Forkhead)/Liver-Foxa2-ChIP-Seq(GSE25694)/Homer	1e-2	-6.447e+00	0.0894	439.0	17.34%	25122.4	15.19%	motif file (matrix)	svg
19	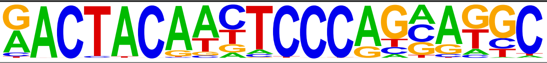	Ronin(THAP)/ES-Thap11-ChIP-Seq(GSE51522)/Homer	1e-2	-6.394e+00	0.0894	46.0	1.82%	1876.3	1.13%	motif file (matrix)	svg
20	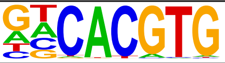	CLOCK(bHLH)/Liver-Clock-ChIP-Seq(GSE39860)/Homer	1e-2	-6.104e+00	0.1123	276.0	10.90%	15242.9	9.21%	motif file (matrix)	svg
21	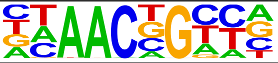	BMYB(HTH)/Hela-BMYB-ChIP-Seq(GSE27030)/Homer	1e-2	-5.802e+00	0.1448	784.0	30.98%	47107.1	28.48%	motif file (matrix)	svg
22	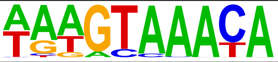	FOXA1(Forkhead)/MCF7-FOXA1-ChIP-Seq(GSE26831)/Homer	1e-2	-5.754e+00	0.1450	569.0	22.48%	33510.0	20.26%	motif file (matrix)	svg
23	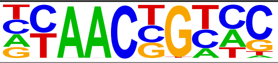	MYB77(MYB)/col-MYB77-DAP-Seq(GSE60143)/Homer	1e-2	-5.635e+00	0.1563	692.0	27.34%	41335.7	24.99%	motif file (matrix)	svg
24	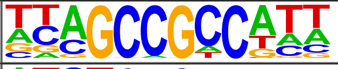	ERF13(AP2EREBP)/colamp-ERF13-DAP-Seq(GSE60143)/Homer	1e-2	-5.624e+00	0.1563	350.0	13.83%	19919.5	12.04%	motif file (matrix)	svg
25	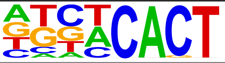	AZF1(C2H2)/colamp-AZF1-DAP-Seq(GSE60143)/Homer	1e-2	-5.611e+00	0.1563	1811.0	71.55%	114288.1	69.09%	motif file (matrix)	svg
26	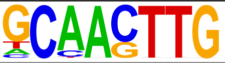	bHLH130(bHLH)/col-bHLH130-DAP-Seq(GSE60143)/Homer	1e-2	-5.436e+00	0.1687	284.0	11.22%	15935.9	9.63%	motif file (matrix)	svg
27	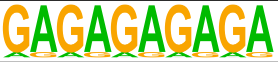	SeqBias: GA-repeat	1e-2	-5.303e+00	0.1855	1767.0	69.81%	111512.0	67.41%	motif file (matrix)	svg
28	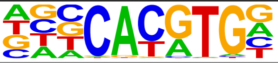	PIF4(bHLH)/Seedling-PIF4-ChIP-Seq(GSE35315)/Homer	1e-2	-5.191e+00	0.2000	573.0	22.64%	34012.8	20.56%	motif file (matrix)	svg
29	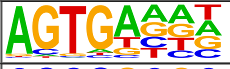	At5g04390(C2H2)/col200-At5g04390-DAP-Seq(GSE60143)/Homer	1e-2	-5.103e+00	0.2109	1944.0	76.81%	123472.1	74.64%	motif file (matrix)	svg
30	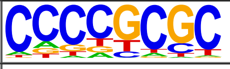	SUT1?/SacCer-Promoters/Homer	1e-2	-5.099e+00	0.2109	1671.0	66.02%	105238.3	63.61%	motif file (matrix)	svg
31		IBL1(bHLH)/Seedling-IBL1-	1e-2	-5.032e+00	0.2117	587.0	23.19%	34970.5	21.14%	motif	svg

	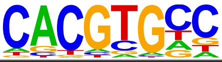	ChIP-Seq(GSE51120)/Homer								file (matrix)	
32	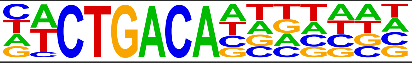	GRF9(GRF)/colamp-GRF9-DAP-Seq(GSE60143)/Homer	1e-2	-4.915e+00	0.2306	448.0	17.70%	26280.9	15.89%	motif file (matrix)	svg
33	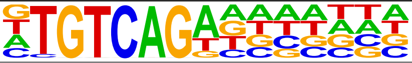	AtGRF6(GRF)/col-AtGRF6-DAP-Seq(GSE60143)/Homer	1e-2	-4.914e+00	0.2306	530.0	20.94%	31429.6	19.00%	motif file (matrix)	svg
34	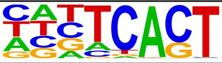	STZ(C2H2)/colamp-STZ-DAP-Seq(GSE60143)/Homer	1e-2	-4.789e+00	0.2461	1964.0	77.60%	124980.2	75.55%	motif file (matrix)	svg
35	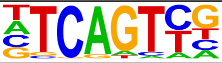	Initiator/Drosophila-Promoters/Homer	1e-2	-4.782e+00	0.2461	919.0	36.31%	56292.4	34.03%	motif file (matrix)	svg
36	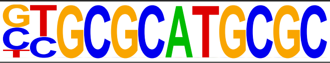	NRF(NRF)/Promoter/Homer	1e-2	-4.754e+00	0.2461	113.0	4.46%	5860.2	3.54%	motif file (matrix)	svg
37	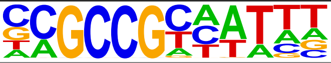	RRTF1(AP2EREBP)/colamp-RRTF1-DAP-Seq(GSE60143)/Homer	1e-2	-4.693e+00	0.2491	96.0	3.79%	4884.6	2.95%	motif file (matrix)	svg
38	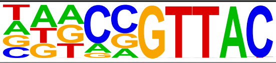	MYB98(MYB)/col-MYB98-DAP-Seq(GSE60143)/Homer	1e-2	-4.687e+00	0.2491	206.0	8.14%	11425.8	6.91%	motif file (matrix)	svg
39	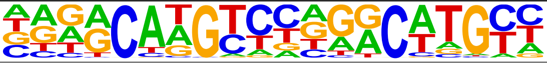	p73(p53)/Trachea-p73-ChIP-Seq(PRJNA310161)/Homer	1e-2	-4.683e+00	0.2491	24.0	0.95%	916.2	0.55%	motif file (matrix)	svg
