# Supplementary material for: Foxi1 regulates multipotent mucociliary progenitors and ionocyte specification through transcriptional and epigenetic mechanisms
Source: PLoS Biol. 2026 Jan 5;24(1):e3003583. doi: 10.1371/journal.pbio.3003583 (PMC12768278; doi:10.1371/journal.pbio.3003583)
Supplement: S3 Data — (PDF) [file pbio.3003583.s012.pdf]

Homer Known Motif Enrichment Results (allNoMT_condition1_txt_motif)

Lost Peaks after 3pmol Foxi1MO

Homer *de novo* Motif Results
Gene Ontology Enrichment Results
Known Motif Enrichment Results (txt file)
Total Target Sequences = 168953, Total Background Sequences = 127876

Rank	Motif	Name	P-value	log P-pvalue	q-value (Benjamini)	# Target Sequences with Motif	% of Targets Sequences with Motif	# Background Sequences with Motif	% of Background Sequences with Motif	Motif File	SVG
1		TCP7(TCP)/col-TCP7-DAP-Seq(GSE60143)/Homer	1e-89	-2.058e+02	0.0000	7586.0	4.49%	4539.6	3.55%	motif file (matrix)	svg
2		AP-2alpha(AP2)/Hela-AP2alpha-ChIP-Seq(GSE31477)/Homer	1e-85	-1.962e+02	0.0000	8599.0	5.09%	5255.0	4.11%	motif file (matrix)	svg
3		TCP16(TCP)/colamp-TCP16-DAP-Seq(GSE60143)/Homer	1e-74	-1.715e+02	0.0000	7622.0	4.51%	4663.6	3.64%	motif file (matrix)	svg
4		AP-2gamma(AP2)/MCF7-TFAP2C-ChIP-Seq(GSE21234)/Homer	1e-73	-1.701e+02	0.0000	12051.0	7.13%	7733.5	6.04%	motif file (matrix)	svg
5		TBP3(MYBrelated)/col-TBP3-DAP-Seq(GSE60143)/Homer	1e-67	-1.561e+02	0.0000	14171.0	8.38%	9285.8	7.26%	motif file (matrix)	svg
6		SeqBias: TA-repeat	1e-67	-1.544e+02	0.0000	70146.0	41.49%	50449.0	39.42%	motif file (matrix)	svg
7		AT1G72740(MYBrelated)/colamp-AT1G72740-DAP-Seq(GSE60143)/Homer	1e-63	-1.469e+02	0.0000	19048.0	11.27%	12809.8	10.01%	motif file (matrix)	svg
8		At5g08330(TCP)/col-At5g08330-DAP-Seq(GSE60143)/Homer	1e-60	-1.385e+02	0.0000	8880.0	5.25%	5642.6	4.41%	motif file (matrix)	svg
9		HIC1(Zf)/Treg-ZBTB29-ChIP-Seq(GSE99889)/Homer	1e-56	-1.291e+02	0.0000	27864.0	16.48%	19305.3	15.08%	motif file (matrix)	svg
10		At1g69690(TCP)/colamp-At1g69690-DAP-Seq(GSE60143)/Homer	1e-55	-1.277e+02	0.0000	4428.0	2.62%	2627.3	2.05%	motif file (matrix)	svg
11		SRS7(SRS)/colamp-SRS7-DAP-Seq(GSE60143)/Homer	1e-53	-1.237e+02	0.0000	61655.0	36.47%	44365.4	34.67%	motif file (matrix)	svg
12		LBD18(LOBAS2)/colamp-LBD18-DAP-Seq(GSE60143)/Homer	1e-52	-1.211e+02	0.0000	47016.0	27.81%	33478.9	26.16%	motif file (matrix)	svg
13		MYB83(MYB)/colamp-MYB83-DAP-Seq(GSE60143)/Homer	1e-50	-1.159e+02	0.0000	27050.0	16.00%	18804.9	14.69%	motif file (matrix)	svg
14		GATA15(C2C2gata)/col-GATA15-DAP-Seq(GSE60143)/Homer	1e-50	-1.153e+02	0.0000	23026.0	13.62%	15875.9	12.40%	motif file (matrix)	svg
15		At3g24120(G2like)/col-At3g24120-DAP-Seq(GSE60143)/Homer	1e-47	-1.096e+02	0.0000	58493.0	34.60%	42137.5	32.93%	motif file (matrix)	svg
16		CCA(Myb)/Arabidopsis-CCA.GFP-ChIP-Seq(GSE70533)/Homer	1e-47	-1.088e+02	0.0000	15358.0	9.08%	10371.6	8.10%	motif file (matrix)	svg
17		ZNF692(Zf)/HEK293-ZNF692.GFP-ChIP-Seq(GSE58341)/Homer	1e-47	-1.084e+02	0.0000	3289.0	1.95%	1917.5	1.50%	motif file (matrix)	svg
18		At1g72010(TCP)/colamp-At1g72010-DAP-Seq(GSE60143)/Homer	1e-45	-1.039e+02	0.0000	7234.0	4.28%	4629.6	3.62%	motif file (matrix)	svg
19		TCP20(TCP)/col-TCP20-DAP-Seq(GSE60143)/Homer	1e-41	-9.587e+01	0.0000	5794.0	3.43%	3659.2	2.86%	motif file (matrix)	svg
20		MYB107(MYB)/col-MYB107-DAP-Seq(GSE60143)/Homer	1e-40	-9.438e+01	0.0000	23926.0	14.15%	16681.1	13.03%	motif file (matrix)	svg
21		RBFox2(?)/Heart-RBFox2-CLIP-Seq(GSE57926)/Homer	1e-40	-9.436e+01	0.0000	34225.0	20.24%	24248.5	18.95%	motif file (matrix)	svg
22		Pitx1(Homeobox)/Chicken-Pitx1-ChIP-Seq(GSE38910)/Homer	1e-40	-9.309e+01	0.0000	76155.0	45.04%	55577.5	43.43%	motif file (matrix)	svg
23		Zac1(Zf)/Neuro2A-Plagl1-ChIP-Seq(GSE75942)/Homer	1e-39	-9.193e+01	0.0000	39391.0	23.30%	28091.2	21.95%	motif file (matrix)	svg
24		MYB61(MYB)/colamp-MYB61-DAP-Seq(GSE60143)/Homer	1e-39	-9.037e+01	0.0000	22618.0	13.38%	15756.9	12.31%	motif file (matrix)	svg
25		LHY(Myb)/Seedling-LHY-ChIP-Seq(GSE52175)/Homer	1e-39	-8.992e+01	0.0000	19554.0	11.57%	13526.8	10.57%	motif file (matrix)	svg
26		ATHB40(HB)/col-ATHB40-DAP-Seq(GSE60143)/Homer	1e-38	-8.963e+01	0.0000	18124.0	10.72%	12489.4	9.76%	motif file (matrix)	svg
27		THRb(NR)/Liver-NR1A2-ChIP-Seq(GSE52613)/Homer	1e-38	-8.786e+01	0.0000	54116.0	32.01%	39092.5	30.55%	motif file (matrix)	svg
28		RARa(NR)/K562-RARa-ChIP-Seq(Encode)/Homer	1e-37	-8.669e+01	0.0000	38880.0	23.00%	27763.2	21.69%	motif file (matrix)	svg
29		PABPC1(?)/MEL-PABC1-CLIP-Seq(GSE69755)/Homer	1e-36	-8.454e+01	0.0000	42890.0	25.37%	30761.4	24.04%	motif file (matrix)	svg
30		p63(p53)/Keratinocyte-p63-ChIP-Seq(GSE17611)/Homer	1e-35	-8.065e+01	0.0000	3960.0	2.34%	2449.4	1.91%	motif file (matrix)	svg
31		Tgif1(Homeobox)/mES-Tgif1-ChIP-Seq(GSE55404)/Homer	1e-35	-8.064e+01	0.0000	52610.0	31.12%	38047.6	29.73%	motif file (matrix)	svg
32		RXR(NR),DR1/3T3L1-RXR-ChIP-Seq(GSE13511)/Homer	1e-34	-8.025e+01	0.0000	13297.0	7.86%	9059.8	7.08%	motif file (matrix)	svg
33		RUNX-AML(Runt)/CD4+-PolII-ChIP-Seq(Barski_et_al.)/Homer	1e-34	-7.915e+01	0.0000	9043.0	5.35%	6018.0	4.70%	motif file (matrix)	svg
34		Smad4(MAD)/ESC-SMAD4-ChIP-Seq(GSE29422)/Homer	1e-33	-7.770e+01	0.0000	21352.0	12.63%	14930.0	11.67%	motif file (matrix)	svg
35		MYB67(MYB)/col-MYB67-DAP-Seq(GSE60143)/Homer	1e-33	-7.751e+01	0.0000	14726.0	8.71%	10109.1	7.90%	motif file (matrix)	svg
36		MYB51(MYB)/col-MYB51-DAP-Seq(GSE60143)/Homer	1e-32	-7.589e+01	0.0000	11190.0	6.62%	7570.5	5.92%	motif file (matrix)	svg
37		PRDM15(Zf)/ESC-Prdm15-ChIP-Seq(GSE73694)/Homer	1e-32	-7.398e+01	0.0000	11818.0	6.99%	8033.4	6.28%	motif file (matrix)	svg
38		MYB3(MYB)/Arabidopsis-MYB3-ChIP-Seq(GSE80564)/Homer	1e-31	-7.344e+01	0.0000	24505.0	14.49%	17277.0	13.50%	motif file (matrix)	svg
39		ATY19(MYB)/col-ATY19-DAP-Seq(GSE60143)/Homer	1e-31	-7.293e+01	0.0000	10505.0	6.21%	7096.9	5.55%	motif file (matrix)	svg
40		MYB99(MYB)/colamp-MYB99-DAP-Seq(GSE60143)/Homer	1e-31	-7.267e+01	0.0000	12847.0	7.60%	8783.5	6.86%	motif file (matrix)	svg
41		AT4G26030(C2H2)/col-AT4G26030-DAP-Seq(GSE60143)/Homer	1e-31	-7.228e+01	0.0000	14857.0	8.79%	10240.1	8.00%	motif file (matrix)	svg
42		COUP-TFII(NR)/Artia-Nr2f2-ChIP-Seq(GSE46497)/Homer	1e-30	-6.934e+01	0.0000	23531.0	13.92%	16598.9	12.97%	motif file (matrix)	svg
43		EBF2(EBF)/BrownAdipose-EBF2-ChIP-Seq(GSE97114)/Homer	1e-29	-6.757e+01	0.0000	8378.0	4.96%	5605.8	4.38%	motif file (matrix)	svg
44		Eomes(T-box)/H9-Eomes-ChIP-Seq(GSE26097)/Homer	1e-28	-6.633e+01	0.0000	34602.0	20.47%	24802.0	19.38%	motif file (matrix)	svg
45		Tbr1(T-box)/Cortex-Tbr1-ChIP-Seq(GSE71384)/Homer	1e-28	-6.625e+01	0.0000	22438.0	13.27%	15823.8	12.36%	motif file (matrix)	svg
46		At2g45680(TCP)/colamp-At2g45680-DAP-Seq(GSE60143)/Homer	1e-28	-6.476e+01	0.0000	1086.0	0.64%	576.7	0.45%	motif file (matrix)	svg
47		Unknown2/Drosophila-Promoters/Homer	1e-27	-6.441e+01	0.0000	20742.0	12.27%	14595.2	11.40%	motif file (matrix)	svg
48		MYB55(MYB)/colamp-MYB55-DAP-Seq(GSE60143)/Homer	1e-27	-6.373e+01	0.0000	17216.0	10.18%	12021.4	9.39%	motif file (matrix)	svg
49		WRKY18(WRKY)/col-WRKY18-DAP-Seq(GSE60143)/Homer	1e-27	-6.299e+01	0.0000	20898.0	12.36%	14722.2	11.50%	motif file (matrix)	svg
50		AMYB(HTH)/Testes-AMYB-ChIP-Seq(GSE44588)/Homer	1e-27	-6.291e+01	0.0000	29353.0	17.36%	20951.6	16.37%	motif file (matrix)	svg
51		ATHB13(Homeobox)/col-ATHB13-DAP-Seq(GSE60143)/Homer	1e-27	-6.262e+01	0.0000	24777.0	14.65%	17577.2	13.73%	motif file (matrix)	svg
52		LMI1(HB)/colamp-LMI1-DAP-Seq(GSE60143)/Homer	1e-26	-6.173e+01	0.0000	15786.0	9.34%	10994.2	8.59%	motif file (matrix)	svg
53		Adof1(C2C2dof)/col-Adof1-DAP-Seq(GSE60143)/Homer	1e-26	-6.083e+01	0.0000	58892.0	34.83%	42996.3	33.60%	motif file (matrix)	svg
54		LBD19(LOBAS2)/colamp-LBD19-DAP-Seq(GSE60143)/Homer	1e-26	-6.015e+01	0.0000	33759.0	19.97%	24244.8	18.94%	motif file (matrix)	svg
55		EAR2(NR)/K562-NR2F6-ChIP-Seq(Encode)/Homer	1e-26	-6.000e+01	0.0000	16699.0	9.88%	11674.0	9.12%	motif file (matrix)	svg
56		PPARE(NR),DR1/3T3L1-Pparg-ChIP-Seq(GSE13511)/Homer	1e-25	-5.798e+01	0.0000	11796.0	6.98%	8124.0	6.35%	motif file (matrix)	svg
57		CRX(Homeobox)/Retina-Crx-ChIP-Seq(GSE20012)/Homer	1e-25	-5.790e+01	0.0000	40986.0	24.24%	29644.5	23.16%	motif file (matrix)	svg
58		Tbx6(T-box)/ESC-Tbx6-ChIP-Seq(GSE93524)/Homer	1e-25	-5.765e+01	0.0000	17639.0	10.43%	12381.0	9.67%	motif file (matrix)	svg
59		At3g12730(G2like)/colamp-At3g12730-DAP-Seq(GSE60143)/Homer	1e-24	-5.737e+01	0.0000	25818.0	15.27%	18397.1	14.37%	motif file (matrix)	svg
60		MYB58(MYB)/colamp-MYB58-DAP-Seq(GSE60143)/Homer	1e-24	-5.654e+01	0.0000	14327.0	8.47%	9972.0	7.79%	motif file (matrix)	svg
61		KAN2(G2like)/colamp-KAN2-DAP-Seq(GSE60143)/Homer	1e-24	-5.647e+01	0.0000	26102.0	15.44%	18616.7	14.55%	motif file (matrix)	svg
62		MYB49(MYB)/col-MYB49-DAP-Seq(GSE60143)/Homer	1e-24	-5.645e+01	0.0000	16936.0	10.02%	11878.0	9.28%	motif file (matrix)	svg
63		MYB93(MYB)/colamp-MYB93-DAP-Seq(GSE60143)/Homer	1e-23	-5.460e+01	0.0000	16843.0	9.96%	11825.6	9.24%	motif file (matrix)	svg
64		PTF1(TCP)/colamp-PTF1-DAP-Seq(GSE60143)/Homer	1e-23	-5.439e+01	0.0000	1185.0	0.70%	659.5	0.52%	motif file (matrix)	svg
65		ATHB15(HB)/col-ATHB15-DAP-Seq(GSE60143)/Homer	1e-23	-5.429e+01	0.0000	5084.0	3.01%	3335.2	2.61%	motif file (matrix)	svg
66		At3g04030(G2like)/col-At3g04030-DAP-Seq(GSE60143)/Homer	1e-23	-5.404e+01	0.0000	15730.0	9.30%	11016.7	8.61%	motif file (matrix)	svg
67		At4g01280(MYBrelated)/colamp-At4g01280-DAP-Seq(GSE60143)/Homer	1e-23	-5.371e+01	0.0000	11076.0	6.55%	7632.6	5.96%	motif file (matrix)	svg
68		RUNX1(Runt)/Jurkat-RUNX1-ChIP-Seq(GSE29180)/Homer	1e-21	-4.853e+01	0.0000	13501.0	7.99%	9435.6	7.37%	motif file (matrix)	svg
69		ATHB6(Homeobox)/Arabidopsis-HB6-ChIP-Seq(GSE80564)/Homer	1e-21	-4.850e+01	0.0000	15008.0	8.88%	10536.3	8.23%	motif file (matrix)	svg
70		Tgif2(Homeobox)/mES-Tgif2-ChIP-Seq(GSE55404)/Homer	1e-20	-4.803e+01	0.0000	55771.0	32.99%	40837.1	31.91%	motif file (matrix)	svg
71		PPARa(NR),DR1/Liver-Ppara-ChIP-Seq(GSE47954)/Homer	1e-20	-4.744e+01	0.0000	12339.0	7.30%	8597.3	6.72%	motif file (matrix)	svg
72		MYB4(MYB)/col200-MYB4-DAP-Seq(GSE60143)/Homer	1e-20	-4.735e+01	0.0000	9937.0	5.88%	6853.9	5.36%	motif file (matrix)	svg
73		GATA19(C2C2gata)/colamp-GATA19-DAP-Seq(GSE60143)/Homer	1e-20	-4.686e+01	0.0000	1815.0	1.07%	1095.2	0.86%	motif file (matrix)	svg
74		PR(NR)/T47D-PR-ChIP-Seq(GSE31130)/Homer	1e-20	-4.605e+01	0.0000	35252.0	20.85%	25526.0	19.95%	motif file (matrix)	svg
75		MYB63(MYB)/col-MYB63-DAP-Seq(GSE60143)/Homer	1e-19	-4.589e+01	0.0000	6375.0	3.77%	4297.1	3.36%	motif file (matrix)	svg
76		ZNF165(Zf)/WHIM12-ZNF165-ChIP-Seq(GSE65937)/Homer	1e-19	-4.570e+01	0.0000	1421.0	0.84%	834.6	0.65%	motif file (matrix)	svg
77		KANADI1(Myb)/Seedling-KAN1-ChIP-Seq(GSE48081)/Homer	1e-19	-4.562e+01	0.0000	25547.0	15.11%	18326.0	14.32%	motif file (matrix)	svg
78		SGR5(C2H2)/colamp-SGR5-DAP-Seq(GSE60143)/Homer	1e-19	-4.462e+01	0.0000	9792.0	5.79%	6768.7	5.29%	motif file (matrix)	svg
79		GATA1(C2C2gata)/colamp-GATA1-DAP-Seq(GSE60143)/Homer	1e-19	-4.442e+01	0.0000	5746.0	3.40%	3856.5	3.01%	motif file (matrix)	svg
80		MS188(MYB)/colamp-MS188-DAP-Seq(GSE60143)/Homer	1e-19	-4.397e+01	0.0000	13531.0	8.00%	9496.5	7.42%	motif file (matrix)	svg
81		MYB92(MYB)/colamp-MYB92-DAP-Seq(GSE60143)/Homer	1e-18	-4.336e+01	0.0000	13250.0	7.84%	9296.5	7.26%	motif file (matrix)	svg
82		MYB74(MYB)/colamp-MYB74-DAP-Seq(GSE60143)/Homer	1e-18	-4.287e+01	0.0000	9414.0	5.57%	6507.3	5.08%	motif file (matrix)	svg
83		ATY13(MYB)/col-ATY13-DAP-Seq(GSE60143)/Homer	1e-18	-4.278e+01	0.0000	64204.0	37.97%	47258.5	36.93%	motif file (matrix)	svg
84		AT3G10580(MYBrelated)/colamp-AT3G10580-DAP-Seq(GSE60143)/Homer	1e-18	-4.262e+01	0.0000	6852.0	4.05%	4659.8	3.64%	motif file (matrix)	svg
85		E2A(bHLH),near_PU.1/Bcell-PU.1-ChIP-Seq(GSE21512)/Homer	1e-18	-4.241e+01	0.0000	17343.0	10.26%	12306.5	9.62%	motif file (matrix)	svg
86		NGA4(ABI3VP1)/col-NGA4-DAP-Seq(GSE60143)/Homer	1e-18	-4.234e+01	0.0000	37629.0	22.26%	27348.0	21.37%	motif file (matrix)	svg
87		CELF2(RRM)/JSL1-CELF2-CLIP-Seq(GSE71264)/Homer	1e-18	-4.211e+01	0.0000	5544.0	3.28%	3726.0	2.91%	motif file (matrix)	svg
88		ZBTB12(Zf)/HEK293-ZBTB12.GFP-ChIP-Seq(GSE58341)/Homer	1e-18	-4.189e+01	0.0000	3357.0	1.99%	2177.2	1.70%	motif file (matrix)	svg
89		GSC(Homeobox)/FrogEmbryos-GSC-ChIP-Seq(DRA000576)/Homer	1e-17	-4.121e+01	0.0000	20239.0	11.97%	14452.2	11.29%	motif file (matrix)	svg
90		SF1(NR)/H295R-Nr5a1-ChIP-Seq(GSE44220)/Homer	1e-17	-4.022e+01	0.0000	3767.0	2.23%	2473.5	1.93%	motif file (matrix)	svg
91		ATHB20(Homeobox)/colamp-ATHB20-DAP-Seq(GSE60143)/Homer	1e-17	-4.000e+01	0.0000	7583.0	4.48%	5203.3	4.07%	motif file (matrix)	svg
92		HLF(bZIP)/HSC-HLF.Flag-ChIP-Seq(GSE69817)/Homer	1e-16	-3.891e+01	0.0000	19074.0	11.28%	13618.2	10.64%	motif file (matrix)	svg
93		MYB27(MYB)/colamp-MYB27-DAP-Seq(GSE60143)/Homer	1e-16	-3.889e+01	0.0000	3204.0	1.90%	2083.2	1.63%	motif file (matrix)	svg
94		At1g25550(G2like)/colamp-At1g25550-DAP-Seq(GSE60143)/Homer	1e-16	-3.873e+01	0.0000	17317.0	10.24%	12325.5	9.63%	motif file (matrix)	svg
95		Unknown5/Drosophila-Promoters/Homer	1e-16	-3.872e+01	0.0000	8335.0	4.93%	5756.9	4.50%	motif file (matrix)	svg
96		GRF9(GRF)/colamp-GRF9-DAP-Seq(GSE60143)/Homer	1e-16	-3.848e+01	0.0000	16630.0	9.84%	11822.1	9.24%	motif file (matrix)	svg
97		Hoxc9(Homeobox)/Ainv15-Hoxc9-ChIP-Seq(GSE21812)/Homer	1e-16	-3.838e+01	0.0000	9812.0	5.80%	6831.5	5.34%	motif file (matrix)	svg
98		PBX2(Homeobox)/K562-PBX2-ChIP-Seq(Encode)/Homer	1e-16	-3.828e+01	0.0000	17924.0	10.60%	12777.2	9.98%	motif file (matrix)	svg
99		RVE1(MYBrelated)/col-RVE1-DAP-Seq(GSE60143)/Homer	1e-16	-3.812e+01	0.0000	13548.0	8.01%	9562.8	7.47%	motif file (matrix)	svg
100		Smad2(MAD)/ES-SMAD2-ChIP-Seq(GSE29422)/Homer	1e-16	-3.772e+01	0.0000	20375.0	12.05%	14592.4	11.40%	motif file (matrix)	svg
101		WRKY28(WRKY)/col-WRKY28-DAP-Seq(GSE60143)/Homer	1e-16	-3.732e+01	0.0000	16305.0	9.64%	11595.6	9.06%	motif file (matrix)	svg
102		MYB121(MYB)/col-MYB121-DAP-Seq(GSE60143)/Homer	1e-16	-3.713e+01	0.0000	5698.0	3.37%	3865.6	3.02%	motif file (matrix)	svg
103		ERE(NR),IR3/MCF7-ERa-ChIP-Seq(Unpublished)/Homer	1e-16	-3.706e+01	0.0000	2368.0	1.40%	1507.8	1.18%	motif file (matrix)	svg
104		Dorsal(RHD)/Embryo-dl-ChIP-Seq(GSE65441)/Homer	1e-15	-3.648e+01	0.0000	2629.0	1.55%	1691.0	1.32%	motif file (matrix)	svg
105		STAT4(Stat)/CD4-Stat4-ChIP-Seq(GSE22104)/Homer	1e-15	-3.609e+01	0.0000	11927.0	7.05%	8394.1	6.56%	motif file (matrix)	svg
106		EPR1(MYBrelated)/colamp-EPR1-DAP-Seq(GSE60143)/Homer	1e-15	-3.590e+01	0.0000	6873.0	4.07%	4719.3	3.69%	motif file (matrix)	svg
107		LHY1(MYBrelated)/col-LHY1-DAP-Seq(GSE60143)/Homer	1e-15	-3.590e+01	0.0000	6873.0	4.07%	4719.3	3.69%	motif file (matrix)	svg
108		BAM8(BES1)/col-BAM8-DAP-Seq(GSE60143)/Homer	1e-15	-3.589e+01	0.0000	1785.0	1.06%	1109.7	0.87%	motif file (matrix)	svg
109		dof45(C2C2dof)/col-dof45-DAP-Seq(GSE60143)/Homer	1e-15	-3.554e+01	0.0000	54001.0	31.94%	39711.0	31.03%	motif file (matrix)	svg
110		HAT5(Homeobox)/colamp-HAT5-DAP-Seq(GSE60143)/Homer	1e-15	-3.532e+01	0.0000	2765.0	1.64%	1791.9	1.40%	motif file (matrix)	svg
111		OBP1(C2C2dof)/col-OBP1-DAP-Seq(GSE60143)/Homer	1e-15	-3.491e+01	0.0000	59857.0	35.40%	44124.4	34.48%	motif file (matrix)	svg
112		WRKY55(WRKY)/col-WRKY55-DAP-Seq(GSE60143)/Homer	1e-15	-3.486e+01	0.0000	14210.0	8.40%	10080.8	7.88%	motif file (matrix)	svg
113		At4g18890(BZR)/col-At4g18890-DAP-Seq(GSE60143)/Homer	1e-15	-3.486e+01	0.0000	1857.0	1.10%	1162.7	0.91%	motif file (matrix)	svg
114		RAV1(RAV)/colamp-RAV1-DAP-Seq(GSE60143)/Homer	1e-15	-3.460e+01	0.0000	12705.0	7.51%	8978.5	7.02%	motif file (matrix)	svg
115		MYB10(MYB)/col-MYB10-DAP-Seq(GSE60143)/Homer	1e-15	-3.457e+01	0.0000	5525.0	3.27%	3757.3	2.94%	motif file (matrix)	svg
116		MYB118(MYB)/colamp-MYB118-DAP-Seq(GSE60143)/Homer	1e-14	-3.430e+01	0.0000	6849.0	4.05%	4713.0	3.68%	motif file (matrix)	svg
117		GLIS3(Zf)/Thyroid-Glis3.GFP-ChIP-Seq(GSE103297)/Homer	1e-14	-3.426e+01	0.0000	14781.0	8.74%	10506.5	8.21%	motif file (matrix)	svg
118		At3g09600(MYBrelated)/colamp-At3g09600-DAP-Seq(GSE60143)/Homer	1e-14	-3.381e+01	0.0000	10061.0	5.95%	7051.9	5.51%	motif file (matrix)	svg
119		AT3G51470(DBP)/col-AT3G51470-DAP-Seq(GSE60143)/Homer	1e-14	-3.375e+01	0.0000	27782.0	16.43%	20135.1	15.73%	motif file (matrix)	svg
120		COUP-TFII(NR)/K562-NR2F1-ChIP-Seq(Encode)/Homer	1e-14	-3.343e+01	0.0000	17980.0	10.63%	12873.8	10.06%	motif file (matrix)	svg
121		AT5G66940(C2C2dof)/col-AT5G66940-DAP-Seq(GSE60143)/Homer	1e-14	-3.342e+01	0.0000	43698.0	25.85%	32021.0	25.02%	motif file (matrix)	svg
122		ATHB21(HB)/colamp-ATHB21-DAP-Seq(GSE60143)/Homer	1e-13	-3.222e+01	0.0000	11440.0	6.77%	8074.4	6.31%	motif file (matrix)	svg
123		ATHB6(Homeobox)/col-ATHB6-DAP-Seq(GSE60143)/Homer	1e-13	-3.205e+01	0.0000	26328.0	15.57%	19078.5	14.91%	motif file (matrix)	svg
124		HNF4a(NR),DR1/HepG2-HNF4a-ChIP-Seq(GSE25021)/Homer	1e-13	-3.196e+01	0.0000	4843.0	2.86%	3284.6	2.57%	motif file (matrix)	svg
125		Meis1(Homeobox)/MastCells-Meis1-ChIP-Seq(GSE48085)/Homer	1e-13	-3.188e+01	0.0000	25387.0	15.02%	18381.1	14.36%	motif file (matrix)	svg
126		MYB30(MYB)/colamp-MYB30-DAP-Seq(GSE60143)/Homer	1e-13	-3.153e+01	0.0000	15531.0	9.19%	11088.8	8.66%	motif file (matrix)	svg
127		PCF/Arabidopsis-Promoters/Homer	1e-13	-3.101e+01	0.0000	4889.0	2.89%	3323.3	2.60%	motif file (matrix)	svg
128		AT5G45580(G2like)/colamp-AT5G45580-DAP-Seq(GSE60143)/Homer	1e-13	-3.024e+01	0.0000	27270.0	16.13%	19805.1	15.48%	motif file (matrix)	svg
129		Bcl6(Zf)/Liver-Bcl6-ChIP-Seq(GSE31578)/Homer	1e-13	-3.010e+01	0.0000	14213.0	8.41%	10133.1	7.92%	motif file (matrix)	svg
130		Slug(Zf)/Mesoderm-Snai2-ChIP-Seq(GSE61475)/Homer	1e-12	-2.975e+01	0.0000	6413.0	3.79%	4431.0	3.46%	motif file (matrix)	svg
131		NPAS2(bHLH)/Liver-NPAS2-ChIP-Seq(GSE39860)/Homer	1e-12	-2.970e+01	0.0000	13733.0	8.12%	9784.5	7.65%	motif file (matrix)	svg
132		Atf4(bZIP)/MEF-Atf4-ChIP-Seq(GSE35681)/Homer	1e-12	-2.932e+01	0.0000	5142.0	3.04%	3517.0	2.75%	motif file (matrix)	svg
133		REM19(REM)/colamp-REM19-DAP-Seq(GSE60143)/Homer	1e-12	-2.923e+01	0.0000	13927.0	8.24%	9932.5	7.76%	motif file (matrix)	svg
134		WRKY29(WRKY)/colamp-WRKY29-DAP-Seq(GSE60143)/Homer	1e-12	-2.919e+01	0.0000	13042.0	7.71%	9281.1	7.25%	motif file (matrix)	svg
135		Chop(bZIP)/MEF-Chop-ChIP-Seq(GSE35681)/Homer	1e-12	-2.904e+01	0.0000	3553.0	2.10%	2379.8	1.86%	motif file (matrix)	svg
136		WRKY50(WRKY)/col-WRKY50-DAP-Seq(GSE60143)/Homer	1e-12	-2.892e+01	0.0000	12747.0	7.54%	9067.1	7.08%	motif file (matrix)	svg
137		HIF-1b(HLH)/T47D-HIF1b-ChIP-Seq(GSE59937)/Homer	1e-12	-2.862e+01	0.0000	15721.0	9.30%	11262.8	8.80%	motif file (matrix)	svg
138		ATHB53(HB)/col-ATHB53-DAP-Seq(GSE60143)/Homer	1e-12	-2.859e+01	0.0000	11121.0	6.58%	7876.9	6.15%	motif file (matrix)	svg
139		HOXA1(Homeobox)/mES-Hoxa1-ChIP-Seq(SRP084292)/Homer	1e-12	-2.853e+01	0.0000	4113.0	2.43%	2782.8	2.17%	motif file (matrix)	svg
140		HAT2(Homeobox)/colamp-HAT2-DAP-Seq(GSE60143)/Homer	1e-12	-2.830e+01	0.0000	17080.0	10.10%	12270.8	9.59%	motif file (matrix)	svg
141		Tbet(T-box)/CD8-Tbet-ChIP-Seq(GSE33802)/Homer	1e-12	-2.829e+01	0.0000	16264.0	9.62%	11667.8	9.12%	motif file (matrix)	svg
142		AT2G38300(G2like)/col-AT2G38300-DAP-Seq(GSE60143)/Homer	1e-12	-2.810e+01	0.0000	24730.0	14.63%	17947.6	14.02%	motif file (matrix)	svg
143		IRF:BATF(IRF:bZIP)/pDC-Irf8-ChIP-Seq(GSE66899)/Homer	1e-12	-2.807e+01	0.0000	1653.0	0.98%	1046.7	0.82%	motif file (matrix)	svg
144		At5g52660(MYBrelated)/colamp-At5g52660-DAP-Seq(GSE60143)/Homer	1e-12	-2.797e+01	0.0000	9781.0	5.79%	6900.5	5.39%	motif file (matrix)	svg
145		MYB57(MYB)/col-MYB57-DAP-Seq(GSE60143)/Homer	1e-12	-2.791e+01	0.0000	7570.0	4.48%	5286.6	4.13%	motif file (matrix)	svg
146		RBPJ:Ebox(?,bHLH)/Panc1-Rbpj1-ChIP-Seq(GSE47459)/Homer	1e-12	-2.782e+01	0.0000	3619.0	2.14%	2434.0	1.90%	motif file (matrix)	svg
147		AtGRF6(GRF)/col-AtGRF6-DAP-Seq(GSE60143)/Homer	1e-11	-2.750e+01	0.0000	20030.0	11.85%	14465.7	11.30%	motif file (matrix)	svg
148		SeqBias: polyA-repeat	1e-11	-2.745e+01	0.0000	168482.0	99.65%	127389.1	99.54%	motif file (matrix)	svg
149		Znf263(Zf)/K562-Znf263-ChIP-Seq(GSE31477)/Homer	1e-11	-2.733e+01	0.0000	24073.0	14.24%	17470.0	13.65%	motif file (matrix)	svg
150		ZNF415(Zf)/HEK293-ZNF415.GFP-ChIP-Seq(GSE58341)/Homer	1e-11	-2.709e+01	0.0000	5392.0	3.19%	3712.7	2.90%	motif file (matrix)	svg
151		At4g38000(C2C2dof)/col-At4g38000-DAP-Seq(GSE60143)/Homer	1e-11	-2.699e+01	0.0000	26419.0	15.63%	19221.6	15.02%	motif file (matrix)	svg
152		MYB116(MYB)/colamp-MYB116-DAP-Seq(GSE60143)/Homer	1e-11	-2.698e+01	0.0000	12710.0	7.52%	9061.1	7.08%	motif file (matrix)	svg
153		WIP5(C2H2)/colamp-WIP5-DAP-Seq(GSE60143)/Homer	1e-11	-2.697e+01	0.0000	15216.0	9.00%	10909.6	8.52%	motif file (matrix)	svg
154		Fox:Ebox(Forkhead,bHLH)/Panc1-Foxa2-ChIP-Seq(GSE47459)/Homer	1e-11	-2.671e+01	0.0000	19573.0	11.58%	14137.6	11.05%	motif file (matrix)	svg
155		Hnf6b(Homeobox)/LNCaP-Hnf6b-ChIP-Seq(GSE106305)/Homer	1e-11	-2.658e+01	0.0000	20184.0	11.94%	14592.1	11.40%	motif file (matrix)	svg
156		Stat3+il21(Stat)/CD4-Stat3-ChIP-Seq(GSE19198)/Homer	1e-11	-2.604e+01	0.0000	7685.0	4.55%	5386.8	4.21%	motif file (matrix)	svg
157		TRP2(MYBrelated)/colamp-TRP2-DAP-Seq(GSE60143)/Homer	1e-11	-2.591e+01	0.0000	3277.0	1.94%	2200.6	1.72%	motif file (matrix)	svg
158		MYB39(MYB)/col-MYB39-DAP-Seq(GSE60143)/Homer	1e-11	-2.577e+01	0.0000	2367.0	1.40%	1555.3	1.22%	motif file (matrix)	svg
159		WRKY27(WRKY)/colamp-WRKY27-DAP-Seq(GSE60143)/Homer	1e-11	-2.566e+01	0.0000	11293.0	6.68%	8033.6	6.28%	motif file (matrix)	svg
160		MYB13(MYB)/col-MYB13-DAP-Seq(GSE60143)/Homer	1e-10	-2.508e+01	0.0000	8896.0	5.26%	6280.8	4.91%	motif file (matrix)	svg
161		Barx1(Homeobox)/Stomach-Barx1.3xFlag-ChIP-Seq(GSE69483)/Homer	1e-10	-2.500e+01	0.0000	10136.0	5.99%	7190.2	5.62%	motif file (matrix)	svg
162		TCP17(TCP)/col-TCP17-DAP-Seq(GSE60143)/Homer	1e-10	-2.496e+01	0.0000	631.0	0.37%	362.1	0.28%	motif file (matrix)	svg
163		WRKY25(WRKY)/colamp-WRKY25-DAP-Seq(GSE60143)/Homer	1e-10	-2.495e+01	0.0000	15443.0	9.13%	11102.4	8.68%	motif file (matrix)	svg
164		At2g01060(G2like)/colamp-At2g01060-DAP-Seq(GSE60143)/Homer	1e-10	-2.482e+01	0.0000	61515.0	36.38%	45573.4	35.61%	motif file (matrix)	svg
165		bZIP18(bZIP)/colamp-bZIP18-DAP-Seq(GSE60143)/Homer	1e-10	-2.462e+01	0.0000	85100.0	50.33%	63388.6	49.53%	motif file (matrix)	svg
166		ZEB2(Zf)/SNU398-ZEB2-ChIP-Seq(GSE103048)/Homer	1e-10	-2.455e+01	0.0000	12603.0	7.45%	9010.7	7.04%	motif file (matrix)	svg
167		Arnt:Ahr(bHLH)/MCF7-Arnt-ChIP-Seq(Lo_et_al.)/Homer	1e-10	-2.447e+01	0.0000	8810.0	5.21%	6223.7	4.86%	motif file (matrix)	svg
168		AT2G28920(ND)/col-AT2G28920-DAP-Seq(GSE60143)/Homer	1e-10	-2.444e+01	0.0000	4982.0	2.95%	3435.6	2.68%	motif file (matrix)	svg
169		WRKY15(WRKY)/col-WRKY15-DAP-Seq(GSE60143)/Homer	1e-10	-2.438e+01	0.0000	12325.0	7.29%	8807.2	6.88%	motif file (matrix)	svg
170		IDD7(C2H2)/col-IDD7-DAP-Seq(GSE60143)/Homer	1e-10	-2.426e+01	0.0000	5349.0	3.16%	3702.9	2.89%	motif file (matrix)	svg
171		ERRg(NR)/Kidney-ESRRG-ChIP-Seq(GSE104905)/Homer	1e-10	-2.414e+01	0.0000	9542.0	5.64%	6763.7	5.28%	motif file (matrix)	svg
172		IRF3(IRF)/BMDM-Irf3-ChIP-Seq(GSE67343)/Homer	1e-10	-2.413e+01	0.0000	3345.0	1.98%	2259.5	1.77%	motif file (matrix)	svg
173		PHA-4(Forkhead)/cElegans-Embryos-PHA4-ChIP-Seq(modEncode)/Homer	1e-10	-2.412e+01	0.0000	68915.0	40.76%	51168.8	39.98%	motif file (matrix)	svg
174		MYB40(MYB)/col-MYB40-DAP-Seq(GSE60143)/Homer	1e-10	-2.393e+01	0.0000	4815.0	2.85%	3318.1	2.59%	motif file (matrix)	svg
175		WRKY6(WRKY)/colamp-WRKY6-DAP-Seq(GSE60143)/Homer	1e-10	-2.390e+01	0.0000	10591.0	6.26%	7536.7	5.89%	motif file (matrix)	svg
176		TGA9(bZIP)/colamp-TGA9-DAP-Seq(GSE60143)/Homer	1e-10	-2.373e+01	0.0000	21415.0	12.67%	15548.4	12.15%	motif file (matrix)	svg
177		BPC1(BBRBPC)/colamp-BPC1-DAP-Seq(GSE60143)/Homer	1e-10	-2.370e+01	0.0000	8010.0	4.74%	5645.6	4.41%	motif file (matrix)	svg
178		BZR1(BZR)/col-BZR1-DAP-Seq(GSE60143)/Homer	1e-10	-2.355e+01	0.0000	901.0	0.53%	546.8	0.43%	motif file (matrix)	svg
179		Pdx1(Homeobox)/Islet-Pdx1-ChIP-Seq(SRA008281)/Homer	1e-10	-2.352e+01	0.0000	20049.0	11.86%	14536.5	11.36%	motif file (matrix)	svg
180		ANAC079(NAC)/colamp-ANAC079-DAP-Seq(GSE60143)/Homer	1e-10	-2.347e+01	0.0000	6069.0	3.59%	4231.4	3.31%	motif file (matrix)	svg
181		MGP(C2H2)/colamp-MGP-DAP-Seq(GSE60143)/Homer	1e-10	-2.343e+01	0.0000	3346.0	1.98%	2264.2	1.77%	motif file (matrix)	svg
182		SeqBias: G/A bias	1e-10	-2.310e+01	0.0000	168064.0	99.40%	127051.1	99.27%	motif file (matrix)	svg
183		AT1G47655(C2C2dof)/colamp-AT1G47655-DAP-Seq(GSE60143)/Homer	1e-10	-2.309e+01	0.0000	71484.0	42.28%	53131.5	41.52%	motif file (matrix)	svg
184		ATHB18(Homeobox)/colamp-ATHB18-DAP-Seq(GSE60143)/Homer	1e-9	-2.300e+01	0.0000	3320.0	1.96%	2248.3	1.76%	motif file (matrix)	svg
185		MYB119(MYB)/colamp-MYB119-DAP-Seq(GSE60143)/Homer	1e-9	-2.249e+01	0.0000	6645.0	3.93%	4659.4	3.64%	motif file (matrix)	svg
186		Pitx1:Ebox(Homeobox,bHLH)/Hindlimb-Pitx1-ChIP-Seq(GSE41591)/Homer	1e-9	-2.248e+01	0.0000	3208.0	1.90%	2171.4	1.70%	motif file (matrix)	svg
187		NAC2(NAC)/colamp-NAC2-DAP-Seq(GSE60143)/Homer	1e-9	-2.175e+01	0.0000	6112.0	3.61%	4277.9	3.34%	motif file (matrix)	svg
188		HAP3(CCAATHAP3)/col-HAP3-DAP-Seq(GSE60143)/Homer	1e-9	-2.149e+01	0.0000	4791.0	2.83%	3319.7	2.59%	motif file (matrix)	svg
189		NF1-halfsite(CTF)/LNCaP-NF1-ChIP-Seq(Unpublished)/Homer	1e-9	-2.148e+01	0.0000	25188.0	14.90%	18394.5	14.37%	motif file (matrix)	svg
190		MYB41(MYB)/col-MYB41-DAP-Seq(GSE60143)/Homer	1e-9	-2.144e+01	0.0000	5029.0	2.97%	3492.7	2.73%	motif file (matrix)	svg
191		GBF3(bZIP)/Arabidopsis-GBF3-ChIP-Seq(GSE80564)/Homer	1e-9	-2.143e+01	0.0000	4680.0	2.77%	3239.8	2.53%	motif file (matrix)	svg
192		TEAD2(TEA)/Py2T-Tead2-ChIP-Seq(GSE55709)/Homer	1e-9	-2.124e+01	0.0000	6354.0	3.76%	4458.3	3.48%	motif file (matrix)	svg
193		GRE(NR),IR3/RAW264.7-GRE-ChIP-Seq(Unpublished)/Homer	1e-9	-2.124e+01	0.0000	3523.0	2.08%	2405.6	1.88%	motif file (matrix)	svg
194		PHV(HB)/col-PHV-DAP-Seq(GSE60143)/Homer	1e-9	-2.124e+01	0.0000	6706.0	3.97%	4715.5	3.68%	motif file (matrix)	svg
195		AT3G52440(C2C2dof)/colamp-AT3G52440-DAP-Seq(GSE60143)/Homer	1e-9	-2.121e+01	0.0000	50397.0	29.81%	37286.0	29.13%	motif file (matrix)	svg
196		AT2G33550(Trihelix)/colamp-AT2G33550-DAP-Seq(GSE60143)/Homer	1e-9	-2.114e+01	0.0000	47693.0	28.21%	35256.0	27.55%	motif file (matrix)	svg
197		Hoxa13(Homeobox)/ChickenMSG-Hoxa13.Flag-ChIP-Seq(GSE86088)/Homer	1e-9	-2.080e+01	0.0000	52036.0	30.78%	38528.2	30.10%	motif file (matrix)	svg
198		Unknown4/Arabidopsis-Promoters/Homer	1e-8	-2.027e+01	0.0000	11420.0	6.75%	8189.4	6.40%	motif file (matrix)	svg
199		Erra(NR)/HepG2-Erra-ChIP-Seq(GSE31477)/Homer	1e-8	-2.027e+01	0.0000	26810.0	15.86%	19627.0	15.34%	motif file (matrix)	svg
200		NPAS(bHLH)/Liver-NPAS-ChIP-Seq(GSE39860)/Homer	1e-8	-2.019e+01	0.0000	24998.0	14.79%	18275.8	14.28%	motif file (matrix)	svg
201		LRF(Zf)/Erythroblasts-ZBTB7A-ChIP-Seq(GSE74977)/Homer	1e-8	-2.016e+01	0.0000	17633.0	10.43%	12792.4	10.00%	motif file (matrix)	svg
202		LIN-39(Homeobox)/cElegans.L3-LIN39-ChIP-Seq(modEncode)/Homer	1e-8	-2.008e+01	0.0000	23900.0	14.14%	17459.0	13.64%	motif file (matrix)	svg
203		NF1:FOXA1(CTF,Forkhead)/LNCAP-FOXA1-ChIP-Seq(GSE27824)/Homer	1e-8	-2.006e+01	0.0000	1015.0	0.60%	635.9	0.50%	motif file (matrix)	svg
204		MYB96(MYB)/colamp-MYB96-DAP-Seq(GSE60143)/Homer	1e-8	-1.997e+01	0.0000	13055.0	7.72%	9401.9	7.35%	motif file (matrix)	svg
205		EGL-5(Homeobox)/cElegans-L3-EGL5-ChIP-Seq(modEncode)/Homer	1e-8	-1.987e+01	0.0000	33981.0	20.10%	24996.6	19.53%	motif file (matrix)	svg
206		GATA3(Zf),DR8/iTreg-Gata3-ChIP-Seq(GSE20898)/Homer	1e-8	-1.911e+01	0.0000	1254.0	0.74%	804.1	0.63%	motif file (matrix)	svg
207		ANAC004(NAC)/colamp-ANAC004-DAP-Seq(GSE60143)/Homer	1e-8	-1.883e+01	0.0000	3817.0	2.26%	2634.1	2.06%	motif file (matrix)	svg
208		Tcf7(HMG)/GM12878-TCF7-ChIP-Seq(Encode)/Homer	1e-8	-1.875e+01	0.0000	3841.0	2.27%	2652.4	2.07%	motif file (matrix)	svg
209		c-Myc(bHLH)/mES-cMyc-ChIP-Seq(GSE11431)/Homer	1e-8	-1.866e+01	0.0000	5864.0	3.47%	4124.8	3.22%	motif file (matrix)	svg
210		ZEB1(Zf)/PDAC-ZEB1-ChIP-Seq(GSE64557)/Homer	1e-8	-1.863e+01	0.0000	26465.0	15.65%	19399.5	15.16%	motif file (matrix)	svg
211		STAT1(Stat)/HelaS3-STAT1-ChIP-Seq(GSE12782)/Homer	1e-7	-1.837e+01	0.0000	3014.0	1.78%	2058.4	1.61%	motif file (matrix)	svg
212		GATA(Zf),IR4/iTreg-Gata3-ChIP-Seq(GSE20898)/Homer	1e-7	-1.828e+01	0.0000	1150.0	0.68%	735.6	0.57%	motif file (matrix)	svg
213		PAX3:FKHR-fusion(Paired,Homeobox)/Rh4-PAX3:FKHR-ChIP-Seq(GSE19063)/Homer	1e-7	-1.827e+01	0.0000	3684.0	2.18%	2542.8	1.99%	motif file (matrix)	svg
214		ATHB7(Homeobox)/col-ATHB7-DAP-Seq(GSE60143)/Homer	1e-7	-1.808e+01	0.0000	15015.0	8.88%	10880.7	8.50%	motif file (matrix)	svg
215		TCP1(TCP)/col-TCP1-DAP-Seq(GSE60143)/Homer	1e-7	-1.787e+01	0.0000	7347.0	4.35%	5218.5	4.08%	motif file (matrix)	svg
216		ZNF264(Zf)/HEK293-ZNF264.GFP-ChIP-Seq(GSE58341)/Homer	1e-7	-1.777e+01	0.0000	10264.0	6.07%	7367.4	5.76%	motif file (matrix)	svg
217		Pit1(Homeobox)/GCrat-Pit1-ChIP-Seq(GSE58009)/Homer	1e-7	-1.771e+01	0.0000	21506.0	12.72%	15715.9	12.28%	motif file (matrix)	svg
218		HSFA6B(HSF)/colamp-HSFA6B-DAP-Seq(GSE60143)/Homer	1e-7	-1.766e+01	0.0000	5780.0	3.42%	4073.0	3.18%	motif file (matrix)	svg
219		STOP1(C2H2)/colamp-STOP1-DAP-Seq(GSE60143)/Homer	1e-7	-1.745e+01	0.0000	5405.0	3.20%	3800.2	2.97%	motif file (matrix)	svg
220		WRKY40(WRKY)/colamp-WRKY40-DAP-Seq(GSE60143)/Homer	1e-7	-1.741e+01	0.0000	6547.0	3.87%	4637.0	3.62%	motif file (matrix)	svg
221		ANAC058(NAC)/col-ANAC058-DAP-Seq(GSE60143)/Homer	1e-7	-1.736e+01	0.0000	5449.0	3.22%	3833.8	3.00%	motif file (matrix)	svg
222		ZNF669(Zf)/HEK293-ZNF669.GFP-ChIP-Seq(GSE58341)/Homer	1e-7	-1.733e+01	0.0000	655.0	0.39%	398.2	0.31%	motif file (matrix)	svg
223		bcd(Homeobox)/Embryo-Bcd-ChIP-Seq(GSE86966)/Homer	1e-7	-1.726e+01	0.0000	19148.0	11.33%	13966.5	10.91%	motif file (matrix)	svg
224		AT3G10113(MYBrelated)/col-AT3G10113-DAP-Seq(GSE60143)/Homer	1e-7	-1.705e+01	0.0000	10782.0	6.38%	7759.1	6.06%	motif file (matrix)	svg
225		HAT1(Homeobox)/col-HAT1-DAP-Seq(GSE60143)/Homer	1e-7	-1.704e+01	0.0000	3111.0	1.84%	2137.2	1.67%	motif file (matrix)	svg
226		THRb(NR)/HepG2-THRb.Flag-ChIP-Seq(Encode)/Homer	1e-7	-1.696e+01	0.0000	5932.0	3.51%	4190.8	3.27%	motif file (matrix)	svg
227		TEAD(TEA)/Fibroblast-PU.1-ChIP-Seq(Unpublished)/Homer	1e-7	-1.690e+01	0.0000	9226.0	5.46%	6612.9	5.17%	motif file (matrix)	svg
228		THRa(NR)/C17.2-THRa-ChIP-Seq(GSE38347)/Homer	1e-7	-1.690e+01	0.0000	4655.0	2.75%	3258.3	2.55%	motif file (matrix)	svg
229		ELF5(ETS)/T47D-ELF5-ChIP-Seq(GSE30407)/Homer	1e-7	-1.688e+01	0.0000	10718.0	6.34%	7714.2	6.03%	motif file (matrix)	svg
230		WRKY71(WRKY)/col-WRKY71-DAP-Seq(GSE60143)/Homer	1e-7	-1.641e+01	0.0000	9253.0	5.47%	6638.9	5.19%	motif file (matrix)	svg
231		ARE(NR)/LNCAP-AR-ChIP-Seq(GSE27824)/Homer	1e-7	-1.633e+01	0.0000	2928.0	1.73%	2010.6	1.57%	motif file (matrix)	svg
232		Tbox:Smad(T-box,MAD)/ESCd5-Smad2_3-ChIP-Seq(GSE29422)/Homer	1e-7	-1.631e+01	0.0000	2426.0	1.43%	1649.2	1.29%	motif file (matrix)	svg
233		NFIL3(bZIP)/HepG2-NFIL3-ChIP-Seq(Encode)/Homer	1e-7	-1.623e+01	0.0000	16401.0	9.70%	11940.0	9.33%	motif file (matrix)	svg
234		CEBP:AP1(bZIP)/ThioMac-CEBPb-ChIP-Seq(GSE21512)/Homer	1e-7	-1.619e+01	0.0000	14328.0	8.47%	10399.7	8.13%	motif file (matrix)	svg
235		AT3G10030(Trihelix)/colamp-AT3G10030-DAP-Seq(GSE60143)/Homer	1e-6	-1.602e+01	0.0000	10509.0	6.22%	7571.2	5.92%	motif file (matrix)	svg
236		IRF8(IRF)/BMDM-IRF8-ChIP-Seq(GSE77884)/Homer	1e-6	-1.572e+01	0.0000	3352.0	1.98%	2321.8	1.81%	motif file (matrix)	svg
237		HSF3(HSF)/colamp-HSF3-DAP-Seq(GSE60143)/Homer	1e-6	-1.572e+01	0.0000	11052.0	6.54%	7977.4	6.23%	motif file (matrix)	svg
238		SeqBias: A/T bias	1e-6	-1.556e+01	0.0000	158293.0	93.62%	119425.9	93.32%	motif file (matrix)	svg
239		Mef2d(MADS)/Retina-Mef2d-ChIP-Seq(GSE61391)/Homer	1e-6	-1.525e+01	0.0000	2629.0	1.55%	1802.3	1.41%	motif file (matrix)	svg
240		MYB3R1(MYB)/col-MYB3R1-DAP-Seq(GSE60143)/Homer	1e-6	-1.522e+01	0.0000	4841.0	2.86%	3409.1	2.66%	motif file (matrix)	svg
241		CRC(C2C2YABBY)/col-CRC-DAP-Seq(GSE60143)/Homer	1e-6	-1.521e+01	0.0000	24977.0	14.77%	18355.6	14.34%	motif file (matrix)	svg
242		At5g66730(C2H2)/colamp-At5g66730-DAP-Seq(GSE60143)/Homer	1e-6	-1.495e+01	0.0000	3688.0	2.18%	2571.5	2.01%	motif file (matrix)	svg
243		Unknown3/Drosophila-Promoters/Homer	1e-6	-1.489e+01	0.0000	1684.0	1.00%	1127.9	0.88%	motif file (matrix)	svg
244		ANAC062(NAC)/colamp-ANAC062-DAP-Seq(GSE60143)/Homer	1e-6	-1.485e+01	0.0000	4073.0	2.41%	2852.7	2.23%	motif file (matrix)	svg

245	AIL7(AP2EREBP)/colamp-AIL7-DAP-Seq(GSE60143)/Homer	1e-6	-1.483e+01	0.0000	4803.0	2.84%	3386.0	2.65%	motif file (matrix)	svg
246	At1g78700(BZR)/col-At1g78700-DAP-Seq(GSE60143)/Homer	1e-6	-1.481e+01	0.0000	3069.0	1.82%	2123.8	1.66%	motif file (matrix)	svg
247	MafA(bZIP)/Islet-MafA-ChIP-Seq(GSE30298)/Homer	1e-6	-1.480e+01	0.0000	10790.0	6.38%	7796.6	6.09%	motif file (matrix)	svg
248	Stat3(Stat)/mES-Stat3-ChIP-Seq(GSE11431)/Homer	1e-6	-1.477e+01	0.0000	4712.0	2.79%	3319.8	2.59%	motif file (matrix)	svg
249	Cux2(Homeobox)/Liver-Cux2-ChIP-Seq(GSE35985)/Homer	1e-6	-1.473e+01	0.0000	9306.0	5.50%	6699.9	5.24%	motif file (matrix)	svg
250	NTM2(NAC)/col-NTM2-DAP-Seq(GSE60143)/Homer	1e-6	-1.466e+01	0.0000	5826.0	3.45%	4136.7	3.23%	motif file (matrix)	svg
251	At4g36780(BZR)/col-At4g36780-DAP-Seq(GSE60143)/Homer	1e-6	-1.456e+01	0.0000	3174.0	1.88%	2201.4	1.72%	motif file (matrix)	svg
252	PAX6(Paired,Homeobox)/Forebrain-Pax6-ChIP-Seq(GSE66961)/Homer	1e-6	-1.438e+01	0.0000	1028.0	0.61%	666.2	0.52%	motif file (matrix)	svg
253	AT2G40260(G2like)/colamp-AT2G40260-DAP-Seq(GSE60143)/Homer	1e-6	-1.435e+01	0.0000	30215.0	17.87%	22295.7	17.42%	motif file (matrix)	svg
254	O2(bZIP)/Corn-O2-ChIP-Seq(GSE63991)/Homer	1e-6	-1.421e+01	0.0000	1304.0	0.77%	861.1	0.67%	motif file (matrix)	svg
255	HNF6(Homeobox)/Liver-Hnf6-ChIP-Seq(ERP000394)/Homer	1e-6	-1.410e+01	0.0000	11909.0	7.04%	8636.9	6.75%	motif file (matrix)	svg
256	WRKY22(WRKY)/colamp-WRKY22-DAP-Seq(GSE60143)/Homer	1e-5	-1.359e+01	0.0000	7040.0	4.16%	5042.0	3.94%	motif file (matrix)	svg
257	ATHB5(HB)/colamp-ATHB5-DAP-Seq(GSE60143)/Homer	1e-5	-1.336e+01	0.0000	19871.0	11.75%	14578.9	11.39%	motif file (matrix)	svg
258	TATA-box/SacCer-Promoters/Homer	1e-5	-1.327e+01	0.0000	21740.0	12.86%	15976.4	12.48%	motif file (matrix)	svg
259	At2g03500(G2like)/col-At2g03500-DAP-Seq(GSE60143)/Homer	1e-5	-1.324e+01	0.0000	4873.0	2.88%	3452.9	2.70%	motif file (matrix)	svg
260	Otx2(Homeobox)/EpiLC-Otx2-ChIP-Seq(GSE56098)/Homer	1e-5	-1.319e+01	0.0000	13437.0	7.95%	9786.8	7.65%	motif file (matrix)	svg
261	DAG2(C2C2dof)/col-DAG2-DAP-Seq(GSE60143)/Homer	1e-5	-1.310e+01	0.0000	35156.0	20.79%	26032.1	20.34%	motif file (matrix)	svg
262	STAT6(Stat)/Macrophage-Stat6-ChIP-Seq(GSE38377)/Homer	1e-5	-1.302e+01	0.0000	6235.0	3.69%	4455.6	3.48%	motif file (matrix)	svg
263	AR-halfsite(NR)/LNCaP-AR-ChIP-Seq(GSE27824)/Homer	1e-5	-1.301e+01	0.0000	59017.0	34.91%	43993.9	34.38%	motif file (matrix)	svg
264	NF1(CTF)/LNCAP-NF1-ChIP-Seq(Unpublished)/Homer	1e-5	-1.300e+01	0.0000	2855.0	1.69%	1982.7	1.55%	motif file (matrix)	svg
265	NTM1(NAC)/col-NTM1-DAP-Seq(GSE60143)/Homer	1e-5	-1.289e+01	0.0000	5492.0	3.25%	3911.0	3.06%	motif file (matrix)	svg
266	WRKY24(WRKY)/colamp-WRKY24-DAP-Seq(GSE60143)/Homer	1e-5	-1.271e+01	0.0000	10869.0	6.43%	7886.8	6.16%	motif file (matrix)	svg
267	FUS3(ABI3VP1)/col-FUS3-DAP-Seq(GSE60143)/Homer	1e-5	-1.270e+01	0.0000	15733.0	9.31%	11504.1	8.99%	motif file (matrix)	svg
268	STAT5(Stat)/mCD4+-Stat5-ChIP-Seq(GSE12346)/Homer	1e-5	-1.263e+01	0.0000	2804.0	1.66%	1948.9	1.52%	motif file (matrix)	svg
269	At5g29000(G2like)/col-At5g29000-DAP-Seq(GSE60143)/Homer	1e-5	-1.252e+01	0.0000	4815.0	2.85%	3417.7	2.67%	motif file (matrix)	svg
270	ANAC016(NAC)/col-ANAC016-DAP-Seq(GSE60143)/Homer	1e-5	-1.248e+01	0.0000	7185.0	4.25%	5162.8	4.03%	motif file (matrix)	svg
271	ZNF189(Zf)/HEK293-ZNF189.GFP-ChIP-Seq(GSE58341)/Homer	1e-5	-1.247e+01	0.0000	9846.0	5.82%	7131.2	5.57%	motif file (matrix)	svg
272	MYB73(MYB)/col-MYB73-DAP-Seq(GSE60143)/Homer	1e-5	-1.246e+01	0.0000	43753.0	25.88%	32511.4	25.40%	motif file (matrix)	svg
273	bZIP:IRF(bZIP,IRF)/Th17-BatF-ChIP-Seq(GSE39756)/Homer	1e-5	-1.237e+01	0.0000	6253.0	3.70%	4476.4	3.50%	motif file (matrix)	svg
274	MYB44(MYB)/colamp-MYB44-DAP-Seq(GSE60143)/Homer	1e-5	-1.233e+01	0.0000	1400.0	0.83%	939.4	0.73%	motif file (matrix)	svg
275	WRKY42(WRKY)/colamp-WRKY42-DAP-Seq(GSE60143)/Homer	1e-5	-1.216e+01	0.0000	3339.0	1.97%	2341.4	1.83%	motif file (matrix)	svg
276	Tlx?(NR)/NPC-H3K4me1-ChIP-Seq(GSE16256)/Homer	1e-5	-1.202e+01	0.0000	3318.0	1.96%	2327.5	1.82%	motif file (matrix)	svg
277	Unknown4/Drosophila-Promoters/Homer	1e-5	-1.201e+01	0.0000	2720.0	1.61%	1892.4	1.48%	motif file (matrix)	svg
278	ERG(ETS)/VCaP-ERG-ChIP-Seq(GSE14097)/Homer	1e-5	-1.192e+01	0.0000	20885.0	12.35%	15366.4	12.01%	motif file (matrix)	svg
279	IDD2(C2H2)/colamp-IDD2-DAP-Seq(GSE60143)/Homer	1e-5	-1.191e+01	0.0000	1771.0	1.05%	1207.6	0.94%	motif file (matrix)	svg
280	ZNF711(Zf)/SHSY5Y-ZNF711-ChIP-Seq(GSE20673)/Homer	1e-5	-1.173e+01	0.0000	16499.0	9.76%	12094.9	9.45%	motif file (matrix)	svg
281	ANAC020(NAC)/col-ANAC020-DAP-Seq(GSE60143)/Homer	1e-5	-1.159e+01	0.0000	5757.0	3.40%	4120.5	3.22%	motif file (matrix)	svg
282	WRKY33(WRKY)/col-WRKY33-DAP-Seq(GSE60143)/Homer	1e-4	-1.149e+01	0.0000	8817.0	5.21%	6383.9	4.99%	motif file (matrix)	svg
283	WRKY75(WRKY)/col-WRKY75-DAP-Seq(GSE60143)/Homer	1e-4	-1.148e+01	0.0000	11511.0	6.81%	8383.3	6.55%	motif file (matrix)	svg
284	ARF2(ARF)/col-ARF2-DAP-Seq(GSE60143)/Homer	1e-4	-1.146e+01	0.0000	45664.0	27.01%	33979.5	26.55%	motif file (matrix)	svg
285	PRDM1(Zf)/Hela-PRDM1-ChIP-Seq(GSE31477)/Homer	1e-4	-1.144e+01	0.0000	7322.0	4.33%	5277.3	4.12%	motif file (matrix)	svg
286	TCP3(TCP)/colamp-TCP3-DAP-Seq(GSE60143)/Homer	1e-4	-1.132e+01	0.0000	3234.0	1.91%	2272.6	1.78%	motif file (matrix)	svg
287	AT4G37180(G2like)/col-AT4G37180-DAP-Seq(GSE60143)/Homer	1e-4	-1.130e+01	0.0000	18013.0	10.65%	13233.1	10.34%	motif file (matrix)	svg
288	CEBP:CEBP(bZIP)/MEF-Chop-ChIP-Seq(GSE35681)/Homer	1e-4	-1.130e+01	0.0000	2311.0	1.37%	1601.7	1.25%	motif file (matrix)	svg
289	ZML2(C2C2gata)/col-ZML2-DAP-Seq(GSE60143)/Homer	1e-4	-1.119e+01	0.0000	2227.0	1.32%	1541.0	1.20%	motif file (matrix)	svg
290	Mef2a(MADS)/HL1-Mef2a.biotin-ChIP-Seq(GSE21529)/Homer	1e-4	-1.116e+01	0.0000	6452.0	3.82%	4638.1	3.62%	motif file (matrix)	svg
291	GRHL2(CP2)/HBE-GRHL2-ChIP-Seq(GSE46194)/Homer	1e-4	-1.112e+01	0.0001	5009.0	2.96%	3575.2	2.79%	motif file (matrix)	svg
292	ANAC075(NAC)/col-ANAC075-DAP-Seq(GSE60143)/Homer	1e-4	-1.105e+01	0.0001	3184.0	1.88%	2238.6	1.75%	motif file (matrix)	svg
293	FRS9(ND)/col-FRS9-DAP-Seq(GSE60143)/Homer	1e-4	-1.080e+01	0.0001	4028.0	2.38%	2858.3	2.23%	motif file (matrix)	svg
294	ZKSCAN1(Zf)/HepG2-ZKSCAN1-ChIP-Seq(Encode)/Homer	1e-4	-1.066e+01	0.0001	314.0	0.19%	187.7	0.15%	motif file (matrix)	svg
295	Pho4(bHLH)/Yeast-Pho4-ChIP-Seq(GSE29506)/Homer	1e-4	-1.061e+01	0.0001	1849.0	1.09%	1272.9	0.99%	motif file (matrix)	svg
296	WRKY31(WRKY)/colamp-WRKY31-DAP-Seq(GSE60143)/Homer	1e-4	-1.058e+01	0.0001	8337.0	4.93%	6041.7	4.72%	motif file (matrix)	svg
297	AGL95(ND)/col-AGL95-DAP-Seq(GSE60143)/Homer	1e-4	-1.051e+01	0.0001	777.0	0.46%	507.6	0.40%	motif file (matrix)	svg
298	CUC3(NAC)/col-CUC3-DAP-Seq(GSE60143)/Homer	1e-4	-1.046e+01	0.0001	3279.0	1.94%	2313.3	1.81%	motif file (matrix)	svg
299	STAT6(Stat)/CD4-Stat6-ChIP-Seq(GSE22104)/Homer	1e-4	-1.042e+01	0.0001	6283.0	3.72%	4523.6	3.53%	motif file (matrix)	svg
300	SeqBias: polyC-repeat	1e-4	-1.042e+01	0.0001	165696.0	98.00%	125243.5	97.86%	motif file (matrix)	svg
301	TATA-Box(TBP)/Promoter/Homer	1e-4	-1.039e+01	0.0001	29611.0	17.51%	21942.7	17.15%	motif file (matrix)	svg
302	AT1G76870(Trihelix)/col-AT1G76870-DAP-Seq(GSE60143)/Homer	1e-4	-1.036e+01	0.0001	7031.0	4.16%	5077.7	3.97%	motif file (matrix)	svg
303	MYB17(MYB)/colamp-MYB17-DAP-Seq(GSE60143)/Homer	1e-4	-1.035e+01	0.0001	7166.0	4.24%	5177.7	4.05%	motif file (matrix)	svg
304	AZF1(C2H2)/colamp-AZF1-DAP-Seq(GSE60143)/Homer	1e-4	-1.035e+01	0.0001	93301.0	55.18%	70004.1	54.70%	motif file (matrix)	svg
305	Zelda(Zf)/Embryo-zld-ChIP-Seq(GSE65441)/Homer	1e-4	-1.032e+01	0.0001	7765.0	4.59%	5621.3	4.39%	motif file (matrix)	svg
306	HNF1b(Homeobox)/PDAC-HNF1B-ChIP-Seq(GSE64557)/Homer	1e-4	-1.029e+01	0.0001	2329.0	1.38%	1622.1	1.27%	motif file (matrix)	svg
307	p53(p53)/Saos-p53-ChIP-Seq(GSE15780)/Homer	1e-4	-1.029e+01	0.0001	1071.0	0.63%	716.0	0.56%	motif file (matrix)	svg
308	p53(p53)/Saos-p53-ChIP-Seq/Homer	1e-4	-1.029e+01	0.0001	1071.0	0.63%	716.0	0.56%	motif file (matrix)	svg
309	VIP1(bZIP)/col-VIP1-DAP-Seq(GSE60143)/Homer	1e-4	-1.029e+01	0.0001	1944.0	1.15%	1343.2	1.05%	motif file (matrix)	svg
310	Foxo1(Forkhead)/RAW-Foxo1-ChIP-Seq(Fan_et_al.)/Homer	1e-4	-1.027e+01	0.0001	29789.0	17.62%	22079.8	17.25%	motif file (matrix)	svg
311	ETS:E-box(ETS,bHLH)/HPC7-Scl-ChIP-Seq(GSE22178)/Homer	1e-4	-1.005e+01	0.0001	845.0	0.50%	557.1	0.44%	motif file (matrix)	svg
312	ABF1(bZIP)/Arabidopsis-ABF1-ChIP-Seq(GSE80564)/Homer	1e-4	-1.004e+01	0.0001	8540.0	5.05%	6200.6	4.84%	motif file (matrix)	svg
313	RUNX2(Runt)/PCa-RUNX2-ChIP-Seq(GSE33889)/Homer	1e-4	-9.818e+00	0.0002	11330.0	6.70%	8279.0	6.47%	motif file (matrix)	svg
314	MYB3R4(MYB)/col-MYB3R4-DAP-Seq(GSE60143)/Homer	1e-4	-9.779e+00	0.0002	2676.0	1.58%	1879.1	1.47%	motif file (matrix)	svg
315	ANAC053(NAC)/colamp-ANAC053-DAP-Seq(GSE60143)/Homer	1e-4	-9.730e+00	0.0002	4427.0	2.62%	3163.9	2.47%	motif file (matrix)	svg
316	ZNF136(Zf)/HEK293-ZNF136.GFP-ChIP-Seq(GSE58341)/Homer	1e-4	-9.725e+00	0.0002	1388.0	0.82%	946.8	0.74%	motif file (matrix)	svg
317	Hoxa10(Homeobox)/ChickenMSG-Hoxa10.Flag-ChIP-Seq(GSE86088)/Homer	1e-4	-9.708e+00	0.0002	9619.0	5.69%	7007.9	5.48%	motif file (matrix)	svg
318	ANAC092(NAC)/colamp-ANAC092-DAP-Seq(GSE60143)/Homer	1e-4	-9.590e+00	0.0002	3957.0	2.34%	2819.1	2.20%	motif file (matrix)	svg
319	RAR:RXR(NR),DR5/ES-RAR-ChIP-Seq(GSE56893)/Homer	1e-4	-9.586e+00	0.0002	259.0	0.15%	154.0	0.12%	motif file (matrix)	svg
320	HDG1(Homeobox)/col100-HDG1-DAP-Seq(GSE60143)/Homer	1e-4	-9.564e+00	0.0002	13269.0	7.85%	9728.3	7.60%	motif file (matrix)	svg
321	AT2G01818(PLATZ)/col-AT2G01818-DAP-Seq(GSE60143)/Homer	1e-4	-9.289e+00	0.0003	1203.0	0.71%	816.6	0.64%	motif file (matrix)	svg
322	At3g11280(MYBrelated)/col-At3g11280-DAP-Seq(GSE60143)/Homer	1e-4	-9.284e+00	0.0003	17213.0	10.18%	12680.4	9.91%	motif file (matrix)	svg
323	CES-1(Homeobox)/cElegans-L1-CES1-ChIP-Seq(modEncode)/Homer	1e-4	-9.265e+00	0.0003	11571.0	6.84%	8468.1	6.62%	motif file (matrix)	svg
324	Bcl11a(Zf)/HSPC-BCL11A-ChIP-Seq(GSE104676)/Homer	1e-3	-9.207e+00	0.0003	9872.0	5.84%	7205.0	5.63%	motif file (matrix)	svg
325	ANAC070(NAC)/colamp-ANAC070-DAP-Seq(GSE60143)/Homer	1e-3	-9.192e+00	0.0003	15799.0	9.34%	11625.9	9.08%	motif file (matrix)	svg
326	n-Myc(bHLH)/mES-nMyc-ChIP-Seq(GSE11431)/Homer	1e-3	-9.137e+00	0.0003	8015.0	4.74%	5825.6	4.55%	motif file (matrix)	svg
327	p73(p53)/Trachea-p73-ChIP-Seq(PRJNA310161)/Homer	1e-3	-9.134e+00	0.0003	553.0	0.33%	356.3	0.28%	motif file (matrix)	svg
328	Tal1	1e-3	-9.050e+00	0.0004	19719.0	11.66%	14561.7	11.38%	motif file (matrix)	svg
329	EFL-1(E2F)/cElegans-L1-EFL1-ChIP-Seq(modEncode)/Homer	1e-3	-9.046e+00	0.0004	297.0	0.18%	180.1	0.14%	motif file (matrix)	svg
330	Bach1(bZIP)/K562-Bach1-ChIP-Seq(GSE31477)/Homer	1e-3	-8.947e+00	0.0004	982.0	0.58%	660.7	0.52%	motif file (matrix)	svg
331	REF6(Zf)/Arabidopsis-REF6-ChIP-Seq(GSE106942)/Homer	1e-3	-8.916e+00	0.0004	8749.0	5.17%	6374.5	4.98%	motif file (matrix)	svg
332	PU.1:IRF8(ETS:IRF)/pDC-Irf8-ChIP-Seq(GSE66899)/Homer	1e-3	-8.880e+00	0.0004	2079.0	1.23%	1452.3	1.13%	motif file (matrix)	svg
333	AtHB32(ZFHD)/col200-AtHB32-DAP-Seq(GSE60143)/Homer	1e-3	-8.879e+00	0.0004	32422.0	19.18%	24098.1	18.83%	motif file (matrix)	svg
334	HSF7(HSF)/colamp-HSF7-DAP-Seq(GSE60143)/Homer	1e-3	-8.870e+00	0.0004	1606.0	0.95%	1109.5	0.87%	motif file (matrix)	svg
335	Tcfcp2l1(CP2)/mES-Tcfcp2l1-ChIP-Seq(GSE11431)/Homer	1e-3	-8.588e+00	0.0006	1297.0	0.77%	888.4	0.69%	motif file (matrix)	svg
336	ANAC045(NAC)/col-ANAC045-DAP-Seq(GSE60143)/Homer	1e-3	-8.543e+00	0.0006	23393.0	13.84%	17328.8	13.54%	motif file (matrix)	svg
337	ZNF41(Zf)/HEK293-ZNF41.GFP-ChIP-Seq(GSE58341)/Homer	1e-3	-8.472e+00	0.0006	296.0	0.18%	181.8	0.14%	motif file (matrix)	svg
338	HOXD13(Homeobox)/Chicken-Hoxd13-ChIP-Seq(GSE38910)/Homer	1e-3	-8.444e+00	0.0006	23188.0	13.71%	17177.9	13.42%	motif file (matrix)	svg
339	Fli1(ETS)/CD8-FLI-ChIP-Seq(GSE20898)/Homer	1e-3	-8.243e+00	0.0008	16216.0	9.59%	11959.5	9.34%	motif file (matrix)	svg
340	AT3G09735(S1Falike)/col-AT3G09735-DAP-Seq(GSE60143)/Homer	1e-3	-8.232e+00	0.0008	4547.0	2.69%	3270.6	2.56%	motif file (matrix)	svg
341	PU.1-IRF(ETS:IRF)/Bcell-PU.1-ChIP-Seq(GSE21512)/Homer	1e-3	-8.216e+00	0.0008	15068.0	8.91%	11101.7	8.67%	motif file (matrix)	svg
342	ETV1(ETS)/GIST48-ETV1-ChIP-Seq(GSE22441)/Homer	1e-3	-8.074e+00	0.0009	18827.0	11.14%	13918.4	10.88%	motif file (matrix)	svg
343	Pax7(Paired,Homeobox),long/Myoblast-Pax7-ChIP-Seq(GSE25064)/Homer	1e-3	-8.033e+00	0.0010	485.0	0.29%	313.1	0.24%	motif file (matrix)	svg
344	ZFX(Zf)/mES-Zfx-ChIP-Seq(GSE11431)/Homer	1e-3	-8.025e+00	0.0010	11508.0	6.81%	8446.4	6.60%	motif file (matrix)	svg
345	LCL1(MYBrelated)/colamp-LCL1-DAP-Seq(GSE60143)/Homer	1e-3	-7.975e+00	0.0010	2308.0	1.37%	1627.3	1.27%	motif file (matrix)	svg
346	CUX1(Homeobox)/K562-CUX1-ChIP-Seq(GSE92882)/Homer	1e-3	-7.964e+00	0.0010	16422.0	9.71%	12121.0	9.47%	motif file (matrix)	svg
347	Replumless(BLH)/Arabidopsis-RPL.GFP-ChIP-Seq(GSE78727)/Homer	1e-3	-7.910e+00	0.0011	16169.0	9.56%	11932.3	9.32%	motif file (matrix)	svg
348	HSF21(HSF)/col-HSF21-DAP-Seq(GSE60143)/Homer	1e-3	-7.820e+00	0.0012	774.0	0.46%	518.9	0.41%	motif file (matrix)	svg
349	Unknown1(NR/Ini-like)/Drosophila-Promoters/Homer	1e-3	-7.629e+00	0.0014	2578.0	1.52%	1828.2	1.43%	motif file (matrix)	svg
350	ANAC013(NAC)/col-ANAC013-DAP-Seq(GSE60143)/Homer	1e-3	-7.514e+00	0.0016	3344.0	1.98%	2392.2	1.87%	motif file (matrix)	svg
351	SeqBias: GCW-triplet	1e-3	-7.504e+00	0.0016	167590.0	99.12%	126758.2	99.05%	motif file (matrix)	svg
352	LEF1(HMG)/H1-LEF1-ChIP-Seq(GSE64758)/Homer	1e-3	-7.465e+00	0.0016	9123.0	5.40%	6679.4	5.22%	motif file (matrix)	svg
353	GATA:SCL(Zf,bHLH)/Ter119-SCL-ChIP-Seq(GSE18720)/Homer	1e-3	-7.315e+00	0.0019	1544.0	0.91%	1076.9	0.84%	motif file (matrix)	svg
354	ZNF382(Zf)/HEK293-ZNF382.GFP-ChIP-Seq(GSE58341)/Homer	1e-3	-7.246e+00	0.0020	416.0	0.25%	268.7	0.21%	motif file (matrix)	svg
355	GEI-11(Myb?)/cElegans-L4-GEI11-ChIP-Seq(modEncode)/Homer	1e-3	-7.235e+00	0.0020	866.0	0.51%	587.7	0.46%	motif file (matrix)	svg
356	JGL(C2H2)/col-JGL-DAP-Seq(GSE60143)/Homer	1e-3	-7.126e+00	0.0023	30837.0	18.24%	22964.9	17.94%	motif file (matrix)	svg
357	Phox2a(Homeobox)/Neuron-Phox2a-ChIP-Seq(GSE31456)/Homer	1e-3	-7.062e+00	0.0024	7649.0	4.52%	5589.4	4.37%	motif file (matrix)	svg
358	GATA3(Zf),DR4/iTreg-Gata3-ChIP-Seq(GSE20898)/Homer	1e-3	-6.983e+00	0.0026	2109.0	1.25%	1491.6	1.17%	motif file (matrix)	svg
359	bHLHE40(bHLH)/HepG2-BHLHE40-ChIP-Seq(GSE31477)/Homer	1e-3	-6.944e+00	0.0027	3557.0	2.10%	2556.8	2.00%	motif file (matrix)	svg
360	GBF5(bZIP)/colamp-GBF5-DAP-Seq(GSE60143)/Homer	1e-3	-6.942e+00	0.0027	3055.0	1.81%	2186.4	1.71%	motif file (matrix)	svg
361	ANAC087(NAC)/col-ANAC087-DAP-Seq(GSE60143)/Homer	1e-2	-6.901e+00	0.0028	2558.0	1.51%	1821.7	1.42%	motif file (matrix)	svg
362	SFP1/SacCer-Promoters/Homer	1e-2	-6.831e+00	0.0030	1658.0	0.98%	1163.2	0.91%	motif file (matrix)	svg
363	VDR(NR),DR3/GM10855-VDR+vitD-ChIP-Seq(GSE22484)/Homer	1e-2	-6.787e+00	0.0031	3062.0	1.81%	2193.4	1.71%	motif file (matrix)	svg
364	ASHR1(ND)/col-ASHR1-DAP-Seq(GSE60143)/Homer	1e-2	-6.761e+00	0.0032	15889.0	9.40%	11753.0	9.18%	motif file (matrix)	svg
365	CEBP(bZIP)/ThioMac-CEBPb-ChIP-Seq(GSE21512)/Homer	1e-2	-6.701e+00	0.0034	13599.0	8.04%	10039.2	7.84%	motif file (matrix)	svg
366	AT1G04880(ARID)/colamp-AT1G04880-DAP-Seq(GSE60143)/Homer	1e-2	-6.664e+00	0.0035	4192.0	2.48%	3029.6	2.37%	motif file (matrix)	svg
367	MYB(HTH)/ERMYB-Myb-ChIPSeq(GSE22095)/Homer	1e-2	-6.649e+00	0.0036	38038.0	22.50%	28402.6	22.19%	motif file (matrix)	svg
368	FXR(NR),IR1/Liver-FXR-ChIP-Seq(Chong_et_al.)/Homer	1e-2	-6.587e+00	0.0038	5150.0	3.05%	3740.6	2.92%	motif file (matrix)	svg
369	NUC(C2H2)/col-NUC-DAP-Seq(GSE60143)/Homer	1e-2	-6.568e+00	0.0038	1259.0	0.74%	875.6	0.68%	motif file (matrix)	svg
370	AT2G20400(G2like)/colamp-AT2G20400-DAP-Seq(GSE60143)/Homer	1e-2	-6.501e+00	0.0041	2908.0	1.72%	2083.7	1.63%	motif file (matrix)	svg
371	Phox2b(Homeobox)/CLBGA-PHOX2B-ChIP-Seq(GSE90683)/Homer	1e-2	-6.484e+00	0.0041	4075.0	2.41%	2945.3	2.30%	motif file (matrix)	svg
372	IRF1(IRF)/PBMC-IRF1-ChIP-Seq(GSE43036)/Homer	1e-2	-6.455e+00	0.0043	1460.0	0.86%	1022.5	0.80%	motif file (matrix)	svg
373	AtIDD11(C2H2)/colamp-AtIDD11-DAP-Seq(GSE60143)/Homer	1e-2	-6.391e+00	0.0045	4709.0	2.79%	3417.0	2.67%	motif file (matrix)	svg
374	LBD2(LOBAS2)/colamp-LBD2-DAP-Seq(GSE60143)/Homer	1e-2	-6.300e+00	0.0049	3962.0	2.34%	2864.5	2.24%	motif file (matrix)	svg
375	EWS:ERG-fusion(ETS)/CADO_ES1-EWS:ERG-ChIP-Seq(SRA014231)/Homer	1e-2	-6.146e+00	0.0057	10262.0	6.07%	7557.1	5.90%	motif file (matrix)	svg
376	AARE(HLH)/mES-cMyc-ChIP-Seq/Homer	1e-2	-6.111e+00	0.0059	1221.0	0.72%	851.7	0.67%	motif file (matrix)	svg
377	VND3(NAC)/colamp-VND3-DAP-Seq(GSE60143)/Homer	1e-2	-6.098e+00	0.0060	7412.0	4.38%	5431.3	4.24%	motif file (matrix)	svg
378	AT1G10720(BSD)/col-AT1G10720-DAP-Seq(GSE60143)/Homer	1e-2	-5.954e+00	0.0069	130.0	0.08%	76.5	0.06%	motif file (matrix)	svg
379	Esrrb(NR)/mES-Esrrb-ChIP-Seq(GSE11431)/Homer	1e-2	-5.942e+00	0.0070	7554.0	4.47%	5540.5	4.33%	motif file (matrix)	svg
380	TEAD1(TEAD)/HepG2-TEAD1-ChIP-Seq(Encode)/Homer	1e-2	-5.779e+00	0.0082	13867.0	8.20%	10264.7	8.02%	motif file (matrix)	svg
381	OBP3(C2C2dof)/col-OBP3-DAP-Seq(GSE60143)/Homer	1e-2	-5.772e+00	0.0082	64534.0	38.17%	48435.6	37.85%	motif file (matrix)	svg
382	bZIP69(bZIP)/col-bZIP69-DAP-Seq(GSE60143)/Homer	1e-2	-5.744e+00	0.0084	1049.0	0.62%	730.0	0.57%	motif file (matrix)	svg
383	SND3(NAC)/col-SND3-DAP-Seq(GSE60143)/Homer	1e-2	-5.742e+00	0.0084	8370.0	4.95%	6153.6	4.81%	motif file (matrix)	svg
384	ANAC057(NAC)/colamp-ANAC057-DAP-Seq(GSE60143)/Homer	1e-2	-5.734e+00	0.0085	7327.0	4.33%	5375.8	4.20%	motif file (matrix)	svg
385	GFY(?)/Promoter/Homer	1e-2	-5.606e+00	0.0096	1551.0	0.92%	1096.2	0.86%	motif file (matrix)	svg
386	At5g05790(MYBrelated)/col-At5g05790-DAP-Seq(GSE60143)/Homer	1e-2	-5.604e+00	0.0096	18200.0	10.76%	13520.0	10.56%	motif file (matrix)	svg
387	bZIP16(bZIP)/colamp-bZIP16-DAP-Seq(GSE60143)/Homer	1e-2	-5.526e+00	0.0103	2611.0	1.54%	1876.4	1.47%	motif file (matrix)	svg
388	MYB94(MYB)/col-MYB94-DAP-Seq(GSE60143)/Homer	1e-2	-5.520e+00	0.0104	5087.0	3.01%	3711.3	2.90%	motif file (matrix)	svg
389	At5g62940(C2C2dof)/col-At5g62940-DAP-Seq(GSE60143)/Homer	1e-2	-5.481e+00	0.0108	68645.0	40.60%	51557.4	40.29%	motif file (matrix)	svg
390	MafB(bZIP)/BMM-Mafb-ChIP-Seq(GSE75722)/Homer	1e-2	-5.425e+00	0.0114	5234.0	3.10%	3822.3	2.99%	motif file (matrix)	svg
391	RORgt(NR)/EL4-RORgt.Flag-ChIP-Seq(GSE56019)/Homer	1e-2	-5.395e+00	0.0116	1339.0	0.79%	943.6	0.74%	motif file (matrix)	svg
392	RORgt(NR)/EL4-RORgt.Flag-ChIP-Seq(GSE56019)/Homer	1e-2	-5.395e+00	0.0116	1339.0	0.79%	943.6	0.74%	motif file (matrix)	svg
393	bZIP68(bZIP)/col-bZIP68-DAP-Seq(GSE60143)/Homer	1e-2	-5.348e+00	0.0122	3237.0	1.91%	2341.8	1.83%	motif file (matrix)	svg
394	DREF/Drosophila-Promoters/Homer	1e-2	-5.314e+00	0.0126	279.0	0.17%	181.0	0.14%	motif file (matrix)	svg
395	AGL15(MADS)/col-AGL15-DAP-Seq(GSE60143)/Homer	1e-2	-5.293e+00	0.0128	3140.0	1.86%	2270.7	1.77%	motif file (matrix)	svg
396	NRF(NRF)/Promoter/Homer	1e-2	-5.233e+00	0.0136	3525.0	2.08%	2556.6	2.00%	motif file (matrix)	svg
397	bZIP3(bZIP)/col-bZIP3-DAP-Seq(GSE60143)/Homer	1e-2	-5.195e+00	0.0141	5634.0	3.33%	4124.6	3.22%	motif file (matrix)	svg
398	GFY-Staf(?,Zf)/Promoter/Homer	1e-2	-5.135e+00	0.0149	1400.0	0.83%	990.2	0.77%	motif file (matrix)	svg
399	SeqBias: CG-repeat	1e-2	-5.083e+00	0.0156	12050.0	7.13%	8922.9	6.97%	motif file (matrix)	svg
400	IRF2(IRF)/Erythroblas-IRF2-ChIP-Seq(GSE36985)/Homer	1e-2	-5.083e+00	0.0156	1253.0	0.74%	883.0	0.69%	motif file (matrix)	svg
401	SPL3(SBP)/colamp-SPL3-DAP-Seq(GSE60143)/Homer	1e-2	-4.988e+00	0.0171	703.0	0.42%	484.4	0.38%	motif file (matrix)	svg
402	TEAD4(TEA)/Tropoblast-Tead4-ChIP-Seq(GSE37350)/Homer	1e-2	-4.981e+00	0.0172	11894.0	7.03%	8808.6	6.88%	motif file (matrix)	svg
403	MNT(bHLH)/HepG2-MNT-ChIP-Seq(Encode)/Homer	1e-2	-4.854e+00	0.0195	17171.0	10.16%	12771.1	9.98%	motif file (matrix)	svg
404	ANAC050(NAC)/colamp-ANAC050-DAP-Seq(GSE60143)/Homer	1e-2	-4.838e+00	0.0197	4842.0	2.86%	3542.0	2.77%	motif file (matrix)	svg
405	bHLH34(bHLH)/colamp-bHLH34-DAP-Seq(GSE60143)/Homer	1e-2	-4.791e+00	0.0206	3145.0	1.86%	2281.1	1.78%	motif file (matrix)	svg
406	CUC1(NAC)/col-CUC1-DAP-Seq(GSE60143)/Homer	1e-2	-4.747e+00	0.0215	4013.0	2.37%	2926.1	2.29%	motif file (matrix)	svg
407	RIN(MADS)/Tomato-RIN-ChIP-Seq(GSE116581)/Homer	1e-2	-4.687e+00	0.0228	25234.0	14.92%	18840.7	14.72%	motif file (matrix)	svg
408	RORa(NR)/Liver-Rora-ChIP-Seq(GSE101115)/Homer	1e-2	-4.608e+00	0.0246	1691.0	1.00%	1209.9	0.95%	motif file (matrix)	svg

Homer Known Motif Enrichment Results (allNoMT_Common_txt_motif)

Maintained Peaks after 3pmol Foxi1MO

Homer *de novo* Motif Results
Gene Ontology Enrichment Results
Known Motif Enrichment Results (txt file)
Total Target Sequences = 142380, Total Background Sequences = 153626

Rank	Motif	Name	P-value	log P-pvalue	q-value (Benjamini)	# Target Sequences with Motif	% of Targets Sequences with Motif	# Background Sequences with Motif	% of Background Sequences with Motif	Motif File	SVG
1		GATA6(C2C2gata)/col200-GATA6-DAP-Seq(GSE60143)/Homer	1e-278	-6.403e+02	0.0000	5923.0	4.16%	3902.1	2.54%	motif file (matrix)	svg
2		E-box/Drosophila-Promoters/Homer	1e-227	-5.238e+02	0.0000	6988.0	4.91%	5039.0	3.28%	motif file (matrix)	svg
3		PRDM14(Zf)/H1-PRDM14-ChIP-Seq(GSE22767)/Homer	1e-211	-4.868e+02	0.0000	9833.0	6.91%	7703.5	5.02%	motif file (matrix)	svg
4		Brachyury(T-box)/Mesoendoderm-Brachyury-ChIP-exo(GSE54963)/Homer	1e-189	-4.366e+02	0.0000	6976.0	4.90%	5222.0	3.40%	motif file (matrix)	svg
5		Reverb(NR),DR2/RAW-Reverba.biotin-ChIP-Seq(GSE45914)/Homer	1e-180	-4.166e+02	0.0000	3737.0	2.63%	2436.5	1.59%	motif file (matrix)	svg
6		Sox7(HMG)/ESC-Sox7-ChIP-Seq(GSE133899)/Homer	1e-177	-4.095e+02	0.0000	9794.0	6.89%	7887.7	5.14%	motif file (matrix)	svg
7		Gata1(Zf)/K562-GATA1-ChIP-Seq(GSE18829)/Homer	1e-174	-4.024e+02	0.0000	14799.0	10.40%	12695.9	8.27%	motif file (matrix)	svg
8		PRDM9(Zf)/Testis-DMC1-ChIP-Seq(GSE35498)/Homer	1e-170	-3.929e+02	0.0000	10024.0	7.05%	8154.9	5.31%	motif file (matrix)	svg
9		ERF105(AP2EREBP)/colamp-ERF105-DAP-Seq(GSE60143)/Homer	1e-168	-3.871e+02	0.0000	12431.0	8.74%	10464.4	6.82%	motif file (matrix)	svg
10		ERF13(AP2EREBP)/colamp-ERF13-DAP-Seq(GSE60143)/Homer	1e-158	-3.650e+02	0.0000	13336.0	9.38%	11419.9	7.44%	motif file (matrix)	svg
11		Gata2(Zf)/K562-GATA2-ChIP-Seq(GSE18829)/Homer	1e-149	-3.452e+02	0.0000	15633.0	10.99%	13742.6	8.95%	motif file (matrix)	svg
12		AT5G23930(mTERF)/col-AT5G23930-DAP-Seq(GSE60143)/Homer	1e-142	-3.274e+02	0.0000	17107.0	12.03%	15277.3	9.95%	motif file (matrix)	svg
13		Atf2(bZIP)/3T3L1-Atf2-ChIP-Seq(GSE56872)/Homer	1e-128	-2.962e+02	0.0000	9429.0	6.63%	7911.6	5.15%	motif file (matrix)	svg
14		GATA14(C2C2gata)/col-GATA14-DAP-Seq(GSE60143)/Homer	1e-128	-2.958e+02	0.0000	13487.0	9.48%	11848.2	7.72%	motif file (matrix)	svg
15		c-Jun-CRE(bZIP)/K562-cJun-ChIP-Seq(GSE31477)/Homer	1e-127	-2.939e+02	0.0000	9014.0	6.34%	7524.0	4.90%	motif file (matrix)	svg
16		Sox4(HMG)/proB-Sox4-ChIP-Seq(GSE50066)/Homer	1e-124	-2.863e+02	0.0000	15059.0	10.59%	13440.1	8.75%	motif file (matrix)	svg
17		ERF15(AP2EREBP)/colamp-ERF15-DAP-Seq(GSE60143)/Homer	1e-124	-2.860e+02	0.0000	26620.0	18.72%	25094.2	16.34%	motif file (matrix)	svg
18		Gata4(Zf)/Heart-Gata4-ChIP-Seq(GSE35151)/Homer	1e-115	-2.650e+02	0.0000	20899.0	14.69%	19402.0	12.64%	motif file (matrix)	svg
19		Sox15(HMG)/CPA-Sox15-ChIP-Seq(GSE62909)/Homer	1e-114	-2.626e+02	0.0000	20466.0	14.39%	18977.3	12.36%	motif file (matrix)	svg
20		MyoD(bHLH)/Myotube-MyoD-ChIP-Seq(GSE21614)/Homer	1e-112	-2.591e+02	0.0000	13879.0	9.76%	12401.7	8.08%	motif file (matrix)	svg
21		GATA11(C2C2gata)/col-GATA11-DAP-Seq(GSE60143)/Homer	1e-110	-2.536e+02	0.0000	8827.0	6.21%	7491.0	4.88%	motif file (matrix)	svg
22		Gata6(Zf)/HUG1N-GATA6-ChIP-Seq(GSE51936)/Homer	1e-104	-2.416e+02	0.0000	19329.0	13.59%	17949.8	11.69%	motif file (matrix)	svg
23		Sox2(HMG)/mES-Sox2-ChIP-Seq(GSE11431)/Homer	1e-103	-2.394e+02	0.0000	17439.0	12.26%	16056.5	10.46%	motif file (matrix)	svg
24		ZBTB18(Zf)/HEK293-ZBTB18.GFP-ChIP-Seq(GSE58341)/Homer	1e-101	-2.335e+02	0.0000	8177.0	5.75%	6941.5	4.52%	motif file (matrix)	svg
25		Atf7(bZIP)/3T3L1-Atf7-ChIP-Seq(GSE56872)/Homer	1e-101	-2.334e+02	0.0000	10933.0	7.69%	9618.2	6.26%	motif file (matrix)	svg
26		Sox17(HMG)/Endoderm-Sox17-ChIP-Seq(GSE61475)/Homer	1e-98	-2.272e+02	0.0000	17099.0	12.02%	15783.1	10.28%	motif file (matrix)	svg
27		Rfx6(HTH)/Min6b1-Rfx6.HA-ChIP-Seq(GSE62844)/Homer	1e-96	-2.229e+02	0.0000	17736.0	12.47%	16450.9	10.71%	motif file (matrix)	svg
28		EMB1789(C3H)/col-EMB1789-DAP-Seq(GSE60143)/Homer	1e-95	-2.189e+02	0.0000	10418.0	7.32%	9178.2	5.98%	motif file (matrix)	svg
29		Egr1(Zf)/K562-Egr1-ChIP-Seq(GSE32465)/Homer	1e-94	-2.165e+02	0.0000	8737.0	6.14%	7549.5	4.92%	motif file (matrix)	svg
30		FEA4(bZIP)/Corn-FEA4-ChIP-Seq(GSE61954)/Homer	1e-93	-2.152e+02	0.0000	19624.0	13.80%	18408.9	11.99%	motif file (matrix)	svg
31		CREB5(bZIP)/LNCaP-CREB5.V5-ChIP-Seq(GSE137775)/Homer	1e-91	-2.116e+02	0.0000	10014.0	7.04%	8815.6	5.74%	motif file (matrix)	svg
32		E2F3(E2F)/MEF-E2F3-ChIP-Seq(GSE71376)/Homer	1e-86	-1.997e+02	0.0000	6805.0	4.78%	5752.2	3.75%	motif file (matrix)	svg
33		Maz(Zf)/HepG2-Maz-ChIP-Seq(GSE31477)/Homer	1e-85	-1.961e+02	0.0000	20156.0	14.17%	19074.4	12.42%	motif file (matrix)	svg
34		Tcf12(bHLH)/GM12878-Tcf12-ChIP-Seq(GSE32465)/Homer	1e-83	-1.927e+02	0.0000	16185.0	11.38%	15064.9	9.81%	motif file (matrix)	svg
35		Myf5(bHLH)/GM-Myf5-ChIP-Seq(GSE24852)/Homer	1e-83	-1.926e+02	0.0000	11156.0	7.84%	10030.6	6.53%	motif file (matrix)	svg
36		At2g33710(AP2EREBP)/colamp-At2g33710-DAP-Seq(GSE60143)/Homer	1e-82	-1.901e+02	0.0000	19330.0	13.59%	18272.5	11.90%	motif file (matrix)	svg
37		TFE3(bHLH)/MEF-TFE3-ChIP-Seq(GSE75757)/Homer	1e-79	-1.827e+02	0.0000	5158.0	3.63%	4246.9	2.77%	motif file (matrix)	svg
38		OCT4-SOX2-TCF-NANOG(POU,Homeobox,HMG)/mES-Oct4-ChIP-Seq(GSE11431)/Homer	1e-77	-1.778e+02	0.0000	4750.0	3.34%	3878.5	2.53%	motif file (matrix)	svg
39		PAX5(Paired,Homeobox),condensed/GM12878-PAX5-ChIP-Seq(GSE32465)/Homer	1e-76	-1.753e+02	0.0000	1692.0	1.19%	1125.5	0.73%	motif file (matrix)	svg
40		KLF5(Zf)/LoVo-KLF5-ChIP-Seq(GSE49402)/Homer	1e-76	-1.751e+02	0.0000	13483.0	9.48%	12447.6	8.11%	motif file (matrix)	svg
41		Sox3(HMG)/NPC-Sox3-ChIP-Seq(GSE33059)/Homer	1e-75	-1.747e+02	0.0000	30994.0	21.79%	30421.5	19.81%	motif file (matrix)	svg
42		Atf1(bZIP)/K562-ATF1-ChIP-Seq(GSE31477)/Homer	1e-70	-1.624e+02	0.0000	13640.0	9.59%	12681.9	8.26%	motif file (matrix)	svg
43		Tcf21(bHLH)/ArterySmoothMuscle-Tcf21-ChIP-Seq(GSE61369)/Homer	1e-68	-1.575e+02	0.0000	14533.0	10.22%	13614.1	8.87%	motif file (matrix)	svg
44		HLH-1(bHLH)/cElegans-Embryo-HLH1-ChIP-Seq(modEncode)/Homer	1e-67	-1.560e+02	0.0000	13537.0	9.52%	12617.8	8.22%	motif file (matrix)	svg
45		GATA12(C2C2gata)/col-GATA12-DAP-Seq(GSE60143)/Homer	1e-67	-1.547e+02	0.0000	10882.0	7.65%	9959.0	6.49%	motif file (matrix)	svg
46		Sp5(Zf)/mES-Sp5.Flag-ChIP-Seq(GSE72989)/Homer	1e-66	-1.542e+02	0.0000	10875.0	7.65%	9954.3	6.48%	motif file (matrix)	svg
47		Usf2(bHLH)/C2C12-Usf2-ChIP-Seq(GSE36030)/Homer	1e-66	-1.525e+02	0.0000	7720.0	5.43%	6837.6	4.45%	motif file (matrix)	svg
48		MyoG(bHLH)/C2C12-MyoG-ChIP-Seq(GSE36024)/Homer	1e-65	-1.503e+02	0.0000	15288.0	10.75%	14427.4	9.40%	motif file (matrix)	svg
49		Sox10(HMG)/SciaticNerve-Sox3-ChIP-Seq(GSE35132)/Homer	1e-61	-1.422e+02	0.0000	26900.0	18.91%	26447.8	17.23%	motif file (matrix)	svg
50		Oct6(POU,Homeobox)/NPC-Pou3f1-ChIP-Seq(GSE35496)/Homer	1e-60	-1.392e+02	0.0000	8112.0	5.70%	7288.4	4.75%	motif file (matrix)	svg
51		KLF1(Zf)/HUDEP2-KLF1-CutnRun(GSE136251)/Homer	1e-60	-1.382e+02	0.0000	9351.0	6.57%	8523.6	5.55%	motif file (matrix)	svg
52		At1g49010(MYBrelated)/col-At1g49010-DAP-Seq(GSE60143)/Homer	1e-58	-1.343e+02	0.0000	37338.0	26.25%	37457.0	24.40%	motif file (matrix)	svg
53		KLF14(Zf)/HEK293-KLF14.GFP-ChIP-Seq(GSE58341)/Homer	1e-57	-1.334e+02	0.0000	19564.0	13.75%	18928.3	12.33%	motif file (matrix)	svg
54		Sp1(Zf)/Promoter/Homer	1e-57	-1.314e+02	0.0000	2746.0	1.93%	2155.3	1.40%	motif file (matrix)	svg
55		AT1G76880(Trihelix)/col-AT1G76880-DAP-Seq(GSE60143)/Homer	1e-55	-1.279e+02	0.0000	9452.0	6.65%	8682.8	5.66%	motif file (matrix)	svg
56		Oct4(POU,Homeobox)/mES-Oct4-ChIP-Seq(GSE11431)/Homer	1e-53	-1.236e+02	0.0000	9342.0	6.57%	8597.8	5.60%	motif file (matrix)	svg
57		Sox21(HMG)/ESC-SOX21-ChIP-Seq(GSE110505)/Homer	1e-53	-1.221e+02	0.0000	29460.0	20.71%	29312.9	19.09%	motif file (matrix)	svg
58		BORIS(Zf)/K562-CTCFL-ChIP-Seq(GSE32465)/Homer	1e-52	-1.198e+02	0.0000	4072.0	2.86%	3438.6	2.24%	motif file (matrix)	svg
59		GATA3(Zf)/iTreg-Gata3-ChIP-Seq(GSE20898)/Homer	1e-51	-1.191e+02	0.0000	29722.0	20.90%	29618.3	19.29%	motif file (matrix)	svg
60		Six2(Homeobox)/NephronProgenitor-Six2-ChIP-Seq(GSE39837)/Homer	1e-50	-1.173e+02	0.0000	18392.0	12.93%	17856.0	11.63%	motif file (matrix)	svg
61		Cbf1(bHLH)/Yeast-Cbf1-ChIP-Seq(GSE29506)/Homer	1e-50	-1.163e+02	0.0000	6888.0	4.84%	6196.4	4.04%	motif file (matrix)	svg
62		Atoh1(bHLH)/Cerebellum-Atoh1-ChIP-Seq(GSE22111)/Homer	1e-50	-1.160e+02	0.0000	16042.0	11.28%	15450.0	10.06%	motif file (matrix)	svg
63		Klf9(Zf)/GBM-Klf9-ChIP-Seq(GSE62211)/Homer	1e-49	-1.135e+02	0.0000	4505.0	3.17%	3879.9	2.53%	motif file (matrix)	svg
64		AT3G57600(AP2EREBP)/col-AT3G57600-DAP-Seq(GSE60143)/Homer	1e-48	-1.126e+02	0.0000	4737.0	3.33%	4107.0	2.67%	motif file (matrix)	svg
65		KLF3(Zf)/MEF-Klf3-ChIP-Seq(GSE44748)/Homer	1e-48	-1.125e+02	0.0000	4944.0	3.48%	4308.8	2.81%	motif file (matrix)	svg
66		Sp2(Zf)/HEK293-Sp2.eGFP-ChIP-Seq(Encode)/Homer	1e-44	-1.028e+02	0.0000	15984.0	11.24%	15498.7	10.09%	motif file (matrix)	svg
67		Ap4(bHLH)/AML-Tfap4-ChIP-Seq(GSE45738)/Homer	1e-44	-1.019e+02	0.0000	16994.0	11.95%	16546.8	10.78%	motif file (matrix)	svg
68		USF1(bHLH)/GM12878-Usf1-ChIP-Seq(GSE32465)/Homer	1e-44	-1.019e+02	0.0000	8783.0	6.17%	8169.0	5.32%	motif file (matrix)	svg
69		At5g58900(MYBrelated)/colamp-At5g58900-DAP-Seq(GSE60143)/Homer	1e-43	-9.923e+01	0.0000	30460.0	21.41%	30611.0	19.94%	motif file (matrix)	svg
70		MYB113(MYB)/col-MYB113-DAP-Seq(GSE60143)/Homer	1e-42	-9.858e+01	0.0000	16135.0	11.34%	15690.7	10.22%	motif file (matrix)	svg
71		MYB98(MYB)/col-MYB98-DAP-Seq(GSE60143)/Homer	1e-42	-9.844e+01	0.0000	7453.0	5.24%	6856.3	4.47%	motif file (matrix)	svg
72		Brn1(POU,Homeobox)/NPC-Brn1-ChIP-Seq(GSE35496)/Homer	1e-41	-9.614e+01	0.0000	6030.0	4.24%	5453.6	3.55%	motif file (matrix)	svg
73		GATA4(C2C2gata)/col-GATA4-DAP-Seq(GSE60143)/Homer	1e-40	-9.437e+01	0.0000	13373.0	9.40%	12887.8	8.39%	motif file (matrix)	svg
74		DMRT6(DM)/Testis-DMRT6-ChIP-Seq(GSE60440)/Homer	1e-40	-9.385e+01	0.0000	5759.0	4.05%	5197.3	3.38%	motif file (matrix)	svg
75		Zic(Zf)/Cerebellum-ZIC1.2-ChIP-Seq(GSE60731)/Homer	1e-40	-9.316e+01	0.0000	12765.0	8.97%	12274.5	7.99%	motif file (matrix)	svg
76		CTCF(Zf)/CD4+-CTCF-ChIP-Seq(Barski_et_al.)/Homer	1e-39	-9.063e+01	0.0000	3984.0	2.80%	3472.1	2.26%	motif file (matrix)	svg
77		Sox9(HMG)/Limb-SOX9-ChIP-Seq(GSE73225)/Homer	1e-36	-8.398e+01	0.0000	14530.0	10.22%	14166.7	9.23%	motif file (matrix)	svg
78		KLF6(Zf)/PDAC-KLF6-ChIP-Seq(GSE64557)/Homer	1e-36	-8.333e+01	0.0000	10620.0	7.47%	10160.3	6.62%	motif file (matrix)	svg
79		Klf4(Zf)/mES-Klf4-ChIP-Seq(GSE11431)/Homer	1e-32	-7.587e+01	0.0000	3900.0	2.74%	3458.8	2.25%	motif file (matrix)	svg
80		ZNF416(Zf)/HEK293-ZNF416.GFP-ChIP-Seq(GSE58341)/Homer	1e-32	-7.523e+01	0.0000	14739.0	10.36%	14464.8	9.42%	motif file (matrix)	svg
81		TGA2(bZIP)/colamp-TGA2-DAP-Seq(GSE60143)/Homer	1e-32	-7.486e+01	0.0000	8325.0	5.85%	7889.9	5.14%	motif file (matrix)	svg
82		bHLH130(bHLH)/col-bHLH130-DAP-Seq(GSE60143)/Homer	1e-32	-7.480e+01	0.0000	9967.0	7.01%	9561.6	6.23%	motif file (matrix)	svg
83		MYB65(MYB)/colamp-MYB65-DAP-Seq(GSE60143)/Homer	1e-29	-6.711e+01	0.0000	27798.0	19.54%	28212.5	18.37%	motif file (matrix)	svg
84		Ascl1(bHLH)/NeuralTubes-Ascl1-ChIP-Seq(GSE55840)/Homer	1e-29	-6.708e+01	0.0000	19748.0	13.88%	19761.8	12.87%	motif file (matrix)	svg
85		ZNF467(Zf)/HEK293-ZNF467.GFP-ChIP-Seq(GSE58341)/Homer	1e-28	-6.646e+01	0.0000	11679.0	8.21%	11389.1	7.42%	motif file (matrix)	svg
86		BHLHA15(bHLH)/NIH3T3-BHLHB8.HA-ChIP-Seq(GSE119782)/Homer	1e-28	-6.619e+01	0.0000	20434.0	14.37%	20489.1	13.34%	motif file (matrix)	svg
87		CLOCK(bHLH)/Liver-Clock-ChIP-Seq(GSE39860)/Homer	1e-28	-6.542e+01	0.0000	9255.0	6.51%	8910.8	5.80%	motif file (matrix)	svg
88		NFY(CCAAT)/Promoter/Homer	1e-27	-6.426e+01	0.0000	17865.0	12.56%	17828.2	11.61%	motif file (matrix)	svg
89		Ascl2(bHLH)/ESC-Ascl2-ChIP-Seq(GSE97712)/Homer	1e-27	-6.415e+01	0.0000	15914.0	11.19%	15797.9	10.29%	motif file (matrix)	svg
90		M1BP(Zf)/S2R+-M1BP-ChIP-Seq(GSE49842)/Homer	1e-27	-6.236e+01	0.0000	3247.0	2.28%	2884.5	1.88%	motif file (matrix)	svg
91		Sox6(HMG)/Myotubes-Sox6-ChIP-Seq(GSE32627)/Homer	1e-26	-6.125e+01	0.0000	27808.0	19.55%	28305.8	18.44%	motif file (matrix)	svg
92		TRPS1(Zf)/MCF7-TRPS1-ChIP-Seq(GSE107013)/Homer	1e-25	-5.940e+01	0.0000	37149.0	26.12%	38226.7	24.90%	motif file (matrix)	svg
93		CArG(MADS)/PUER-Srf-ChIP-Seq(Sullivan_et_al.)/Homer	1e-25	-5.901e+01	0.0000	7046.0	4.95%	6709.3	4.37%	motif file (matrix)	svg
94		CRE(bZIP)/Promoter/Homer	1e-24	-5.710e+01	0.0000	5522.0	3.88%	5180.6	3.37%	motif file (matrix)	svg
95		TCF4(bHLH)/SHSY5Y-TCF4-ChIP-Seq(GSE96915)/Homer	1e-24	-5.702e+01	0.0000	22030.0	15.49%	22280.3	14.51%	motif file (matrix)	svg
96		DMRT1(DM)/Testis-DMRT1-ChIP-Seq(GSE64892)/Homer	1e-24	-5.570e+01	0.0000	6394.0	4.50%	6072.6	3.96%	motif file (matrix)	svg
97		MYB62(MYB)/colamp-MYB62-DAP-Seq(GSE60143)/Homer	1e-21	-5.018e+01	0.0000	22765.0	16.00%	23150.2	15.08%	motif file (matrix)	svg
98		bZIP52(bZIP)/colamp-bZIP52-DAP-Seq(GSE60143)/Homer	1e-21	-4.908e+01	0.0000	19860.0	13.96%	20110.7	13.10%	motif file (matrix)	svg
99		Bapx1(Homeobox)/VertebralCol-Bapx1-ChIP-Seq(GSE36672)/Homer	1e-21	-4.896e+01	0.0000	30471.0	21.42%	31313.0	20.39%	motif file (matrix)	svg
100		E-box(bHLH)/Promoter/Homer	1e-21	-4.870e+01	0.0000	1903.0	1.34%	1639.0	1.07%	motif file (matrix)	svg
101		RAP211(AP2EREBP)/colamp-RAP211-DAP-Seq(GSE60143)/Homer	1e-19	-4.524e+01	0.0000	20700.0	14.55%	21049.3	13.71%	motif file (matrix)	svg
102		SUT1?/SacCer-Promoters/Homer	1e-18	-4.329e+01	0.0000	73742.0	51.84%	77778.4	50.66%	motif file (matrix)	svg
103		BOS1(MYB)/col-BOS1-DAP-Seq(GSE60143)/Homer	1e-18	-4.268e+01	0.0000	15557.0	10.94%	15685.6	10.22%	motif file (matrix)	svg
104		ERF104(AP2EREBP)/col-ERF104-DAP-Seq(GSE60143)/Homer	1e-17	-4.116e+01	0.0000	4929.0	3.47%	4696.6	3.06%	motif file (matrix)	svg
105		E2A(bHLH)/proBcell-E2A-ChIP-Seq(GSE21978)/Homer	1e-17	-4.077e+01	0.0000	18882.0	13.27%	19203.3	12.51%	motif file (matrix)	svg
106		HY5(bZIP)/colamp-HY5-DAP-Seq(GSE60143)/Homer	1e-17	-4.063e+01	0.0000	9576.0	6.73%	9476.3	6.17%	motif file (matrix)	svg
107		ERF2(AP2EREBP)/colamp-ERF2-DAP-Seq(GSE60143)/Homer	1e-15	-3.640e+01	0.0000	2999.0	2.11%	2782.9	1.81%	motif file (matrix)	svg
108		AT4G18450(AP2EREBP)/col-AT4G18450-DAP-Seq(GSE60143)/Homer	1e-15	-3.596e+01	0.0000	2018.0	1.42%	1810.9	1.18%	motif file (matrix)	svg
109		PIF5ox(bHLH)/Arabidopsis-PIF5ox-ChIP-Seq(GSE35062)/Homer	1e-15	-3.596e+01	0.0000	16944.0	11.91%	17238.2	11.23%	motif file (matrix)	svg
110		KLF10(Zf)/HEK293-KLF10.GFP-ChIP-Seq(GSE58341)/Homer	1e-15	-3.512e+01	0.0000	6807.0	4.79%	6673.0	4.35%	motif file (matrix)	svg
111		ERF1(AP2EREBP)/colamp-ERF1-DAP-Seq(GSE60143)/Homer	1e-15	-3.480e+01	0.0000	2765.0	1.94%	2558.5	1.67%	motif file (matrix)	svg
112		ESE1(AP2EREBP)/col-ESE1-DAP-Seq(GSE60143)/Homer	1e-14	-3.353e+01	0.0000	3208.0	2.26%	3011.0	1.96%	motif file (matrix)	svg
113		NFkB-p50,p52(RHD)/Monocyte-p50-ChIP-Chip(Schreiber_et_al.)/Homer	1e-14	-3.329e+01	0.0000	1625.0	1.14%	1438.4	0.94%	motif file (matrix)	svg
114		Egr2(Zf)/Thymocytes-Egr2-ChIP-Seq(GSE34254)/Homer	1e-14	-3.227e+01	0.0000	1457.0	1.02%	1279.7	0.83%	motif file (matrix)	svg
115		E2F6(E2F)/Hela-E2F6-ChIP-Seq(GSE31477)/Homer	1e-13	-3.177e+01	0.0000	3812.0	2.68%	3635.8	2.37%	motif file (matrix)	svg
116		MYB56(MYB)/colamp-MYB56-DAP-Seq(GSE60143)/Homer	1e-13	-3.159e+01	0.0000	16079.0	11.30%	16398.0	10.68%	motif file (matrix)	svg
117		Twist2(bHLH)/Myoblast-Twist2.Ty1-ChIP-Seq(GSE127998)/Homer	1e-13	-3.081e+01	0.0000	24331.0	17.11%	25133.1	16.37%	motif file (matrix)	svg
118		Oct2(POU,Homeobox)/Bcell-Oct2-ChIP-Seq(GSE21512)/Homer	1e-13	-3.078e+01	0.0000	5228.0	3.68%	5090.5	3.32%	motif file (matrix)	svg
119		bHLH122(bHLH)/col100-bHLH122-DAP-Seq(GSE60143)/Homer	1e-13	-3.077e+01	0.0000	11297.0	7.94%	11390.8	7.42%	motif file (matrix)	svg
120		Pax7(Paired,Homeobox),longest/Myoblast-Pax7-ChIP-Seq(GSE25064)/Homer	1e-13	-3.010e+01	0.0000	562.0	0.40%	437.3	0.28%	motif file (matrix)	svg
121		EKLF(Zf)/Erythrocyte-Klf1-ChIP-Seq(GSE20478)/Homer	1e-12	-2.988e+01	0.0000	2300.0	1.62%	2124.5	1.38%	motif file (matrix)	svg
122		DREB19(AP2EREBP)/colamp-DREB19-DAP-Seq(GSE60143)/Homer	1e-12	-2.965e+01	0.0000	4489.0	3.16%	4343.0	2.83%	motif file (matrix)	svg
123		Smad3(MAD)/NPC-Smad3-ChIP-Seq(GSE36673)/Homer	1e-12	-2.918e+01	0.0000	39112.0	27.50%	40911.9	26.65%	motif file (matrix)	svg
124		NeuroG2(bHLH)/Fibroblast-NeuroG2-ChIP-Seq(GSE75910)/Homer	1e-12	-2.882e+01	0.0000	21795.0	15.32%	22484.7	14.64%	motif file (matrix)	svg
125		Rfx5(HTH)/GM12878-Rfx5-ChIP-Seq(GSE31477)/Homer	1e-12	-2.881e+01	0.0000	5265.0	3.70%	5147.3	3.35%	motif file (matrix)	svg
126		MYB70(MYB)/col-MYB70-DAP-Seq(GSE60143)/Homer	1e-11	-2.745e+01	0.0000	33656.0	23.66%	35127.5	22.88%	motif file (matrix)	svg
127		Fra1(bZIP)/BT549-Fra1-ChIP-Seq(GSE46166)/Homer	1e-11	-2.710e+01	0.0000	7009.0	4.93%	6968.4	4.54%	motif file (matrix)	svg
128		At1g75490(AP2EREBP)/colamp-At1g75490-DAP-Seq(GSE60143)/Homer	1e-11	-2.709e+01	0.0000	15808.0	11.11%	16187.3	10.54%	motif file (matrix)	svg
129		MYB105(MYB)/colamp-MYB105-DAP-Seq(GSE60143)/Homer	1e-11	-2.695e+01	0.0000	14444.0	10.15%	14752.5	9.61%	motif file (matrix)	svg
130		dof42(C2C2dof)/col-dof42-DAP-Seq(GSE60143)/Homer	1e-11	-2.693e+01	0.0000	11901.0	8.37%	12079.4	7.87%	motif file (matrix)	svg
131		ERF7(AP2EREBP)/col-ERF7-DAP-Seq(GSE60143)/Homer	1e-11	-2.633e+01	0.0000	9480.0	6.66%	9553.0	6.22%	motif file (matrix)	svg
132		SPL11(SBP)/col100-SPL11-DAP-Seq(GSE60143)/Homer	1e-11	-2.616e+01	0.0000	15637.0	10.99%	16023.8	10.44%	motif file (matrix)	svg
133		Oct11(POU,Homeobox)/NCIH1048-POU2F3-ChIP-seq(GSE115123)/Homer	1e-11	-2.615e+01	0.0000	5819.0	4.09%	5746.4	3.74%	motif file (matrix)	svg
134		GTL1(Trihelix)/colamp-GTL1-DAP-Seq(GSE60143)/Homer	1e-11	-2.584e+01	0.0000	24029.0	16.89%	24915.9	16.23%	motif file (matrix)	svg
135		bZIP50(bZIP)/colamp-bZIP50-DAP-Seq(GSE60143)/Homer	1e-11	-2.566e+01	0.0000	13712.0	9.64%	14003.7	9.12%	motif file (matrix)	svg
136		ERF5(AP2EREBP)/colamp-ERF5-DAP-Seq(GSE60143)/Homer	1e-11	-2.552e+01	0.0000	2236.0	1.57%	2088.8	1.36%	motif file (matrix)	svg
137		AT5G02460(C2C2dof)/col-AT5G02460-DAP-Seq(GSE60143)/Homer	1e-11	-2.551e+01	0.0000	34131.0	24.00%	35681.3	23.24%	motif file (matrix)	svg
138		MYB101(MYB)/colamp-MYB101-DAP-Seq(GSE60143)/Homer	1e-10	-2.527e+01	0.0000	41245.0	29.00%	43292.5	28.20%	motif file (matrix)	svg
139		Fra2(bZIP)/Striatum-Fra2-ChIP-Seq(GSE43429)/Homer	1e-10	-2.438e+01	0.0000	5213.0	3.66%	5139.3	3.35%	motif file (matrix)	svg
140		GFX(?)/Promoter/Homer	1e-10	-2.381e+01	0.0000	309.0	0.22%	226.0	0.15%	motif file (matrix)	svg
141		SPL15(SBP)/colamp-SPL15-DAP-Seq(GSE60143)/Homer	1e-10	-2.360e+01	0.0000	29690.0	20.87%	30992.0	20.18%	motif file (matrix)	svg
142		SHN3(AP2EREBP)/col-SHN3-DAP-Seq(GSE60143)/Homer	1e-10	-2.324e+01	0.0000	1267.0	0.89%	1137.9	0.74%	motif file (matrix)	svg
143		CRF10(AP2EREBP)/col100-CRF10-DAP-Seq(GSE60143)/Homer	1e-9	-2.247e+01	0.0000	7539.0	5.30%	7578.7	4.94%	motif file (matrix)	svg
144		ANAC038(NAC)/col-ANAC038-DAP-Seq(GSE60143)/Homer	1e-9	-2.238e+01	0.0000	31481.0	22.13%	32932.4	21.45%	motif file (matrix)	svg
145		Zfp809(Zf)/ES-Zfp809-ChIP-Seq(GSE70799)/Homer	1e-9	-2.228e+01	0.0000	1731.0	1.22%	1603.4	1.04%	motif file (matrix)	svg
146		ZBTB33(Zf)/GM12878-ZBTB33-ChIP-Seq(GSE32465)/Homer	1e-9	-2.146e+01	0.0000	396.0	0.28%	309.1	0.20%	motif file (matrix)	svg
147		CRF4(AP2EREBP)/colamp-CRF4-DAP-Seq(GSE60143)/Homer	1e-9	-2.126e+01	0.0000	2529.0	1.78%	2415.4	1.57%	motif file (matrix)	svg
148		ERF8(AP2EREBP)/colamp-ERF8-DAP-Seq(GSE60143)/Homer	1e-9	-2.110e+01	0.0000	7982.0	5.61%	8060.1	5.25%	motif file (matrix)	svg
149		Atf3(bZIP)/GBM-ATF3-ChIP-Seq(GSE33912)/Homer	1e-8	-2.050e+01	0.0000	8666.0	6.09%	8785.5	5.72%	motif file (matrix)	svg
150		MITF(bHLH)/MastCells-MITF-ChIP-Seq(GSE48085)/Homer	1e-8	-2.027e+01	0.0000	13983.0	9.83%	14386.0	9.37%	motif file (matrix)	svg
151		RAP212(AP2EREBP)/col-RAP212-DAP-Seq(GSE60143)/Homer	1e-8	-1.993e+01	0.0000	5277.0	3.71%	5256.4	3.42%	motif file (matrix)	svg
152		RAP26(AP2EREBP)/colamp-RAP26-DAP-Seq(GSE60143)/Homer	1e-8	-1.930e+01	0.0000	8653.0	6.08%	8790.8	5.73%	motif file (matrix)	svg
153		SPCH(bHLH)/Seedling-SPCH-ChIP-Seq(GSE57497)/Homer	1e-8	-1.923e+01	0.0000	14740.0	10.36%	15207.8	9.90%	motif file (matrix)	svg
154		bHLH80(bHLH)/col-bHLH80-DAP-Seq(GSE60143)/Homer	1e-8	-1.900e+01	0.0000	13118.0	9.22%	13497.6	8.79%	motif file (matrix)	svg
155		RFX(HTH)/K562-RFX3-ChIP-Seq(SRA012198)/Homer	1e-8	-1.867e+01	0.0000	1065.0	0.75%	962.4	0.63%	motif file (matrix)	svg
156		ERF4(AP2EREBP)/colamp-ERF4-DAP-Seq(GSE60143)/Homer	1e-8	-1.865e+01	0.0000	7400.0	5.20%	7487.0	4.88%	motif file (matrix)	svg
157		E2F7(E2F)/Hela-E2F7-ChIP-Seq(GSE32673)/Homer	1e-8	-1.864e+01	0.0000	1009.0	0.71%	907.3	0.59%	motif file (matrix)	svg
158		HOXA9(Homeobox)/HSC-Hoxa9-ChIP-Seq(GSE33509)/Homer	1e-8	-1.857e+01	0.0000	12579.0	8.84%	12937.0	8.43%	motif file (matrix)	svg
159		At1g19000(MYBrelated)/colamp-At1g19000-DAP-Seq(GSE60143)/Homer	1e-7	-1.819e+01	0.0000	17817.0	12.53%	18490.6	12.04%	motif file (matrix)	svg
160		ANAC094(NAC)/col-ANAC094-DAP-Seq(GSE60143)/Homer	1e-7	-1.765e+01	0.0000	2470.0	1.74%	2385.6	1.55%	motif file (matrix)	svg
161		Cdx2(Homeobox)/mES-Cdx2-ChIP-Seq(GSE14586)/Homer	1e-7	-1.756e+01	0.0000	14809.0	10.41%	15315.8	9.97%	motif file (matrix)	svg
162		At5g65130(AP2EREBP)/colamp-At5g65130-DAP-Seq(GSE60143)/Homer	1e-7	-1.753e+01	0.0000	1390.0	0.98%	1292.6	0.84%	motif file (matrix)	svg
163		WT1(Zf)/Kidney-WT1-ChIP-Seq(GSE90016)/Homer	1e-7	-1.725e+01	0.0000	6454.0	4.54%	6518.7	4.25%	motif file (matrix)	svg
164		GAGA-repeat/Arabidopsis-Promoters/Homer	1e-7	-1.724e+01	0.0000	17570.0	12.35%	18250.5	11.89%	motif file (matrix)	svg
165		Olig2(bHLH)/Neuron-Olig2-ChIP-Seq(GSE30882)/Homer	1e-7	-1.715e+01	0.0000	29677.0	20.86%	31150.7	20.29%	motif file (matrix)	svg
166		Nkx2.2(Homeobox)/NPC-Nkx2.2-ChIP-Seq(GSE61673)/Homer	1e-7	-1.684e+01	0.0000	23654.0	16.63%	24732.3	16.11%	motif file (matrix)	svg
167		ERF9(AP2EREBP)/colamp-ERF9-DAP-Seq(GSE60143)/Homer	1e-7	-1.636e+01	0.0000	1576.0	1.11%	1487.9	0.97%	motif file (matrix)	svg
168		ERF115(AP2EREBP)/colamp-ERF115-DAP-Seq(GSE60143)/Homer	1e-7	-1.619e+01	0.0000	11337.0	7.97%	11671.8	7.60%	motif file (matrix)	svg
169		At5g18450(AP2EREBP)/col-At5g18450-DAP-Seq(GSE60143)/Homer	1e-6	-1.605e+01	0.0000	11958.0	8.41%	12330.3	8.03%	motif file (matrix)	svg
170		TGA5(bZIP)/col-TGA5-DAP-Seq(GSE60143)/Homer	1e-6	-1.590e+01	0.0000	2670.0	1.88%	2606.6	1.70%	motif file (matrix)	svg
171		DEL1(E2FDP)/colamp-DEL1-DAP-Seq(GSE60143)/Homer	1e-6	-1.585e+01	0.0000	39.0	0.03%	16.7	0.01%	motif file (matrix)	svg
172		Pax8(Paired,Homeobox)/Thyroid-Pax8-ChIP-Seq(GSE26938)/Homer	1e-6	-1.579e+01	0.0000	2594.0	1.82%	2529.8	1.65%	motif file (matrix)	svg
173		Hoxd12(Homeobox)/ChickenMSG-Hoxd12.Flag-ChIP-Seq(GSE86088)/Homer	1e-6	-1.574e+01	0.0000	33132.0	23.29%	34887.6	22.72%	motif file (matrix)	svg
174		SOC1(MADS)/Seedling-SOC1-ChIP-Seq(GSE45846)/Homer	1e-6	-1.571e+01	0.0000	6995.0	4.92%	7109.0	4.63%	motif file (matrix)	svg
175		ZNF341(Zf)/EBV-ZNF341-ChIP-Seq(GSE113194)/Homer	1e-6	-1.559e+01	0.0000	7442.0	5.23%	7579.3	4.94%	motif file (matrix)	svg
176		BMYB(HTH)/Hela-BMYB-ChIP-Seq(GSE27030)/Homer	1e-6	-1.547e+01	0.0000	28809.0	20.25%	30273.9	19.72%	motif file (matrix)	svg
177		DREB2(AP2EREBP)/col-DREB2-DAP-Seq(GSE60143)/Homer	1e-6	-1.513e+01	0.0000	3689.0	2.59%	3666.6	2.39%	motif file (matrix)	svg
178		Gli2(Zf)/GM2-Gli2-ChIP-Chip(GSE112702)/Homer	1e-6	-1.502e+01	0.0000	2391.0	1.68%	2328.2	1.52%	motif file (matrix)	svg
179		Zic2(Zf)/ESC-Zic2-ChIP-Seq(SRP197560)/Homer	1e-6	-1.501e+01	0.0000	4971.0	3.49%	5002.0	3.26%	motif file (matrix)	svg
180		TGA6(bZIP)/colamp-TGA6-DAP-Seq(GSE60143)/Homer	1e-6	-1.496e+01	0.0000	8850.0	6.22%	9071.6	5.91%	motif file (matrix)	svg
181		E2F(E2F)/Hela-CellCycle-Expression/Homer	1e-6	-1.467e+01	0.0000	871.0	0.61%	792.2	0.52%	motif file (matrix)	svg
182		ERF3(AP2EREBP)/colamp-ERF3-DAP-Seq(GSE60143)/Homer	1e-6	-1.467e+01	0.0000	3623.0	2.55%	3603.0	2.35%	motif file (matrix)	svg
183		TSO1(CPP)/col-TSO1-DAP-Seq(GSE60143)/Homer	1e-6	-1.467e+01	0.0000	2135.0	1.50%	2069.9	1.35%	motif file (matrix)	svg
184		ABR1(AP2EREBP)/colamp-ABR1-DAP-Seq(GSE60143)/Homer	1e-6	-1.443e+01	0.0000	7855.0	5.52%	8033.9	5.23%	motif file (matrix)	svg
185		Nkx2.5(Homeobox)/HL1-Nkx2.5.biotin-ChIP-Seq(GSE21529)/Homer	1e-6	-1.434e+01	0.0000	26972.0	18.96%	28345.5	18.46%	motif file (matrix)	svg
186		ERF11(AP2EREBP)/col-ERF11-DAP-Seq(GSE60143)/Homer	1e-6	-1.431e+01	0.0000	5288.0	3.72%	5342.9	3.48%	motif file (matrix)	svg
187		Gfi1b(Zf)/HPC7-Gfi1b-ChIP-Seq(GSE22178)/Homer	1e-6	-1.421e+01	0.0000	10994.0	7.73%	11349.0	7.39%	motif file (matrix)	svg
188		ANAC046(NAC)/colamp-ANAC046-DAP-Seq(GSE60143)/Homer	1e-6	-1.404e+01	0.0000	30047.0	21.12%	31642.3	20.61%	motif file (matrix)	svg
189		E2F1(E2F)/Hela-E2F1-ChIP-Seq(GSE22478)/Homer	1e-5	-1.371e+01	0.0000	1574.0	1.11%	1505.9	0.98%	motif file (matrix)	svg
190		Pknox1(Homeobox)/ES-Prep1-ChIP-Seq(GSE63282)/Homer	1e-5	-1.356e+01	0.0000	3183.0	2.24%	3160.0	2.06%	motif file (matrix)	svg
191		AT1G71450(AP2EREBP)/col-AT1G71450-DAP-Seq(GSE60143)/Homer	1e-5	-1.344e+01	0.0000	11601.0	8.16%	12008.1	7.82%	motif file (matrix)	svg
192		Srebp2(bHLH)/HepG2-Srebp2-ChIP-Seq(GSE31477)/Homer	1e-5	-1.289e+01	0.0000	1593.0	1.12%	1531.2	1.00%	motif file (matrix)	svg
193		NFkB-p65-Rel(RHD)/ThioMac-LPS-Expression(GSE23622)/Homer	1e-5	-1.276e+01	0.0000	915.0	0.64%	847.8	0.55%	motif file (matrix)	svg
194		Six4(Homeobox)/MCF7-SIX4-ChIP-Seq(Encode)/Homer	1e-5	-1.231e+01	0.0000	2630.0	1.85%	2602.1	1.69%	motif file (matrix)	svg
195		EBNA1(EBV-virus)/Raji-EBNA1-ChIP-Seq(GSE30709)/Homer	1e-5	-1.228e+01	0.0000	125.0	0.09%	88.4	0.06%	motif file (matrix)	svg
196		DEAR2(AP2EREBP)/colamp-DEAR2-DAP-Seq(GSE60143)/Homer	1e-5	-1.203e+01	0.0000	7234.0	5.09%	7424.0	4.84%	motif file (matrix)	svg
197		Rbpj1(?)/Panc1-Rbpj1-ChIP-Seq(GSE47459)/Homer	1e-5	-1.194e+01	0.0000	16177.0	11.37%	16904.1	11.01%	motif file (matrix)	svg
198		caudal(Homeobox)/Drosophila-Embryos-ChIP-Chip(modEncode)/Homer	1e-5	-1.153e+01	0.0000	15858.0	11.15%	16576.4	10.80%	motif file (matrix)	svg
199		At1g36060(AP2EREBP)/colamp-At1g36060-DAP-Seq(GSE60143)/Homer	1e-4	-1.124e+01	0.0001	4487.0	3.15%	4550.3	2.96%	motif file (matrix)	svg
200		Foxo3(Forkhead)/U2OS-Foxo3-ChIP-Seq(E-MTAB-2701)/Homer	1e-4	-1.104e+01	0.0001	13907.0	9.78%	14514.8	9.45%	motif file (matrix)	svg
201		Hoxa11(Homeobox)/ChickenMSG-Hoxa11.Flag-ChIP-Seq(GSE86088)/Homer	1e-4	-1.076e+01	0.0001	45789.0	32.19%	48651.4	31.69%	motif file (matrix)	svg
202		AT5G60130(ABI3VP1)/col-AT5G60130-DAP-Seq(GSE60143)/Homer	1e-4	-1.059e+01	0.0001	28680.0	20.16%	30300.6	19.73%	motif file (matrix)	svg
203		Rfx2(HTH)/LoVo-RFX2-ChIP-Seq(GSE49402)/Homer	1e-4	-1.058e+01	0.0001	1205.0	0.85%	1155.6	0.75%	motif file (matrix)	svg
204		SPL14(SBP)/col-SPL14-DAP-Seq(GSE60143)/Homer	1e-4	-1.058e+01	0.0001	2679.0	1.88%	2673.6	1.74%	motif file (matrix)	svg
205		BIM2(bHLH)/col-BIM2-DAP-Seq(GSE60143)/Homer	1e-4	-1.056e+01	0.0001	14996.0	10.54%	15686.3	10.22%	motif file (matrix)	svg
206		SeqBias: GA-repeat	1e-4	-1.055e+01	0.0001	80127.0	56.33%	85678.0	55.80%	motif file (matrix)	svg
207		AP-1(bZIP)/ThioMac-PU.1-ChIP-Seq(GSE21512)/Homer	1e-4	-1.053e+01	0.0001	9110.0	6.40%	9436.1	6.15%	motif file (matrix)	svg
208		AT4G00250(GeBP)/col-AT4G00250-DAP-Seq(GSE60143)/Homer	1e-4	-1.037e+01	0.0002	12059.0	8.48%	12568.5	8.19%	motif file (matrix)	svg
209		AT5G22990(C2H2)/col-AT5G22990-DAP-Seq(GSE60143)/Homer	1e-4	-1.019e+01	0.0002	1194.0	0.84%	1147.6	0.75%	motif file (matrix)	svg
210		DREB26(AP2EREBP)/col-DREB26-DAP-Seq(GSE60143)/Homer	1e-4	-9.941e+00	0.0002	1192.0	0.84%	1147.2	0.75%	motif file (matrix)	svg
211		PLT1(AP2EREBP)/colamp-PLT1-DAP-Seq(GSE60143)/Homer	1e-4	-9.726e+00	0.0003	612.0	0.43%	563.1	0.37%	motif file (matrix)	svg
212		Pbx3(Homeobox)/GM12878-PBX3-ChIP-Seq(GSE32465)/Homer	1e-4	-9.659e+00	0.0003	3252.0	2.29%	3282.8	2.14%	motif file (matrix)	svg
213		PIF7(bHLH)/col-PIF7-DAP-Seq(GSE60143)/Homer	1e-4	-9.368e+00	0.0004	1369.0	0.96%	1333.4	0.87%	motif file (matrix)	svg
214		ERF10(AP2EREBP)/col-ERF10-DAP-Seq(GSE60143)/Homer	1e-4	-9.265e+00	0.0004	3433.0	2.41%	3477.2	2.26%	motif file (matrix)	svg
215		LBD23(LOBAS2)/colamp-LBD23-DAP-Seq(GSE60143)/Homer	1e-3	-9.151e+00	0.0005	10161.0	7.14%	10584.2	6.89%	motif file (matrix)	svg
216		DEAR3(AP2EREBP)/colamp-DEAR3-DAP-Seq(GSE60143)/Homer	1e-3	-9.089e+00	0.0005	2507.0	1.76%	2513.4	1.64%	motif file (matrix)	svg
217		HEB(bHLH)/mES-Heb-ChIP-Seq(GSE53233)/Homer	1e-3	-8.958e+00	0.0006	23116.0	16.25%	24407.7	15.90%	motif file (matrix)	svg
218		TGA1(bZIP)/colamp-TGA1-DAP-Seq(GSE60143)/Homer	1e-3	-8.796e+00	0.0007	6565.0	4.62%	6782.4	4.42%	motif file (matrix)	svg
219		Fosl2(bZIP)/3T3L1-Fosl2-ChIP-Seq(GSE56872)/Homer	1e-3	-8.782e+00	0.0007	2649.0	1.86%	2665.2	1.74%	motif file (matrix)	svg
220		Nr5a2(NR)/Pancreas-LRH1-ChIP-Seq(GSE34295)/Homer	1e-3	-8.600e+00	0.0008	6245.0	4.39%	6448.7	4.20%	motif file (matrix)	svg
221		AT2G15740(C2H2)/col-AT2G15740-DAP-Seq(GSE60143)/Homer	1e-3	-8.574e+00	0.0009	11078.0	7.79%	11574.8	7.54%	motif file (matrix)	svg
222		AT5G47660(Trihelix)/colamp-AT5G47660-DAP-Seq(GSE60143)/Homer	1e-3	-8.519e+00	0.0009	28983.0	20.38%	30708.2	20.00%	motif file (matrix)	svg
223		FOXP1(Forkhead)/H9-FOXP1-ChIP-Seq(GSE31006)/Homer	1e-3	-8.284e+00	0.0011	8198.0	5.76%	8523.2	5.55%	motif file (matrix)	svg
224		PUCHI(AP2EREBP)/colamp-PUCHI-DAP-Seq(GSE60143)/Homer	1e-3	-8.258e+00	0.0012	3961.0	2.78%	4047.5	2.64%	motif file (matrix)	svg
225		AT1G28160(AP2EREBP)/colamp-AT1G28160-DAP-Seq(GSE60143)/Homer	1e-3	-8.117e+00	0.0013	11452.0	8.05%	11985.7	7.81%	motif file (matrix)	svg
226		JunD(bZIP)/K562-JunD-ChIP-Seq/Homer	1e-3	-8.112e+00	0.0013	2906.0	2.04%	2943.0	1.92%	motif file (matrix)	svg
227		TGA3(bZIP)/colamp-TGA3-DAP-Seq(GSE60143)/Homer	1e-3	-7.883e+00	0.0017	2049.0	1.44%	2052.2	1.34%	motif file (matrix)	svg
228		MYB33(MYB)/col-MYB33-DAP-Seq(GSE60143)/Homer	1e-3	-7.874e+00	0.0017	36653.0	25.77%	38968.7	25.38%	motif file (matrix)	svg
229		Unknown-ESC-element(?)/mES-Nanog-ChIP-Seq(GSE11724)/Homer	1e-3	-7.801e+00	0.0018	6800.0	4.78%	7053.7	4.59%	motif file (matrix)	svg
230		bHLHE41(bHLH)/proB-Bhlhe41-ChIP-Seq(GSE93764)/Homer	1e-3	-7.665e+00	0.0021	14939.0	10.50%	15716.9	10.24%	motif file (matrix)	svg
231		GT2(Trihelix)/colamp-GT2-DAP-Seq(GSE60143)/Homer	1e-3	-7.434e+00	0.0026	22804.0	16.03%	24134.5	15.72%	motif file (matrix)	svg
232		TGA4(bZIP)/colamp-TGA4-DAP-Seq(GSE60143)/Homer	1e-3	-7.386e+00	0.0027	4779.0	3.36%	4925.9	3.21%	motif file (matrix)	svg
233		Hand2(bHLH)/Mesoderm-Hand2-ChIP-Seq(GSE61475)/Homer	1e-3	-7.248e+00	0.0031	5102.0	3.59%	5269.0	3.43%	motif file (matrix)	svg
234		COG1(C2C2dof)/col-COG1-DAP-Seq(GSE60143)/Homer	1e-3	-7.211e+00	0.0032	24663.0	17.34%	26135.4	17.02%	motif file (matrix)	svg
235		ANAC042(NAC)/col-ANAC042-DAP-Seq(GSE60143)/Homer	1e-2	-6.898e+00	0.0043	3815.0	2.68%	3918.4	2.55%	motif file (matrix)	svg
236		ETS:RUNX(ETS,Runt)/Jurkat-RUNX1-ChIP-Seq(GSE17954)/Homer	1e-2	-6.858e+00	0.0045	754.0	0.53%	726.6	0.47%	motif file (matrix)	svg
237		CDF3(C2C2dof)/colamp-CDF3-DAP-Seq(GSE60143)/Homer	1e-2	-6.757e+00	0.0049	28880.0	20.30%	30678.7	19.98%	motif file (matrix)	svg
238		RRTF1(AP2EREBP)/colamp-RRTF1-DAP-Seq(GSE60143)/Homer	1e-2	-6.632e+00	0.0056	2425.0	1.70%	2462.1	1.60%	motif file (matrix)	svg
239		AT1G44830(AP2EREBP)/col-AT1G44830-DAP-Seq(GSE60143)/Homer	1e-2	-6.584e+00	0.0058	1614.0	1.13%	1616.0	1.05%	motif file (matrix)	svg
240		At1g74840(MYBrelated)/col100-At1g74840-DAP-Seq(GSE60143)/Homer	1e-2	-6.547e+00	0.0060	16027.0	11.27%	16920.0	11.02%	motif file (matrix)	svg
241		SCL(bHLH)/HPC7-Scl-ChIP-Seq(GSE13511)/Homer	1e-2	-6.466e+00	0.0065	62379.0	43.86%	66739.0	43.47%	motif file (matrix)	svg
242		REST-NRSF(Zf)/Jurkat-NRSF-ChIP-Seq/Homer	1e-2	-6.423e+00	0.0067	99.0	0.07%	78.3	0.05%	motif file (matrix)	svg
243		bZIP28(bZIP)/col-bZIP28-DAP-Seq(GSE60143)/Homer	1e-2	-6.323e+00	0.0074	2240.0	1.57%	2273.6	1.48%	motif file (matrix)	svg
244		AT3G16280(AP2EREBP)/colamp-AT3G16280-DAP-Seq(GSE60143)/Homer	1e-2	-6.280e+00	0.0077	2876.0	2.02%	2941.0	1.92%	motif file (matrix)	svg
245		IDD4(C2H2)/col-IDD4-DAP-	[illegible]	[illegible]	[illegible]	[illegible]	[illegible]	[illegible]	[illegible]	motif	[illegible]

245	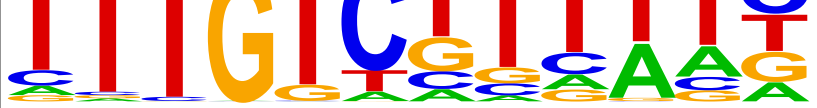	IDD4(C2H2)/col-IDD4-DAP-Seq(GSE60143)/Homer	1e-2	-6.111e+00	0.0091	12730.0	8.95%	13413.8	8.74%	motif file (matrix)	svg
246	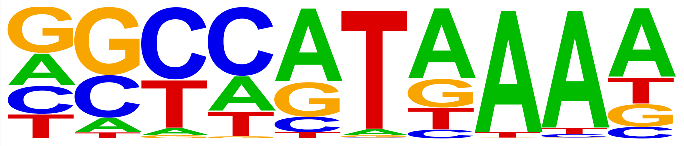	Hoxd11(Homeobox)/ChickenMSG-Hoxd11.Flag-ChIP-Seq(GSE86088)/Homer	1e-2	-6.041e+00	0.0097	43261.0	30.41%	46172.0	30.07%	motif file (matrix)	svg
247	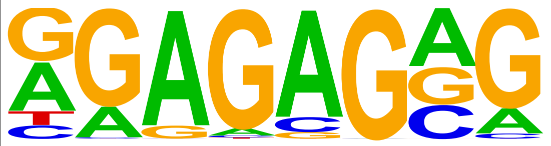	Trl(Zf)/S2-GAGAfactor-ChIP-Seq(GSE40646)/Homer	1e-2	-5.888e+00	0.0113	31748.0	22.32%	33802.9	22.02%	motif file (matrix)	svg
248	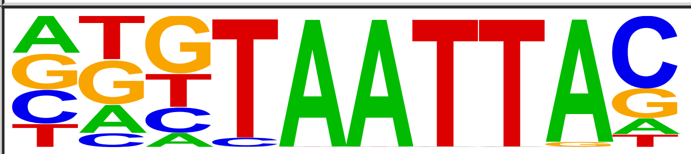	Dlx3(Homeobox)/Kerainocytes-Dlx3-ChIP-Seq(GSE89884)/Homer	1e-2	-5.867e+00	0.0115	14234.0	10.01%	15029.4	9.79%	motif file (matrix)	svg
249	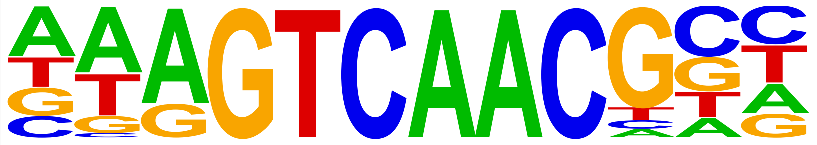	WRKY46(WRKY)/colamp-WRKY46-DAP-Seq(GSE60143)/Homer	1e-2	-5.816e+00	0.0120	424.0	0.30%	399.1	0.26%	motif file (matrix)	svg
250	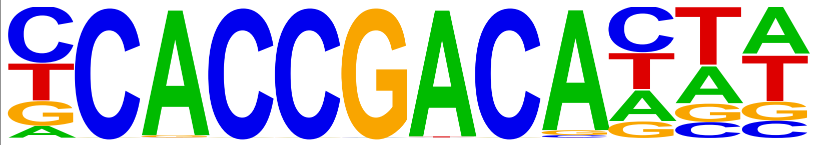	AT1G01250(AP2EREBP)/col-AT1G01250-DAP-Seq(GSE60143)/Homer	1e-2	-5.643e+00	0.0143	607.0	0.43%	586.2	0.38%	motif file (matrix)	svg
251	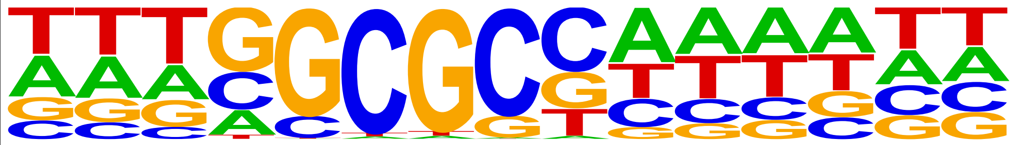	E2FA(E2FDP)/colamp-E2FA-DAP-Seq(GSE60143)/Homer	1e-2	-5.443e+00	0.0173	6938.0	4.88%	7261.6	4.73%	motif file (matrix)	svg
252	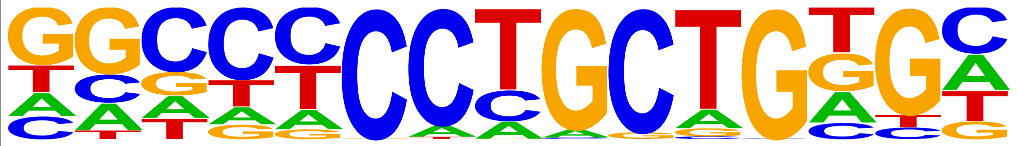	Zic3(Zf)/mES-Zic3-ChIP-Seq(GSE37889)/Homer	1e-2	-5.344e+00	0.0191	5653.0	3.97%	5898.8	3.84%	motif file (matrix)	svg
253	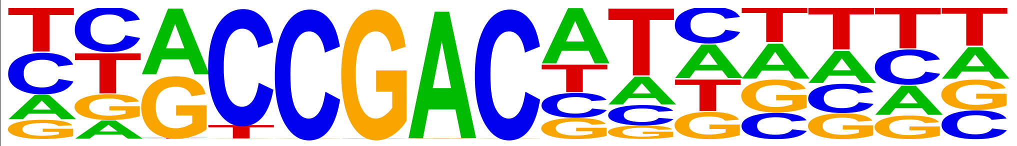	CBF2(AP2EREBP)/colamp-CBF2-DAP-Seq(GSE60143)/Homer	1e-2	-5.065e+00	0.0251	3953.0	2.78%	4102.2	2.67%	motif file (matrix)	svg
254	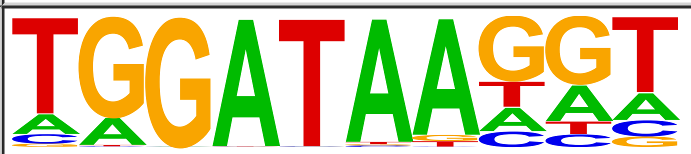	AT5G56840(MYBrelated)/colamp-AT5G56840-DAP-Seq(GSE60143)/Homer	1e-2	-4.975e+00	0.0274	23494.0	16.52%	24990.3	16.28%	motif file (matrix)	svg
255	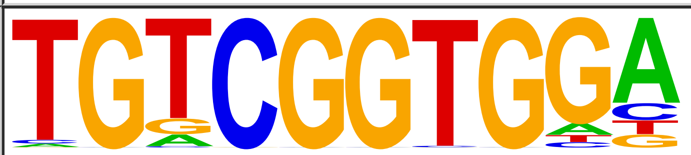	At1g77640(AP2EREBP)/col-At1g77640-DAP-Seq(GSE60143)/Homer	1e-2	-4.937e+00	0.0283	1001.0	0.70%	999.5	0.65%	motif file (matrix)	svg
256	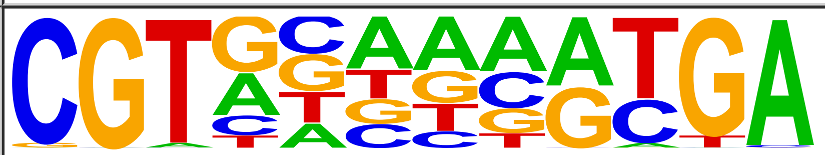	ABF1/SacCer-Promoters/Homer	1e-2	-4.918e+00	0.0287	2202.0	1.55%	2257.0	1.47%	motif file (matrix)	svg
257	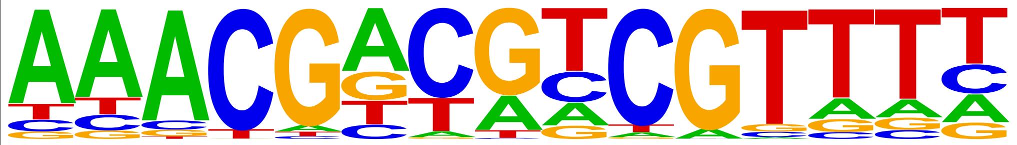	Unknown2/Arabidopsis-Promoters/Homer	1e-2	-4.917e+00	0.0287	256.0	0.18%	236.5	0.15%	motif file (matrix)	svg
258	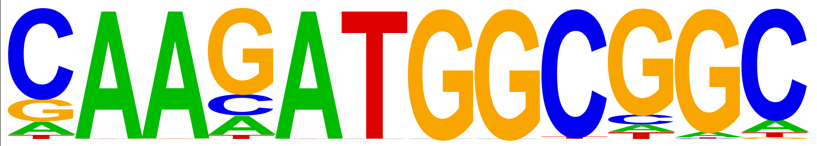	YY1(Zf)/Promoter/Homer	1e-2	-4.842e+00	0.0308	1532.0	1.08%	1554.6	1.01%	motif file (matrix)	svg
259	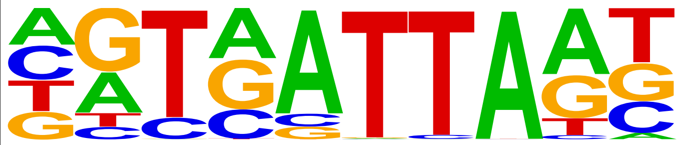	ATHB33(ZFHD)/col-ATHB33-DAP-Seq(GSE60143)/Homer	1e-2	-4.701e+00	0.0353	23658.0	16.63%	25181.8	16.40%	motif file (matrix)	svg
260	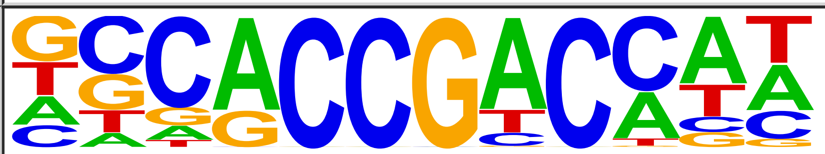	At4g28140(AP2EREBP)/colamp-At4g28140-DAP-Seq(GSE60143)/Homer	1e-2	-4.655e+00	0.0368	1881.0	1.32%	1923.0	1.25%	motif file (matrix)	svg
261	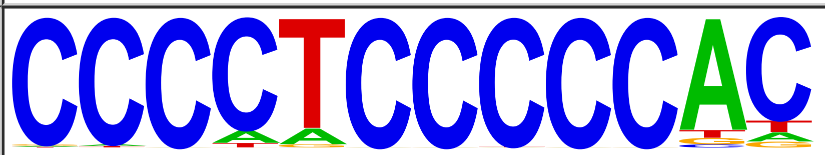	Zfp281(Zf)/ES-Zfp281-ChIP-Seq(GSE81042)/Homer	1e-2	-4.635e+00	0.0374	1488.0	1.05%	1511.8	0.98%	motif file (matrix)	svg
262	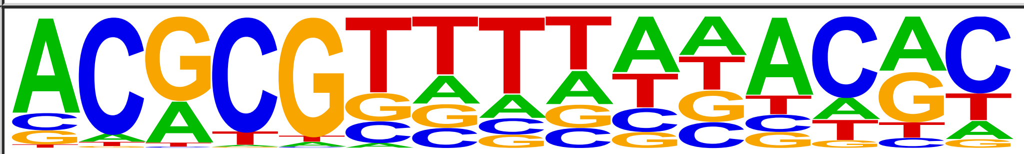	CAMTA5(CAMTA)/col-CAMTA5-DAP-Seq(GSE60143)/Homer	1e-2	-4.623e+00	0.0377	3235.0	2.27%	3352.6	2.18%	motif file (matrix)	svg

Homer Known Motif Enrichment Results (allnoMT_condition2_txt_motif)

Gained Peaks after 3pmol Foxi1MO

Homer *de novo* Motif Results
Gene Ontology Enrichment Results
Known Motif Enrichment Results (txt file)
Total Target Sequences = 3489, Total Background Sequences = 302154

Rank	Motif	Name	P-value	log P-pvalue	q-value (Benjamini)	# Target Sequences with Motif	% of Targets Sequences with Motif	# Background Sequences with Motif	% of Background Sequences with Motif	Motif File	SVG
1		Brn1(POU,Homeobox)/NPC-Brn1-ChIP-Seq(GSE35496)/Homer	1e-16	-3.757e+01	0.0000	306.0	8.77%	16074.6	5.32%	motif file (matrix)	svg
2		EIN3(EIL)/col-EIN3-DAP-Seq(GSE60143)/Homer	1e-14	-3.290e+01	0.0000	168.0	4.82%	7563.5	2.51%	motif file (matrix)	svg
3		Nur77(NR)/K562-NR4A1-ChIP-Seq(GSE31363)/Homer	1e-14	-3.285e+01	0.0000	138.0	3.96%	5745.6	1.90%	motif file (matrix)	svg
4		Oct6(POU,Homeobox)/NPC-Pou3f1-ChIP-Seq(GSE35496)/Homer	1e-11	-2.746e+01	0.0000	362.0	10.38%	21498.0	7.12%	motif file (matrix)	svg
5		Oct4(POU,Homeobox)/mES-Oct4-ChIP-Seq(GSE11431)/Homer	1e-9	-2.143e+01	0.0000	405.0	11.61%	25844.6	8.56%	motif file (matrix)	svg
6		Hoxa10(Homeobox)/ChickenMSG-Hoxa10.Flag-ChIP-Seq(GSE86088)/Homer	1e-9	-2.104e+01	0.0000	338.0	9.69%	20926.8	6.93%	motif file (matrix)	svg
7		HDG7(HB)/col-HDG7-DAP-Seq(GSE60143)/Homer	1e-8	-2.019e+01	0.0000	205.0	5.88%	11485.4	3.80%	motif file (matrix)	svg
8		Fosl2(bZIP)/3T3L1-Fosl2-ChIP-Seq(GSE56872)/Homer	1e-8	-1.874e+01	0.0000	118.0	3.38%	5788.9	1.92%	motif file (matrix)	svg
9		ZNF652/HepG2-ZNF652.Flag-ChIP-Seq(Encode)/Homer	1e-7	-1.709e+01	0.0000	214.0	6.13%	12620.1	4.18%	motif file (matrix)	svg
10		ATHB34(ZFHD)/colamp-ATHB34-DAP-Seq(GSE60143)/Homer	1e-7	-1.698e+01	0.0000	579.0	16.60%	40446.4	13.40%	motif file (matrix)	svg
11		OCT4-SOX2-TCF-NANOG(POU,Homeobox,HMG)/mES-Oct4-ChIP-Seq(GSE11431)/Homer	1e-7	-1.641e+01	0.0000	225.0	6.45%	13535.4	4.48%	motif file (matrix)	svg
12		AT1G76880(Trihelix)/col-AT1G76880-DAP-Seq(GSE60143)/Homer	1e-6	-1.535e+01	0.0000	342.0	9.80%	22477.0	7.45%	motif file (matrix)	svg
13		ATHB23(ZFHD)/col-ATHB23-DAP-Seq(GSE60143)/Homer	1e-6	-1.467e+01	0.0000	649.0	18.60%	46781.7	15.50%	motif file (matrix)	svg
14		dof42(C2C2dof)/col-dof42-DAP-Seq(GSE60143)/Homer	1e-6	-1.413e+01	0.0001	414.0	11.87%	28346.1	9.39%	motif file (matrix)	svg
15		Jun-AP1(bZIP)/K562-cJun-ChIP-Seq(GSE31477)/Homer	1e-5	-1.363e+01	0.0001	78.0	2.24%	3754.9	1.24%	motif file (matrix)	svg
16		HDG1(Homeobox)/col100-HDG1-DAP-Seq(GSE60143)/Homer	1e-5	-1.318e+01	0.0001	484.0	13.87%	34131.5	11.31%	motif file (matrix)	svg
17		Mef2b(MADS)/HEK293-Mef2b.V5-ChIP-Seq(GSE67450)/Homer	1e-5	-1.316e+01	0.0001	475.0	13.61%	33428.4	11.07%	motif file (matrix)	svg
18		ASHR1(ND)/col-ASHR1-DAP-Seq(GSE60143)/Homer	1e-5	-1.212e+01	0.0003	405.0	11.61%	28243.5	9.36%	motif file (matrix)	svg
19		Prop1(Homeobox)/GHFT1-PROP1.biotin-ChIP-Seq(GSE77302)/Homer	1e-4	-1.128e+01	0.0007	478.0	13.70%	34302.7	11.36%	motif file (matrix)	svg
20		ATHB24(ZFHD)/colamp-ATHB24-DAP-Seq(GSE60143)/Homer	1e-4	-1.117e+01	0.0007	542.0	15.53%	39466.1	13.07%	motif file (matrix)	svg
21		COUP-TFII(NR)/K562-NR2F1-ChIP-Seq(Encode)/Homer	1e-4	-1.108e+01	0.0007	415.0	11.89%	29370.6	9.73%	motif file (matrix)	svg
22		TBP3(MYBrelated)/col-TBP3-DAP-Seq(GSE60143)/Homer	1e-4	-1.090e+01	0.0008	323.0	9.26%	22208.8	7.36%	motif file (matrix)	svg
23		ATHB25(ZFHD)/colamp-ATHB25-DAP-Seq(GSE60143)/Homer	1e-4	-1.079e+01	0.0009	754.0	21.61%	56865.7	18.84%	motif file (matrix)	svg
24		GTL1(Trihelix)/colamp-GTL1-DAP-Seq(GSE60143)/Homer	1e-4	-1.015e+01	0.0016	799.0	22.90%	60870.8	20.16%	motif file (matrix)	svg
25		JunB(bZIP)/DendriticCells-Junb-ChIP-Seq(GSE36099)/Homer	1e-4	-1.013e+01	0.0016	180.0	5.16%	11486.3	3.80%	motif file (matrix)	svg
26		Sox10(HMG)/SciaticNerve-Sox3-ChIP-Seq(GSE35132)/Homer	1e-4	-1.006e+01	0.0017	763.0	21.87%	57944.0	19.19%	motif file (matrix)	svg
27		Lhx1(Homeobox)/EmbryoCarcinoma-Lhx1-ChIP-Seq(GSE70957)/Homer	1e-4	-9.224e+00	0.0037	800.0	22.93%	61411.3	20.34%	motif file (matrix)	svg
28		ZEB2(Zf)/SNU398-ZEB2-ChIP-Seq(GSE103048)/Homer	1e-3	-9.030e+00	0.0043	280.0	8.03%	19425.6	6.43%	motif file (matrix)	svg
29		E2A(bHLH),near_PU.1/Bcell-PU.1-ChIP-Seq(GSE21512)/Homer	1e-3	-8.978e+00	0.0044	381.0	10.92%	27406.8	9.08%	motif file (matrix)	svg
30		Lhx3(Homeobox)/Neuron-Lhx3-ChIP-Seq(GSE31456)/Homer	1e-3	-8.895e+00	0.0046	1091.0	31.27%	85891.0	28.45%	motif file (matrix)	svg
31		Sox7(HMG)/ESC-Sox7-ChIP-Seq(GSE133899)/Homer	1e-3	-8.834e+00	0.0047	251.0	7.19%	17229.9	5.71%	motif file (matrix)	svg
32		At1g76110(ARID)/colamp-At1g76110-DAP-Seq(GSE60143)/Homer	1e-3	-8.728e+00	0.0051	507.0	14.53%	37629.3	12.46%	motif file (matrix)	svg
33		MYB55(MYB)/colamp-MYB55-DAP-Seq(GSE60143)/Homer	1e-3	-8.554e+00	0.0059	430.0	12.32%	31488.9	10.43%	motif file (matrix)	svg
34		SeqBias: A/T bias	1e-3	-8.552e+00	0.0059	3427.0	98.22%	293693.8	97.29%	motif file (matrix)	svg
35		Oct2(POU,Homeobox)/Bcell-Oct2-ChIP-Seq(GSE21512)/Homer	1e-3	-8.401e+00	0.0065	223.0	6.39%	15189.6	5.03%	motif file (matrix)	svg
36		Sox2(HMG)/mES-Sox2-ChIP-Seq(GSE11431)/Homer	1e-3	-8.311e+00	0.0069	485.0	13.90%	36027.2	11.93%	motif file (matrix)	svg
37		CCA(Myb)/Arabidopsis-CCA.GFP-ChIP-Seq(GSE70533)/Homer	1e-3	-8.257e+00	0.0071	425.0	12.18%	31206.2	10.34%	motif file (matrix)	svg
38		DLX2(Homeobox)/BasalGanglia-Dlx2-ChIP-seq(GSE124936)/Homer	1e-3	-8.032e+00	0.0086	1011.0	28.98%	79681.5	26.39%	motif file (matrix)	svg
39		Npas4(bHLH)/Neuron-Npas4-ChIP-Seq(GSE127793)/Homer	1e-3	-8.031e+00	0.0086	217.0	6.22%	14832.4	4.91%	motif file (matrix)	svg
40		Oct11(POU,Homeobox)/NCIH1048-POU2F3-ChIP-seq(GSE115123)/Homer	1e-3	-7.939e+00	0.0090	242.0	6.94%	16800.7	5.57%	motif file (matrix)	svg
41		CAMTA5(CAMTA)/col-CAMTA5-DAP-Seq(GSE60143)/Homer	1e-3	-7.915e+00	0.0090	108.0	3.10%	6633.4	2.20%	motif file (matrix)	svg
42		NGA4(ABI3VP1)/col-NGA4-DAP-Seq(GSE60143)/Homer	1e-3	-7.872e+00	0.0091	797.0	22.84%	61886.3	20.50%	motif file (matrix)	svg
43		NAP(NAC)/col-NAP-DAP-Seq(GSE60143)/Homer	1e-3	-7.835e+00	0.0093	316.0	9.06%	22657.9	7.51%	motif file (matrix)	svg
44		OCT:OCT-short(POU,Homeobox)/NPC-OCT6-ChIP-Seq(GSE43916)/Homer	1e-3	-7.827e+00	0.0093	458.0	13.13%	34051.4	11.28%	motif file (matrix)	svg
45		DLX1(Homeobox)/BasalGanglia-Dlx1-ChIP-seq(GSE124936)/Homer	1e-3	-7.822e+00	0.0093	906.0	25.97%	71005.2	23.52%	motif file (matrix)	svg
46		LXRE(NR),DR4/RAW-LXRb.biotin-ChIP-Seq(GSE21512)/Homer	1e-3	-7.725e+00	0.0097	24.0	0.69%	950.1	0.31%	motif file (matrix)	svg
47		At1g64620(C2C2dof)/colamp-At1g64620-DAP-Seq(GSE60143)/Homer	1e-3	-7.680e+00	0.0099	857.0	24.56%	66995.2	22.19%	motif file (matrix)	svg
48		GT2(Trihelix)/colamp-GT2-DAP-Seq(GSE60143)/Homer	1e-3	-7.664e+00	0.0099	794.0	22.76%	61754.2	20.46%	motif file (matrix)	svg
49		At5g62940(C2C2dof)/col-At5g62940-DAP-Seq(GSE60143)/Homer	1e-3	-7.624e+00	0.0100	1759.0	50.42%	143732.9	47.61%	motif file (matrix)	svg
50		HuR(?)/HEK293-HuR-CLIP-Seq(GSE87887)/Homer	1e-3	-7.300e+00	0.0136	1864.0	53.43%	153052.7	50.70%	motif file (matrix)	svg
51		MYB93(MYB)/colamp-MYB93-DAP-Seq(GSE60143)/Homer	1e-3	-7.129e+00	0.0158	403.0	11.55%	29907.1	9.91%	motif file (matrix)	svg
52		ANL2(HB)/col-ANL2-DAP-Seq(GSE60143)/Homer	1e-3	-7.127e+00	0.0158	431.0	12.35%	32174.8	10.66%	motif file (matrix)	svg
53		At5g47390(MYBrelated)/col-At5g47390-DAP-Seq(GSE60143)/Homer	1e-3	-7.116e+00	0.0158	670.0	19.20%	51774.7	17.15%	motif file (matrix)	svg
54		Bach2(bZIP)/OCILy7-Bach2-ChIP-Seq(GSE44420)/Homer	1e-3	-7.009e+00	0.0168	75.0	2.15%	4415.1	1.46%	motif file (matrix)	svg
55		MYB107(MYB)/col-MYB107-DAP-Seq(GSE60143)/Homer	1e-2	-6.893e+00	0.0186	554.0	15.88%	42329.1	14.02%	motif file (matrix)	svg
56		NPAS2(bHLH)/Liver-NPAS2-ChIP-Seq(GSE39860)/Homer	1e-2	-6.830e+00	0.0194	294.0	8.43%	21281.5	7.05%	motif file (matrix)	svg
57		Pit1(Homeobox)/GCrat-Pit1-ChIP-Seq(GSE58009)/Homer	1e-2	-6.790e+00	0.0199	632.0	18.11%	48812.5	16.17%	motif file (matrix)	svg
58		Znf263(Zf)/K562-Znf263-ChIP-Seq(GSE31477)/Homer	1e-2	-6.762e+00	0.0201	421.0	12.07%	31529.1	10.44%	motif file (matrix)	svg
59		LHX9(Homeobox)/Hct116-LHX9.V5-ChIP-Seq(GSE116822)/Homer	1e-2	-6.683e+00	0.0213	866.0	24.82%	68358.7	22.64%	motif file (matrix)	svg
60		Snail1(Zf)/LS174T-SNAIL1.HA-ChIP-Seq(GSE127183)/Homer	1e-2	-6.677e+00	0.0213	251.0	7.19%	17925.7	5.94%	motif file (matrix)	svg
61		GATA3(Zf)/iTreg-Gata3-ChIP-Seq(GSE20898)/Homer	1e-2	-6.454e+00	0.0260	788.0	22.59%	61978.5	20.53%	motif file (matrix)	svg
62		DAG2(C2C2dof)/col-DAG2-DAP-Seq(GSE60143)/Homer	1e-2	-6.433e+00	0.0261	1029.0	29.49%	82237.5	27.24%	motif file (matrix)	svg
63		NFE2L2(bZIP)/HepG2-NFE2L2-ChIP-Seq(Encode)/Homer	1e-2	-6.401e+00	0.0265	20.0	0.57%	812.0	0.27%	motif file (matrix)	svg
64		NFIL3(bZIP)/HepG2-NFIL3-ChIP-Seq(Encode)/Homer	1e-2	-6.295e+00	0.0290	430.0	12.32%	32482.5	10.76%	motif file (matrix)	svg
65		Tgif1(Homeobox)/mES-Tgif1-ChIP-Seq(GSE55404)/Homer	1e-2	-6.233e+00	0.0304	1193.0	34.19%	96274.2	31.89%	motif file (matrix)	svg
66		Sox6(HMG)/Myotubes-Sox6-ChIP-Seq(GSE32627)/Homer	1e-2	-6.227e+00	0.0304	829.0	23.76%	65554.7	21.72%	motif file (matrix)	svg
67		Foxf1(Forkhead)/Lung-Foxf1-ChIP-Seq(GSE77951)/Homer	1e-2	-6.150e+00	0.0320	646.0	18.52%	50340.4	16.68%	motif file (matrix)	svg
68		Isl1(Homeobox)/Neuron-Isl1-ChIP-Seq(GSE31456)/Homer	1e-2	-6.134e+00	0.0321	1041.0	29.84%	83463.6	27.65%	motif file (matrix)	svg
69		Unknown2/Drosophila-Promoters/Homer	1e-2	-6.108e+00	0.0324	452.0	12.96%	34371.7	11.39%	motif file (matrix)	svg
70		At3g24120(G2like)/col-At3g24120-DAP-Seq(GSE60143)/Homer	1e-2	-5.982e+00	0.0363	1372.0	39.32%	111724.6	37.01%	motif file (matrix)	svg
71		At1g69690(TCP)/colamp-At1g69690-DAP-Seq(GSE60143)/Homer	1e-2	-5.981e+00	0.0363	83.0	2.38%	5185.5	1.72%	motif file (matrix)	svg
72		AT3G51470(DBP)/col-AT3G51470-DAP-Seq(GSE60143)/Homer	1e-2	-5.966e+00	0.0363	641.0	18.37%	50035.3	16.57%	motif file (matrix)	svg
73		PR(NR)/T47D-PR-ChIP-Seq(GSE31130)/Homer	1e-2	-5.939e+00	0.0363	811.0	23.24%	64237.5	21.28%	motif file (matrix)	svg
74		MYB58(MYB)/colamp-MYB58-DAP-Seq(GSE60143)/Homer	1e-2	-5.913e+00	0.0367	347.0	9.95%	25920.0	8.59%	motif file (matrix)	svg
75		AT3G52440(C2C2dof)/colamp-AT3G52440-DAP-Seq(GSE60143)/Homer	1e-2	-5.872e+00	0.0378	1335.0	38.26%	108650.6	35.99%	motif file (matrix)	svg
76		HOXA1(Homeobox)/mES-Hoxa1-ChIP-Seq(SRP084292)/Homer	1e-2	-5.871e+00	0.0378	118.0	3.38%	7830.4	2.59%	motif file (matrix)	svg
77		Hoxa13(Homeobox)/ChickenMSG-Hoxa13.Flag-ChIP-Seq(GSE86088)/Homer	1e-2	-5.861e+00	0.0378	1394.0	39.95%	113702.4	37.66%	motif file (matrix)	svg
78		Lhx2(Homeobox)/HFSC-Lhx2-ChIP-Seq(GSE48068)/Homer	1e-2	-5.667e+00	0.0446	702.0	20.12%	55301.8	18.32%	motif file (matrix)	svg
79		MYB61(MYB)/colamp-MYB61-DAP-Seq(GSE60143)/Homer	1e-2	-5.628e+00	0.0458	522.0	14.96%	40382.3	13.38%	motif file (matrix)	svg
80		AT3G25990(Trihelix)/colamp-AT3G25990-DAP-Seq(GSE60143)/Homer	1e-2	-5.595e+00	0.0467	381.0	10.92%	28831.2	9.55%	motif file (matrix)	svg
81		SRS7(SRS)/colamp-SRS7-DAP-Seq(GSE60143)/Homer	1e-2	-5.593e+00	0.0467	1252.0	35.88%	101791.9	33.72%	motif file (matrix)	svg
82		CUC3(NAC)/col-CUC3-DAP-Seq(GSE60143)/Homer	1e-2	-5.527e+00	0.0488	94.0	2.69%	6101.3	2.02%	motif file (matrix)	svg
83		Sox17(HMG)/Endoderm-Sox17-ChIP-Seq(GSE61475)/Homer	1e-2	-5.501e+00	0.0495	467.0	13.38%	35922.1	11.90%	motif file (matrix)	svg
84		Nkx6.1(Homeobox)/Islet-Nkx6.1-ChIP-Seq(GSE40975)/Homer	1e-2	-5.478e+00	0.0500	1576.0	45.17%	129635.2	42.94%	motif file (matrix)	svg
85		ANAC053(NAC)/colamp-ANAC053-DAP-Seq(GSE60143)/Homer	1e-2	-5.475e+00	0.0500	122.0	3.50%	8240.0	2.73%	motif file (matrix)	svg
86		Phox2b(Homeobox)/CLBGA-PHOX2B-ChIP-Seq(GSE90683)/Homer	1e-2	-5.454e+00	0.0500	154.0	4.41%	10718.8	3.55%	motif file (matrix)	svg
87		Dlx3(Homeobox)/Kerainocytes-Dlx3-ChIP-Seq(GSE89884)/Homer	1e-2	-5.432e+00	0.0506	525.0	15.05%	40743.3	13.50%	motif file (matrix)	svg
88		RBFox2(?)/Heart-RBFox2-CLIP-Seq(GSE57926)/Homer	1e-2	-5.365e+00	0.0534	778.0	22.30%	61863.6	20.49%	motif file (matrix)	svg
89		dof45(C2C2dof)/col-dof45-DAP-Seq(GSE60143)/Homer	1e-2	-5.355e+00	0.0534	1431.0	41.01%	117290.8	38.85%	motif file (matrix)	svg
90		MYB13(MYB)/col-MYB13-DAP-Seq(GSE60143)/Homer	1e-2	-5.316e+00	0.0549	212.0	6.08%	15331.3	5.08%	motif file (matrix)	svg
91		Atf4(bZIP)/MEF-Atf4-ChIP-Seq(GSE35681)/Homer	1e-2	-5.263e+00	0.0573	150.0	4.30%	10467.0	3.47%	motif file (matrix)	svg
92		AT5G25475(ABI3VP1)/col-AT5G25475-DAP-Seq(GSE60143)/Homer	1e-2	-5.260e+00	0.0573	405.0	11.61%	30963.7	10.26%	motif file (matrix)	svg
93		Tcfcp2l1(CP2)/mES-Tcfcp2l1-ChIP-Seq(GSE11431)/Homer	1e-2	-5.249e+00	0.0573	34.0	0.97%	1814.7	0.60%	motif file (matrix)	svg
94		AT3G12130(C3H)/colamp-AT3G12130-DAP-Seq(GSE60143)/Homer	1e-2	-5.198e+00	0.0592	1316.0	37.72%	107581.9	35.64%	motif file (matrix)	svg
95		AtIDD11(C2H2)/colamp-AtIDD11-DAP-Seq(GSE60143)/Homer	1e-2	-5.193e+00	0.0592	148.0	4.24%	10332.6	3.42%	motif file (matrix)	svg
96		At1g19000(MYBrelated)/colamp-At1g19000-DAP-Seq(GSE60143)/Homer	1e-2	-5.189e+00	0.0592	549.0	15.74%	42876.3	14.20%	motif file (matrix)	svg
97		SeqBias: C/A-bias	1e-2	-5.069e+00	0.0652	3481.0	99.77%	300310.8	99.48%	motif file (matrix)	svg
98		MYB83(MYB)/colamp-MYB83-DAP-Seq(GSE60143)/Homer	1e-2	-4.980e+00	0.0706	642.0	18.40%	50749.2	16.81%	motif file (matrix)	svg
99		Sox4(HMG)/proB-Sox4-ChIP-Seq(GSE50066)/Homer	1e-2	-4.977e+00	0.0706	390.0	11.18%	29885.3	9.90%	motif file (matrix)	svg
100		TCP7(TCP)/col-TCP7-DAP-Seq(GSE60143)/Homer	1e-2	-4.972e+00	0.0706	125.0	3.58%	8612.2	2.85%	motif file (matrix)	svg
101		CUC2(NAC)/colamp-CUC2-DAP-Seq(GSE60143)/Homer	1e-2	-4.896e+00	0.0745	83.0	2.38%	5421.2	1.80%	motif file (matrix)	svg
102		TCP20(TCP)/col-TCP20-DAP-Seq(GSE60143)/Homer	1e-2	-4.891e+00	0.0745	103.0	2.95%	6941.2	2.30%	motif file (matrix)	svg
103		Fos(bZIP)/TSC-Fos-ChIP-Seq(GSE110950)/Homer	1e-2	-4.879e+00	0.0745	221.0	6.33%	16220.8	5.37%	motif file (matrix)	svg
104		ZNF692(Zf)/HEK293-ZNF692.GFP-ChIP-Seq(GSE58341)/Homer	1e-2	-4.809e+00	0.0789	58.0	1.66%	3582.5	1.19%	motif file (matrix)	svg
105		TCP16(TCP)/colamp-TCP16-DAP-Seq(GSE60143)/Homer	1e-2	-4.744e+00	0.0834	134.0	3.84%	9381.5	3.11%	motif file (matrix)	svg
106		Knotted(Homeobox)/Corn-KN1-ChIP-Seq(GSE39161)/Homer	1e-2	-4.740e+00	0.0834	388.0	11.12%	29850.0	9.89%	motif file (matrix)	svg
107		Tbr1(T-box)/Cortex-Tbr1-ChIP-Seq(GSE71384)/Homer	1e-2	-4.691e+00	0.0863	553.0	15.85%	43525.9	14.42%	motif file (matrix)	svg
108		TRPS1(Zf)/MCF7-TRPS1-ChIP-Seq(GSE107013)/Homer	1e-2	-4.633e+00	0.0906	1005.0	28.80%	81581.1	27.02%	motif file (matrix)	svg
